# Design, Synthesis, Molecular Docking, and Anticancer Activity of Chalcone Derivatives as VEGFR-2 Inhibitors

**DOI:** 10.3390/molecules30234526

**Published:** 2025-11-24

**Authors:** Mingjun Yu, Xin Zhang, Hui Zhu, Xiaoqian Zhang

**Affiliations:** 1 School of Traditional Chinese Medicine, Bozhou University, Bozhou 236800, China; 2 School of Chinese Medicine, Hong Kong Baptist University, Kowloon, Hong Kong SAR 999077, China

**Keywords:** chalcone, VEGFR-2, urea fragment, anticancer, molecular docking

## Abstract

A series of 4-phenylurea chalcones (**2a**–**2s**) as VEGFR-2 inhibitors has been designed, synthesized, and evaluated for in vitro cytotoxic activity against K562, SiHa, and B16 cancer cells. Compared to sorafenib, the compounds exhibited strong cytotoxicity against K562, SiHa and B16 cells. Compounds **2r**, **2o**, and **2l** exhibited remarkable cytotoxicity against K562, SiHa, and B16 cells, with IC_50_ values of 0.97 μM, 1.22 μM, and 1.39 μM, respectively. Moreover, compound **2l** exhibited potent cytotoxicity against K562, SiHa, and B16, with IC_50_ values ranging from 1.25 μM to 1.39 μM. Compounds **2l** and **2o** also exhibited excellent inhibitory activity on VEGFR-2 kinase, with IC_50_ values of 0.42 ± 0.03 and 0.31 ± 0.02 μM, respectively. Molecular docking proved that the target compounds had strong binding interactions with VEGFR-2 proteins. Flow cytometry analysis showed that compound **2l** induced apoptosis and arrested the cell cycle at the G1 and S phases.

## 1. Introduction

Cancer is a complex disease that affects various organs and systems in the body [1]. It is one of the deadliest diseases that seriously endanger human life [2]. Angiogenesis plays a crucial role in cancer cell reproduction and growth [3,4]. The vascular endothelial growth factor (VEGF) signaling pathway is crucial for regulating cancer angiogenesis [5,6]. Vascular endothelial growth factor receptor (VEGFR) is a member of the receptor tyrosine kinase family, which includes VEGFR-1, VEGFR-2, and VEGFR-3 subtypes [7,8]. VEGFR-2 binds to VEGF to regulate physiological responses in vivo and plays an important role in the proliferation, migration, and survival of cancer cells [9,10,11]. High expression of VEGFR-2 in cancers promotes the formation and growth of blood vessels supplying tumors [12,13]. Therefore, inhibition of VEGFR-2 has emerged as an attractive cancer therapeutic strategy [14,15].

Small molecule VEGFR-2 inhibitors, such as sorafenib and regorafenib, have been approved by the FDA for the treatment of various cancers [16,17,18]. The urea functional group in sorafenib processes both hydrogen bond donors and acceptors, which form hydrogen bonds with key amino acid residues Glu885 and Asp1046 in the DFG (Asp-Phe-Gly) motif [19]. In addition, urea is frequently used in the development of anticancer drugs [20,21,22].

**Figure 1:**
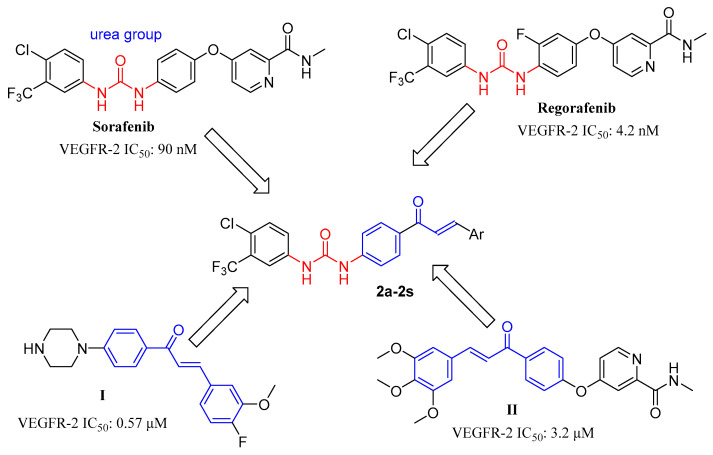
Structures of sorafenib, regorafenib, chalcone derivatives, and target compounds (**2a**–**2s**).

Chalcones belong to the flavonoid family and are commonly found in traditional Chinese medicines, vegetables, fruits, and other plants [23,24]. Chalcones and their derivatives exhibit various biological activities, including anticancer, anti-inflammatory, and antibacterial properties [25,26,27]. The α, β-unsaturated ketone structure of chalcone is considered crucial to its biological activity [28]. Chalcone has been synthesized in high yield from aldehydes and ketones via the Claisen–Schmid condensation reaction [29]. Owing to their various biological activities and simple chemistry, chalcone fragments are commonly used in drug design [30,31]. Chalcone derivatives as VEGFR-2 inhibitors have been reported (see Figure 1), such as compounds **I** and **II**; these compounds exhibited inhibition against cancer in vitro [31,32,33].

## 2. Results and Discussion

### 2.1. Synthesis of Compounds ***2a***–***2s***

**Scheme 1:**
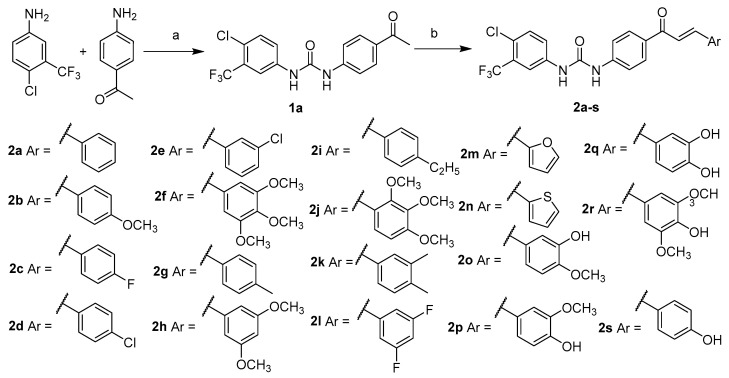
Synthesis of compounds (**2a**–**2s**). Reaction conditions: (a) triphosgene, Et_3_N, absolute DCM, N_2_, 0 °C, 2 h, 69.7%; (b) ArCHO, KOH, MeOH, r.t., overnight, 51.1–81.0%; or ArCHO, SOCl_2_, absolute EtOH, N_2_, 0 °C, 2 h, 46.3–69.8%.

The synthetic approach for the preparation of compounds **2a**–**2s** is illustrated in Scheme 1. 4-Aminoacetophenone was reacted with 4-chloro-3-(trifluoromethyl)phenyl isocyanate in the presence of triethylamine to form the intermediate 1a. Intermediate 1a was allowed to react with aromatic aldehydes to form chalcone derivatives **2a**–**2s** using potassium hydroxide or thionyl chloride as catalysts [34,35].

### 2.2. Biological Evaluation

#### 2.2.1. In Vitro Cytotoxic Activities Study

The CCK-8 assay was used to evaluate the cytotoxicity of compounds **2a**–**2s** in human cervical squamous cell carcinoma cell line SiHa, human chronic myeloid leukemia cell line K562, mouse melanoma cell line B16, and human renal epithelial cell line HK-2. Sorafenib was used as the positive control. The cytotoxic activities of the tested compounds are summarized in Table 1. Data analysis revealed that the benzene ring demonstrated strong cytotoxicity, whereas heteroaromatic compounds **2m** and **2n** exhibited low cytotoxicity. Substituents on the benzene ring exhibited significant impacts on cytotoxicity. The para-substituted compounds **2b**, **2c**, **2d**, **2g**, **2i**, and **2s** increased cytotoxicity against K562 cells, while for B16 cells, cytotoxicity was enhanced, except for chlorine substitution compound **2d**. However, only the fluorine-substituted compound **2c** increased cytotoxicity against SiHa cells. The multi-substituted compounds significantly enhanced cytotoxicity against K562, B16, and SiHa.

As illustrated in Table 1, compound **2c**, containing a 4-fluorine substitution, exhibited significant cytotoxicity against K562, B16, and SiHa cells, with IC_50_ values of 2.05 ± 0.13, 5.02 ± 0.18, and 1.03 ± 0.02 μM, respectively, and represented 6.2-, 2.1-, and 4.3-fold increases in cytotoxicity compared to the unsubstituted compound **2a**. Compared to compound **2c**, 3,5-difluoro-substituted compound **2l** increased cytotoxicity against K562 cells, with an IC_50_ value of 0.91 ± 0.07 μM, and enhanced inhibitory activity on B16, with an IC_50_ value of 1.39 ± 0.21 μM. The cytotoxicity of 3,5-dimethoxy-substituted compound **2h** and 3,4,5-trimethoxy-substituted compound **2f** against K562 and B16 cells was significantly higher than that of 4-methoxy-substituted compound **2b**, with IC_50_ values ranging from 1.62 to 2.02 μM. Introducing hydroxyl (**2q**) or methoxy groups (**2p**) into the 4-hydroxy-substituted compound **2s** increased its cytotoxicity against K562 cells, with IC_50_ values decreasing to 1.12 ± 0.10 and 3.42 ± 0.18 μM, respectively, from 6.47 ± 0.36 μM. Swapping the positions of the hydroxyl and methoxy groups in compound **2p** significantly increased its cytotoxicity, with IC_50_ values decreasing from 4.25 μM to 1.22 μM for SiHa cells, from 3.42 μM to 1.51 μM for K562 cells, and from 4.37 μM to 3.06 μM for B16 cells.

The cytotoxicity of synthesized compounds against HK-2 cells was also detected. As shown in Table 1, the target compound exhibited significantly lower cytotoxicity against HK-2 cells compared to SiHa, K562, and B16 cells. Compared to sorafenib, the compounds **2l**, **2o**, and **2q** exhibited low cytotoxicity, with IC_50_ values of 25.36 ± 1.47, 19.14 ± 1.05, and 24.32 ± 1.71 μM, respectively. In addition, compound **2l** also exhibited high selectivity toward SiHa, K562, and B16 cells, and represented 20.3-, 19.5, and 18.2-fold decreases in cytotoxicity, respectively.

#### 2.2.2. VEGFR-2 Enzyme Inhibition Assay Results

Many studies have shown that abnormal VEGFR-2 signaling could induce proliferation of cervical squamous cell carcinoma cells, myeloid leukemia cancer cells, and melanoma cells [36,37,38]. Targeting VEGFR-2 with inhibitor molecules has been considered an effective method for treating cervical cancer, myeloid leukemia, and melanoma [39,40,41,42]. The inhibitory effects of the synthesized compounds on VEGFR-2 were evaluated, with sorafenib used as a reference drug. The results are shown in Table 2. Compounds **2f**, **2l**, and **2o** exhibited excellent inhibitory activity on VEGFR-2 kinase, with IC_50_ values of 0.39 ± 0.02, 0.42 ± 0.03, and 0.31 ± 0.02 μM, respectively. Compound 2n showed low activity on VEGFR-2 kinase, with an IC_50_ value of 2.2 ± 0.1 μM. Although the activity of compound **2o** on VEGFR-2 kinase was lower than sorafenib, it is higher than reported VEGFR-2 inhibitors I (0.57 μM) and II (3.2 μM) containing chalcone structures [31,32]. These results indicate that the inhibitory activity of VEGR-2 kinase is consistent with its cytotoxicity against cancer cells.

#### 2.2.3. Compound **2l** Induced K562 Apoptosis

**Figure 2:**
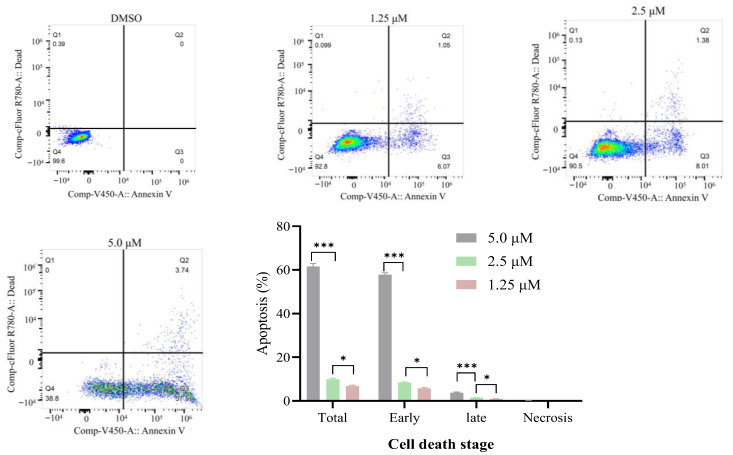
Compound **2l** induces apoptosis in K562 cells. Q1—necrotic cell content; Q2—late apoptotic; Q3—early apoptotic; Q4—viable cells. Values are expressed as an average of three independent replicates: Mean ± SD. * (*p* ≤ 0.05) and *** (*p* ≤ 0.001) indicate significant differences.

Enhancing apoptotic cell death is a crucial way for anticancer drugs to eliminate cancer cells [43,44]. Compound **2l** exhibited significant antiproliferative activity against K562, SiHa, and B16 cells. Therefore, compound **2l** was selected for the apoptosis study. The apoptosis and necrosis patterns of K562 cells were studied after treatment with compound **2l** at concentrations of 1.25, 2.5, and 5.0 μM. As shown in Figure 2, early apoptosis increased in a dose-dependent manner, reaching 5.80%, 8.34%, and 57.79% after treatment with 1.25, 2.5, and 5.0 μM of compound **2l**, respectively. Late apoptosis increased in a dose-dependent manner. Almost no necrosis of the K562 cells was observed. These results demonstrated that compound **2l** contributes to the induction of apoptosis in K562 cells.

#### 2.2.4. Compound **2l** Induced K562 Cycle Analysis

**Figure 3:**
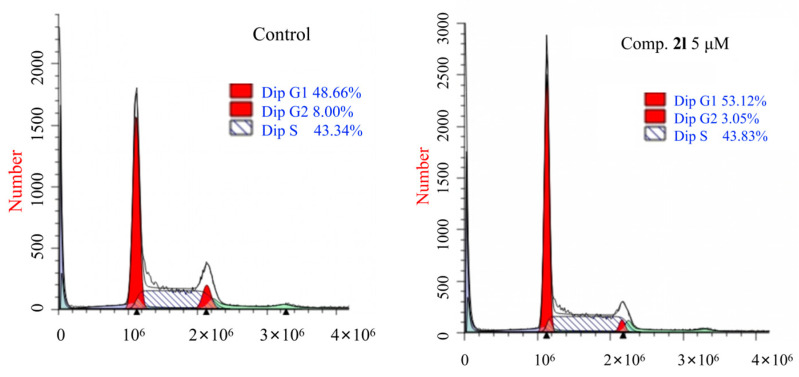
Effect of compound **2l** on the phases of the cell cycles of K562 cells.

Cytotoxic compounds often inhibit cell proliferation by arresting the cell cycle at a specific phase [44]. Flow cytometry was used to analyze the effect of compound **2l** on the cell cycle of K562 cells at a concentration of 5 μM, and the results are shown in Figure 3. Compared with DMSO treatment, the percentage of K562 cells in the G1 phase was increased from 48.66% to 53.12%, and those in the G2/M phase dropped from 8.00% to 3.05% after treatment with compound **2l**. On the other hand, the percentage of K562 cells in the S phase increased from 43.34% to 43.83%. These results showed that the K562 cell cycle was arrested in the G1 phase.

### 2.3. Molecular Docking Results

**Figure 4:**
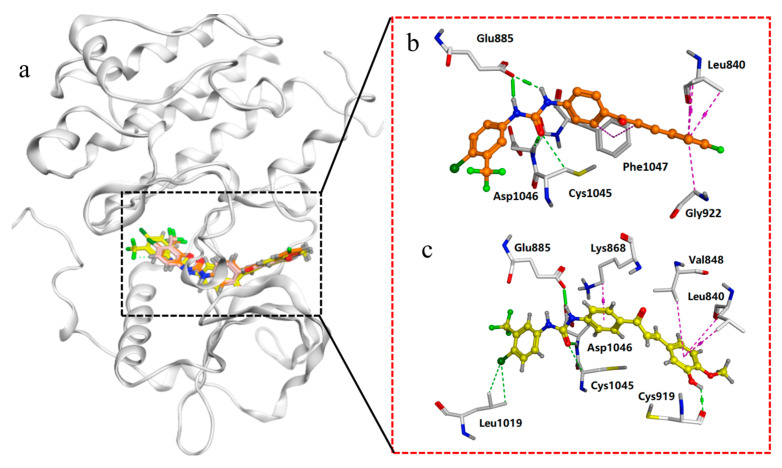
(**a**) Binding mode of compounds **2l** and **2o** with VEGFR-2 (PDB: 4ASD). (**b**) Display interaction between compound **2l** with amino acid residues. (**c**) Display interaction between compound **2o** with amino acid residues. Note: Green dashed line—H-bond; purple dashed line—arene-H interaction.

Molecular docking is an important tool for studying the molecular interactions of ligand-binding sites [45]. MOE (2019) was used to assess the interaction behaviors of compounds **2a**–**2s** with the active site of VEGFR-2 proteins, and sorafenib served as the control drug. Molecular docking was performed using a flexible ligand-docking protocol [46]. The binding free energies (ΔG) and interactions are presented in Table S1 and Figure 4. Sorafenib exhibited a binding affinity of −10.230 kcal/mol. In the sorafenib structure, the two N atoms, an O atom in the urea fragment, and an N atom in the pyridine fragment form hydrogen bonds with the amino acid residues Glu885, Asp1046, and Cys919, respectively. In addition, the amino acid residue Phe1047 forms hydrophobic interactions with the benzene ring. Compounds **2a**–**2s** exhibited binding free energies from −8.983 kcal/mol to −10.178 kcal/mol, and both formed hydrogen bonds with amino acid residues Glu885 and Asp1046. Compounds **2a**, **2c**, **2g**, **2h**, **2j**, **2l**, and **2n**–**2q** formed two hydrogen bonds with the amino acid residue Glu885. In additional, compound **2o** formed a hydrogen bond interaction with Cys919, pi-stacking and hydrophobic interactions with Leu1019, and arene-H interactions with Leu840, Val848, and Lys868. Compound **2l** formed arene-H interactions with Leu84o and Gly922. Compound **2f** exhibited higher binding free energy, but its inhibitory activity on VEGFR-2 kinase was lower than that of compound **2o**, which may be due to its lack of hydrogen bonds with amino acid residue Cys919, while **2o** formed hydrogen bonds with cys919. Compared to the benzene ring compounds, heteroaromatic compounds **2m** and **2n** decreased binding free energies and cytotoxicity. The substitution of methoxy groups on the benzene ring, followed by the introduction of additional substituents on the benzene ring, significantly enhanced affinity and cytotoxicity towards VEGFR-2 (**2b** vs. **2f**, **2h**, **2o**, **2p**, and **2r**). Compound **2c**, with a single fluorine substitution, exhibits lower affinity and cytotoxicity towards VEGFR-2 compared to compound **2l**, which has a double fluorine substitution.

## 3. Materials and Methods

### 3.1. Chemistry

All chemicals were purchased from Shanghai Aladdin Biochemical Technology Co., Ltd. (Shanghai, China). ^1^H NMR and ^13^C NMR spectra were obtained using a Bruker 600 MHz NMR spectrometer (Karlsruhe, Germany) at the Instrumental Analysis Center of HFUT, Hefei University of Technology, Hefei, China. Dimethyl sulfoxide (DMSO-*d*_6_) was used as the solvent, and TMS was used as the internal reference. The melting point was measured using HMZ-2B Micro Processor Melting Point apparatus (HUAZHI Electronic Technology Co., Ltd., Putian, Fujian, China) and was uncorrected. Mass spectra were measured with LC-MS Agilent 6200 ESI-qTOF (Agilent Technologies, Santa Clara, CA, USA). Thin-layer chromatography (TLC) was used to monitor reactions on HSGF254 silica gel plates.

#### 3.1.1. Procedure for Synthesis of Compound **1a**

4-aminoacetophenone (1.35 g, 10.0 mmol) and triethylamine (1.21 g, 12 mmol) were added to absolute dichloromethane (DCM, 40 mL) under a nitrogen atmosphere. The reaction mixture was cooled to 0 °C and a dichloromethane solution containing 4-chloro-3-(trifluoromethyl)phenyl isocyanate (2.21 g, 10.0 mmol) was added dropwise to the reaction mixture. The reaction mixture proceeded at this temperature for 2 h, was warmed to 20–25 °C, and was then stirred for an additional 2 h. The precipitate was then filtered and washed with dichloromethane. The cake was dried to produce a 2.49 g off-white solid as the product, with 69.7% yield, and was used in the next reaction without further purification.

#### 3.1.2. General Procedure for Preparation of **2a**–**2n**

Compound **1a** (0.178 g, 0.5 mmol), aldehyde (0.5 mmol), and potassium hydroxide (1.5 mmol) were added into methanol (5 mL). The reaction mixture was then stirred overnight at room temperature. Water (3 mL) was then added dropwise to the mixture and stirred for 1 h. The precipitate was filtered and recrystallized in ethanol/water to produce compounds **2a**–**2n**.

#### 3.1.3. General Procedure for Preparation of **2o**–**2s**

Absolute ethanol (10 mL) was cooled to −5 °C under a nitrogen atmosphere, and thionyl chloride (0.5 mL) was added when below 0 °C. Then, compound **1a** (0.178 g, 0.5 mmol) and aldehyde (0.55 mmol) were added, and the mixture was stirred at 0 °C for 2 h. The reaction was then quenched with water. The precipitate was filtered and recrystallized in ethanol/water to produce compounds **2o**–**2s**.

#### 3.1.4. Structural Characterization of Synthesized Compounds

1-(4-acetylphenyl)-3-(4-chloro-3-(trifluoromethyl)phenyl)urea (**1a**): Off-white solid, m.p. 247.2–248.2 °C. ^1^H NMR (600 MHz, DMSO-*d*_6_) δ 9.28 (d, J = 4.6 Hz, 2H), 8.11 (d, J = 2.3 Hz, 1H), 7.91 (d, J = 8.7 Hz, 2H), 7.64 (dt, J = 16.6, 5.6 Hz, 2H), 7.60 (d, J = 8.7 Hz, 2H), 2.52 (s, 3H).

1-(4-chloro-3-(trifluoromethyl)phenyl)-3-(4-cinnamoylphenyl)urea (**2a**): Yellow powder, yield 73.2%, m.p. 204.2–205.7 °C. ^1^H NMR (600 MHz, DMSO-*d*_6_) δ 9.32 (s, 1H), 9.30 (s, 1H), 8.15 (d, J = 8.8 Hz, 2H,), 8.12 (d, J = 2.4 Hz, 1H), 7.94 (d, J = 15.6 Hz, 1H), 7.88 (dd, J = 7.3, 1.9 Hz, 2H), 7.72 (d, J = 15.6 Hz, 1H), 7.69–7.61 (m, 4H), 7.49–7.42 (m, 3H); ^13^C NMR (151MHz, DMSO-*d*_6_) δ 187.37, 152.14, 144.02, 143.31, 139.01, 134.83, 132.08, 131.39, 130.47, 130.10, 128.93, 128.82, 126.76 (q, J = 31.1 Hz), 123.38, 122.81 (q, J = 273.2 Hz), 122.75, 121.99, 117.71, 117.04 (q, J = 5.6 Hz). HR-MS (*m*/*z*), [M + H]^+^: 445.0925, found: 445.0931.

(E)-1-(4-chloro-3-(trifluoromethyl)phenyl)-3-(4-(3-(4-methoxyphenyl)acryloyl)phenyl)urea (**2b**): Yellow powder, yield 81.0%, m.p. 220.6–222.5 °C. ^1^H NMR (600 MHz, DMSO-*d*_6_) δ 9.35 (s, 2H), 8.15–8.12 (m, 3H), 7.84 (d, J = 8.7 Hz, 2H), 7.80 (d, J = 15.5 Hz, 1H), 7.70 (s, 1H), 7.68–7.58 (m, 4H), 7.01 (d, J = 8.7 Hz, 2H), 3.82 (s, 3H); ^13^C NMR (151MHz, DMSO-*d*_6_) 187.24, 161.22, 152.14, 143.81, 143.14, 139.02, 132.03, 131.65, 130.64, 129.89, 127.46, 126.64 (q, J = 30.5 Hz), 123.33, 122.79 (q, J = 273.3 Hz), 122.69, 119.46, 117.66, 117.01 (q, J = 5.7 Hz), 114.38, 51.36. HR-MS (*m*/*z*), [M-H]^−^: 473.0885, found: 473.0870.

(E)-1-(4-chloro-3-(trifluoromethyl)phenyl)-3-(4-(3-(4-fluorophenyl)acryloyl)phenyl)urea (**2c**): Off-white powder, yield 62.7%, m.p. 211.6–212.6 °C. ^1^H NMR (600 MHz, DMSO-*d*_6_) δ 9.37 (s, 2H), 8.14 (d, J = 8.7 Hz, 2H), 8.12 (d, J = 2.2 Hz, 1H), 7.96 (dd, J = 8.4, 5.8 Hz, 2H), 7.91 (d, J = 15.6 Hz, 1H), 7.72 (d, J = 15.6 Hz, 1H), 7.69–7.60 (m, 4H), 7.30 (t, J = 8.8 Hz, 2H); ^13^C NMR (151MHz, DMSO-*d*_6_) δ 187.27, 163.31 (d, J = 248.7 Hz), 152.14, 144.05, 141.92, 139.01, 133.03, 131.51(J = 3.1Hz), 131.35, 131.11 (d, J = 8.4Hz), 130.06, 126.74 (q, J = 30.5 Hz), 123.35, 122.79 (q, J = 273.3 Hz) 122.72,121.89, 117.67, 117.03 (q, J = 5.7 Hz), 115.90 (d, J = 21.6 Hz). HR-MS (*m*/*z*), [M-H]^−^: 461.0685, found: 461.0690.

(E)-1-(4-chloro-3-(trifluoromethyl)phenyl)-3-(4-(3-(4-chlorophenyl)acryloyl)phenyl)urea (**2d**): Off-white powder, yield 80.3%, m.p. 230.1–231.5 °C. ^1^H NMR (600 MHz, DMSO-*d*_6_) δ 9.38 (s, 2H), 8.15 (d, J = 8.7 Hz, 2H), 8.11 (d, J = 2.0 Hz, 1H), 7.96 (d, J = 15.6 Hz, 1H), 7.92 (d, J = 8.4 Hz, 2H), 7.70 (d, J = 15.8 Hz, 1H), 7.66–7.62 (m, 4H), 7.52 (d, J = 8.4 Hz, 2H); ^13^C NMR (151MHz, DMSO-*d*_6_) δ 187.22, 152.12, 144.12, 141.67, 139.00, 134.89, 133.81, 132.03, 131.25, 130.47, 130.10, 128.92, 126.74 (q, J = 30.5 Hz), 123.69, 123.34, 122.78 (J = 273.2 Hz), 122.74, 117.67, 117.02 (J = 5.7 Hz). HR-MS (*m*/*z*), [M + H]^+^: 479.0535, found, 479.0519.

(E)-1-(4-chloro-3-(trifluoromethyl)phenyl)-3-(4-(3-(3-chlorophenyl)acryloyl)phenyl)urea (**2e**): Off-white powder, yield 51.1%, m.p. 245.1–246.7 °C. ^1^H NMR (600 MHz, DMSO-*d*_6_) δ 9.33 (s, 1H), 9.31 (s, 1H), 8.17 (d, J = 8.8 Hz, 2H), 8.11 (d, J = 2.5 Hz, 1H), 8.07 (s, 1H), 8.03 (d, J = 15.6 Hz, 1H), 7.82~7.79 (m, 1H), 7.71–7.60 (m, 5H), 7.51–7.45 (m, 2H); ^13^C NMR (151MHz, DMSO-*d*_6_) δ 187.20, 152.10, 144.16, 141.45, 138.96, 137.11, 133.80, 132.05, 131.21, 130.66, 130.21, 129.96, 127.86, 127.81, 126.75 (q, J = 30.8 Hz), 123.53, 123.37, 122.80 (q, J = 273.2 Hz), 122.76, 117.67, 117.05 (q, J = 5.8Hz). HR-MS (*m*/*z*), [M − H]^−^: 477.0389, found: 477.0382.

(E)-1-(4-chloro-3-(trifluoromethyl)phenyl)-3-(4-(3-(3,4,5-trimethoxyphenyl)acryloyl)phenyl)urea (**2f**): Off-white powder, yield 76.6%, m.p. 228.0–229.8 °C. ^1^H NMR (600 MHz, DMSO-*d*_6_) δ 9.34 (s, 2H), 8.16 (d, J = 8.3 Hz, 2H), 8.11 (d, J = 2.1 Hz, 1H), 7.89 (d, J = 15.5 Hz, 1H), 7.72~7.60 (m, 5H), 7.22 (s, 2H), 3.87 (s, 6H_3_), 3.72 (s, 3H); ^13^C NMR (151MHz, DMSO-*d*_6_) δ 187.31, 153.10, 152.13, 143.96, 143.69, 139.60, 139.00, 132.05, 131.50, 130.38, 130.03, 126.75 (q, J = 30.6 Hz), 123.36, 122.79 (q, J = 273.6 Hz), 122.73, 121.16,117.65, 117.03 (q, J = 5.6 Hz), 106.44, 60.14, 56.13. HR-MS (*m*/*z*), [M − H]^−^:533.1096, found: 533.1099.

(E)-1-(4-chloro-3-(trifluoromethyl)phenyl)-3-(4-(3-(p-tolyl)acryloyl)phenyl)urea (**2g**): Off-white powder, yield 69.9%, m.p. 201.8–202.6 °C. ^1^H NMR (600 MHz, DMSO-*d*_6_) δ 9.36 (s, 2H), 8.13 (dd, J = 10.0, 5.6 Hz, 3H), 7.88 (d, J = 15.6 Hz, 1H), 7.77 (d, J = 8.0 Hz, 2H), 7.72–7.59 (m, 5H), 7.27 (d, J = 7.9 Hz, 2H), 2.35 (s, 3H); ^13^C NMR (151MHz, DMSO-*d*_6_) δ 187.32, 152.13, 143.94, 143.23, 140.45, 139.02, 132.10, 132.03, 131.48, 129.98, 129.52, 128.80, 126.74 (q, J = 30.4 Hz), 123.34, 122.79 (q, J = 273.5 Hz), 122.70, 120.91, 117.67, 117.02 (q, J = 5.6 Hz), 21.08. HR-MS (*m*/*z*), [M−H]^−^: 457.0936, found: 457.0940.

(E)-1-(4-chloro-3-(trifluoromethyl)phenyl)-3-(4-(3-(3,5-dimethoxyphenyl)acryloyl)phenyl)urea (**2h**): Off-white powder, yield 73.3%, m.p. 244.7–246.2 °C. ^1^H NMR (600 MHz, DMSO-*d*_6_) δ 9.37 (s, 2H), 8.16 (d, J = 8.5 Hz, 2H), 8.11 (d, J = 2.0 Hz, 1H), 7.94 (d, J = 15.5 Hz, 1H), 7.72–7.58 (m, 4H), 7.06 (s, 2H), 6.58 (s, 1H), 3.81 (s, 6H). ^13^C NMR (151MHz, DMSO-*d*_6_) δ 187.38, 160.72, 152.15, 144.10, 143.34, 139.02, 136.77, 132.06, 131.34, 130.15, 126.76 (q, J = 30.6 Hz),123.37, 122.81 (q, J = 273.3 Hz), 122.73, 122.46, 117.67, 117.04 (q, J = 5.5 Hz), 106.66, 102.72, 55.47, 55.45. HR-MS (*m*/*z*), [M + H]^+^: 505.1136, found: 505.1132.

(E)-1-(4-chloro-3-(trifluoromethyl)phenyl)-3-(4-(3-(4-ethylphenyl)acryloyl)phenyl)urea (**2i**): Off-white powder, yield 61.4%, m.p. 242.5–244.2 °C. ^1^H NMR (600 MHz, DMSO-*d*_6_) δ 9.31 (s, 2H), 8.14 (d, J = 8.8 Hz, 2H), 8.12 (d, J = 2.5 Hz, 1H), 7.88 (d, J = 15.6 Hz, 1H), 7.78 (d, J = 8.1 Hz, 2H), 7.70 (d, J = 15.6 Hz, 1H), 7.68–7.60 (m, 4H), 7.29 (d, J = 8.0 Hz, 2H), 2.64 (q, J = 7.6 Hz, 2H), 1.19 (t, J = 7.6 Hz, 3H); ^13^C NMR (151 MHz, DMSO-*d*_6_) δ 187.36, 152.13, 146.71, 143.94, 143.28, 139.00, 132.38, 132.05, 131.51, 130.01, 128.92, 128.36, 126.77 (q, J = 30.6 Hz), 123.36, 122.81 (q, J = 273.3Hz), 122.76, 120.99, 117.70, 117.04 (q, J = 5.5 Hz). HR-MS (*m*/*z*), [M + H]^+^: 473.1238, found: 473.1257.

(E)-1-(4-chloro-3-(trifluoromethyl)phenyl)-3-(4-(3-(2,3,4-trimethoxyphenyl)acryloyl)phenyl)urea (**2j**): Off-white powder, yield 51.4%, m.p. 213.6–215.3 °C. ^1^H NMR (600 MHz, DMSO-*d*_6_) δ 9.33 (s, 1H), 9.31 (s, 1H), 8.14–8.11 (m, 2H), 8.10 (s, 1H, 7.87–7.83 (m, 2H), 7.77 (d, J = 8.8 Hz, 1H), 7.69~7.61 (m, 4H), 6.92 (d, J = 8.9 Hz, 1H), 3.87 (s, 6H), 3.78 (s, 3H); ^13^C NMR (151MHz, DMSO-*d*_6_) δ 187.37, 155.62, 153.03, 152.13, 143.82, 141.78, 139.01, 137.63, 132.06, 131.67, 129.89, 126.75 (q, J = 30.5 Hz), 123.35, 122.80 (q, J = 273.3 Hz), 122.73, 121.15, 120.39, 117.70, 117.02 (q, J = 5.5 Hz), 108.47, 61.50, 60.47, 56.06. HR-MS (*m*/*z*), [M − H]^−^: 533.1096, found: 533.1099.

(E)-1-(4-chloro-3-(trifluoromethyl)phenyl)-3-(4-(3-(3,4-dimethylphenyl)acryloyl)phenyl)urea (**2k**): Off-white powder, yield 64.5%, m.p. 253.8–255.6 °C. ^1^H NMR (600 MHz, DMSO-*d*_6_) δ 9.32 (s, 1H), 9.30 (s, 1H), 8.14 (d, J = 8.6 Hz, 2H), 8.12 (d, J = 2.2 Hz, 1H), 7.87 (d, J = 15.5 Hz, 1H), 7.68–7.63 (m, 6H), 7.57 (d, J = 7.8 Hz, 1H), 7.22 (d, J = 7.8 Hz, 1H), 2.27 (s, 3H), 2.26 (s, 3H); ^13^C NMR (151MHz, DMSO-*d*_6_) δ 187.30, 152.12, 143.90, 143.46, 139.36, 138.99, 136.86, 132.41, 132.05, 130.01, 129.98, 129.61, 129.59, 126.74 (q, J = 32.8 Hz), 123.36, 122.80 (q, J = 273.5 Hz), 122.73, 120.67, 117.67, 117.53, 117.03 (q, J = 5.5 Hz), 19.46, 19.29. HR-MS (*m*/*z*), [M + H]^+^: 473.1238, found: 473.1240.

(E)-1-(4-chloro-3-(trifluoromethyl)phenyl)-3-(4-(3-(3,5-difluorophenyl)acryloyl)phenyl)urea (**2l**): Off-white powder, yield 45.8%, m.p. 267.7–269.4 °C. ^1^H NMR (600 MHz, DMSO-*d*_6_) δ 9.33 (s, 1H), 9.31 (s, 1H), 8.17 (d, J = 8.7 Hz, 2H), 8.11 (d, J = 2.4 Hz, 1H), 8.06 (d, J = 15.6 Hz, 1H), 7.71 (d, J = 6.8 Hz, 2H), 7.69–7.61 (m, 5H), 7.31 (t, J = 9.1 Hz, 1H); ^13^C NMR (151MHz, DMSO-*d*_6_) δ 187.11, 162.65 (dd, J = 245.9, 13.3 Hz), 152.09, 144.28, 140.48, 138.95, 138.69 (t, J = 9.8 Hz), 132.05, 131.06, 130.27, 126.75 (d, J = 30.3 Hz), 124.71, 123.38, 122.79 (q, J = 273.5 Hz), 122.78, 117.67, 117.05 (d, J = 5.8 Hz), 111.68 (dd, J = 20.4, 5.2 Hz), 105.35 (t, J = 26.2 Hz). HR-MS (*m*/*z*), [M + H]^+^: 481.0737, found: 481.0744.

(E)-1-(4-chloro-3-(trifluoromethyl)phenyl)-3-(4-(3-(furan-2-yl)acryloyl)phenyl)urea (**2m**): Yellow powder, yield 71.3%, m.p. 207.8–209.9 °C. ^1^H NMR (600 MHz, DMSO-*d*_6_) δ 9.36 (s, 2H), 8.19 (s, 1H), 8.11 (d, J = 2.4 Hz, 1H), 8.09 (d, J = 8.7 Hz, 2H), 7.78 (s, 1H), 7.69–7.61 (m, 6H), 7.15 (d, J = 1.2 Hz, 1H); ^13^C NMR (151MHz, DMSO-*d*_6_) δ 187.23, 152.14, 145.56, 144.94, 143.92, 139.01, 132.75, 132.05, 131.36, 129.90, 126.76 (q, J = 30.3 Hz), 123.35, 123.30, 122.80 (q, J = 273.3 Hz),122.74, 121.62, 117.61, 117.02 (q, J = 5.9 Hz), 108.71. HR-MS (*m*/*z*), [M − H]^−^: 433.0572, found: 433.0577.

(E)-1-(4-chloro-3-(trifluoromethyl)phenyl)-3-(4-(3-(thiophen-2-yl)acryloyl)phenyl)urea (**2n**): Off-white powder, yield 72.1%, m.p. 205.4–206.1 °C. ^1^H NMR (600 MHz, DMSO-*d*_6_) δ 9.32 (s, 1H), 9.31 (s, 1H), 8.12 (dd, J = 5.7, 3.1 Hz, 3H), 8.08 (dd, J = 2.8, 1.0 Hz, 1H), 7.79–7.73 (m, 3H), 7.69–7.62 (m, 5H); ^13^C NMR (151 MHz, DMSO-*d*_6_) δ 187.59,152.13, 143.91, 138.99, 138.35, 137.05, 132.06, 131.48, 130.25, 129.95, 127.64, 126.76 (q, J = 30.5 Hz), 126.17, 123.36, 122.80 (q, J = 273.3 Hz), 122.74, 121.46, 117.68, 117.03 (q, J = 5.6 Hz). HR-MS (*m*/*z*), [M − H]^−^: 449.0343, found: 449.0336.

(E)-1-(4-chloro-3-(trifluoromethyl)phenyl)-3-(4-(3-(3-hydroxy-4-methoxyphenyl)acryloyl)phenyl)urea (**2o**): Brown powder, yield 66.6%, m.p. 255.5–257.3 °C. ^1^H NMR (600 MHz, DMSO-*d*_6_) δ 9.29 (s, 2H), 9.15 (s, 1H), 8.11 (d, J = 8.6 Hz, 3H), 7.71–7.58 (m, 6H), 7.32 (s, 1H), 7.28 (d, J = 8.4 Hz, 1H), 6.99 (d, J = 8.3 Hz, 1H), 3.84 (s, 3H); ^13^C NMR (151 MHz, DMSO-*d*_6_) δ 187.24, 152.13, 150.17, 146.66, 143.75, 143.68, 139.01, 132.06, 131.72, 129.88, 127.77, 126.76 (q, J = 30.5 Hz), 123.35, 122.81 (q, J = 273.3 Hz),122.75, 121.92, 119.33, 117.69, 117.03 (q, J = 5.8 Hz), 114.80, 119.91, 55.69. HR-MS (*m*/*z*), [M + H]^+^: 491.0980, found: 491.0967.

(E)-1-(4-chloro-3-(trifluoromethyl)phenyl)-3-(4-(3-(4-hydroxy-3-methoxyphenyl)acryloyl)phenyl)urea (**2p**): Brown powder, yield 69.8%, m.p. 251.3–252.9 °C. ^1^H NMR (600 MHz, DMSO-*d*_6_) δ 9.66 (s, 1H), 9.30 (s, 2H), 8.14 (d, J = 8.8 Hz, 2H), 8.12 (d, J = 2.4 Hz, 1H), 7.77 (d, J = 15.4 Hz, 1H), 7.70–7.62 (m, 5H), 7.51 (d, J = 1.7 Hz, 1H), 7.27 (dd, J = 8.2, 1.8 Hz, 1H), 6.84 (d, J = 8.1 Hz, 1H), 3.88 (s, 3H); ^13^C NMR (151MHz, DMSO-*d*_6_) δ 187.22, 152.13, 149.54, 147.98, 144.10, 143.70, 139.01, 132.05, 131.80, 129.87, 126.75 (q, J = 30.8 Hz), 126.41, 123.99, 123.35, 122.80 (q, J = 273.2 Hz), 122.71, 118.56, 117.64, 117.02 (q, J = 5.9 Hz), 115.57, 111.57, 55.82. HR-MS (*m*/*z*), [M − H]^−^: 489.0834, found: 489.0839.

(E)-1-(4-chloro-3-(trifluoromethyl)phenyl)-3-(4-(3-(3,4-dihydroxyphenyl)acryloyl)phenyl)urea (**2q**): Brown powder, yield: 53.9%, m.p. 219.6–221.3 °C. ^1^H NMR (600 MHz, DMSO-*d*_6_) δ 9.68 (s, 1H), 9.31 (s, 1H), 9.30 (s, 1H), 9.11 (s, 1H), 8.12 (d, J = 2.1 Hz, 1H), 8.09 (d, J = 8.6 Hz, 2H), 7.70–7.53 (m, 6H), 7.25 (d, J = 1.4 Hz, 1H), 7.20–7.15 (m, 1H), 6.81 (d, J = 8.1 Hz, 1H); ^13^C NMR (151MHz, DMSO-*d*_6_) δ 187.21, 152.14, 148.16, 145.59, 144.12, 143.65, 139.02, 132.06, 131.84, 129.79, 126.76 (q, J = 30.7 Hz), 126.42, 123.34, 122.80 (q, J = 270.0 Hz), 122.71, 122.02, 118.31, 117.68, 117.01 (d, J = 5.6 Hz), 115.74, 115.48. HR-MS (*m*/*z*), [M + H]^+^: 477.0823, found: 477.0808.

(E)-1-(4-chloro-3-(trifluoromethyl)phenyl)-3-(4-(3-(4-hydroxy-3,5-dimethoxyphenyl)acryloyl)phenyl)urea (**2r**): Brown powder, yield 69.3%, m.p. 196.9–198.4 °C. ^1^H NMR (600 MHz, DMSO-*d*_6_) δ 9.34 (s, 1H), 9.32 (s, 1H), 9.02 (s, 1H), 8.15 (d, J = 8.8 Hz, 2H), 8.11 (d, J = 2.5 Hz, 1H), 7.80 (d, J = 15.4 Hz, 1H), 7.69–7.62 (m, 5H), 7.19 (s, 2H), 3.85 (s, 6H); ^13^C NMR (151 MHz, DMSO-*d*_6_) δ 187.21, 152.17, 148.08, 144.51, 143.76, 139.04, 138.58, 132.07, 131.79, 129.93, 126.77 (q, J = 30.5 Hz),125.21, 123.37, 122.82 (q, J = 273.2 Hz), 122.72, 118.93, 117.65, 117.04 (q, J = 5.4 Hz), 106.84, 56.20. HR-MS (*m*/*z*), [M − H]^−^: 519.09403, found: 519.0933.

(E)-1-(4-chloro-3-(trifluoromethyl)phenyl)-3-(4-(3-(4-hydroxyphenyl)acryloyl)phenyl)urea (**2s**): Brown powder, yield 46.3%, m.p. 246.9–248.6 °C. ^1^H NMR (600 MHz, DMSO-*d*_6_) δ 10.06 (s, 1H), 9.31 (s, 1H), 9.30 (s, 1H), 8.13–8.09 (m, 3H), 7.72 (dd, J = 12.0, 3.3 Hz, 3H), 7.70–7.61 (m, 5H), 6.84 (d, J = 8.6 Hz, 2H); ^13^C NMR (151MHz, DMSO-*d*_6_) δ 187.24, 159.97, 152.13, 143.69, 143.66, 139.01, 132.05, 131.79, 130.87, 129.83, 126.76 (q, J = 30.7 Hz), 125.93, 123.34, 122.80 (q, J = 273.3 Hz), 122.72, 118.41, 117.67, 117.02 (q, J = 5.5 Hz), 115.81. HR-MS (*m*/*z*), [M − H]^−^: 459.07283, found: 459.0734.

### 3.2. In Vitro Cytotoxicity

The SiHa, K562, B16 and HK-2 cells were obtained from Wuhan Pricella Biotechnology Co., Ltd. (Wuhan, China). The in vitro cytotoxicity of compounds **2a**–**2s** against SiHa, K562, B16, and HK-2 cells was detected by CCK-8 assay, following the method detailed in reference [47]. Briefly, cells were cultured in RPMI-1640 medium containing 10% fetal bovine serum and 1% double antibody (penicillin–streptomycin) in a cell culture incubator at 37 °C and 5% CO_2_. 2–5 × 10^3^ cells were added to each well of a 96-well plate and incubated for 12 h, followed by the addition of the tested compounds. After incubation for 48 h, 10 μL of the CCK-8 solution was added to each well and incubated for another 2 h. The absorbance of each well was measured at 450 nm using an enzyme-linked immunosorbent assay (ELISA) reader. The experiments were replicated three times. The inhibition rate of cancer cells was calculated using the Formula (1). The half-maximal inhibitory concentration (IC_50_) value was calculated using GraphPad Prism 8.0 software.

(1) Inhibition rate (%) = (1 − ODtest)/ODcontrol × 100

OD_test_—absorbance of test sample; OD_control_—absorbance of control.

### 3.3. VEGFR-2 Enzyme Inhibition Assay

A VEGFR-2 (KDR) Kinase Assay Kit (BPS Biosciences, San Diego, CA, USA) was used to conduct a VEGFR-2 inhibition assay following the manufacturer’s protocol. Briefly, kinase buffer and ATP and PTK substrate (Poly-Glu,Tyr 4:1) were diluted with distilled water to prepare a master mix solution. The master mix solution, test sample, and VEGFR-2 (KDR) protein kinase (2 ng/μL) were added to a 96-well plate and incubated at 30 °C for 45 min. After incubation, Kinase-Glo™ MAX (BPS Biosciences) reagent was added to each well, and incubated for 15 min at room temperature. Then it was immediately detected with an ELISA reader. The experiments were replicated three times.

### 3.4. Cell Apoptosis Assay

A RayBio Annexin V Apoptosis Detection Kit (RayBright, Violet 450, RayBiotech Co., Ltd., Guangzhou, China) was used to assess the apoptotic effect of compound **2l** on K562 cells, following the methodology described in previous studies [48]. Briefly, cells were cultured in a 6-well plate for 12 h, and then compound **2l** was added, followed by further culturing for 24 h. The sample was centrifuged to isolate the cells and the kit instructions were followed for subsequent processing. Finally, apoptosis was quantified using a flow cytometer (Cytek Northern Light 3 Laser 16V-14B-8R, Wuxi, China). The experiments were replicated three times.

### 3.5. Cell Cycle Analysis

The effect of compound **2l** on the K562 cell cycle was assessed using propidium iodide (PI) staining on a flow cytometer according to a previously reported method [48]. Briefly, K562 cells were treated with compound 2l and cultured for 24 h. Cells were harvested and fixed with 75% ethanol at 4 °C overnight. The sample was then centrifuged (2000 rpm for 5 min), and the supernatant was aspirated. The fixed cells were resuspended in 0.5 mL of PI solution, cultured at 37 °C in the dark for 30 min, and then cultured at 4 °C in the dark for another 30 min Finally, the cell cycle sample was determined with a flow cytometer (Cytek Northern Light 3 laser 16V-14B-8R, Wuxi, China). The experiments were replicated three times.

### 3.6. Molecular Docking

Docking studies were conducted on compounds to explore their binding mode with VEGFR-2 proteins (PDB: 4ASD, resolution 2.03 Å) using MOE2019 software [49]. The proteins and compounds underwent energy minimization prior to molecular docking. Molecular docking was performed using a flexible docking approach, with the original ligand serving as the center. The London dG scoring function was employed for protein-binding affinity, with lower scores indicating stronger binding. Sorafenib was used as a control to verify the accuracy of the docking results.

### 3.7. Statistical Analysis

All the experiments were executed in triplicate, and experimental data were expressed as the mean ± standard deviation (SD), *n* = 3. The data were analyzed using analysis of variance (ANOVA), followed by suitable post-hoc tests, with *p* < 0.05 considered statistically significant. GraphPad Prism 8.0 was used to calculate IC_50_ values and create graphs.

## 4. Conclusions

A series of novel chalcone derivatives were designed and synthesized as VEGFR-2 inhibitors and their anti-proliferative activities against K562, SiHa, and B16 cells were evaluated. Most compounds, such as **2h**, **2l**, **2o**, and **2r**, exhibited strong antiproliferative activity compared with the reference drug sorafenib. Compound **2l** exhibited the most potent inhibitory activity against SiHa, K562, and B16 cells, with IC_50_ values of 1.25 ± 0.13, 1.30 ± 0.07, and 1.39 ± 0.21 μM, respectively. Moreover, the flow cytometer analysis revealed that the compound **2l** significantly promoted early and late apoptosis in k562 cells, and arrested the cell cycle of k562 cells at the G1 phase. Inhibition activity testing against VEGFR-2 kinase revealed that compounds **2l** and **2o** exhibited excellent inhibitory effects, with IC_50_ values of 0.42 ± 0.03 and 0.31 ± 0.02 μM, respectively. Furthermore, molecular docking studies also demonstrated that compounds **2l**, **2o**, and **2r** formed strong binding interactions with VEGFR-2 kinase. These results indicate that introducing chalcone fragments into the design of VEGFR-2 inhibitors is viable.

## 5. Patents

Mingjun Yu, Hui Zhu, and Xiaoqian Zhang, et al. Preparation method and application of a chalcone derivative-containing urea structure. 2025102951067, 13 March 2025.

## Figures and Tables

**Table 1:** Cytotoxicity of compounds **2a**–**2s** against SiHa, K562, B16, and HK-2 Cells.

Comp.	IC_50_, μM			
	SiHa	K562	B16	HK-2
**2a**	4.43 ± 0.16	12.65 ± 0.45	10.69 ± 0.78	nd
**2b**	5.55 ± 0.26	5.08 ± 0.31	8.32 ± 0.61	nd
**2c**	1.03 ± 0.02	2.05 ± 0.13	5.02 ± 0.18	15.68 ± 1.22
**2d**	4.78 ± 0.14	8.41 ± 0.57	12.77 ± 0.84	nd
**2e**	4.71 ± 0.29	2.91 ± 0.17	11.28 ± 0.64	nd
**2f**	4.89 ± 0.21	1.68 ± 0.09	1.65 ± 0.29	5.85 ± 0.36
**2g**	6.03 ± 0.38	4.19 ± 0.26	7.15 ± 0.49	nd
**2h**	2.99 ± 0.27	2.02 ± 0.12	1.62 ± 0.14	9.87± 0.89
**2i**	7.96 ± 0.26	4.45 ± 0.53	8.66 ± 0.58	nd
**2j**	6.62 ± 0.28	2.40 ± 0.22	9.73 ± 0.98	nd
**2k**	8.35 ± 0.09	1.73 ± 0.23	6.42 ± 0.54	nd
**2l**	1.25 ± 0.13	1.30 ± 0.07	1.39 ± 0.21	25.36 ± 1.47
**2m**	6.53 ± 0.24	8.95 ± 0.76	nd	nd
**2n**	>20	15.2 ± 1.2	nd	nd
**2o**	1.22 ± 0.07	1.51 ± 0.16	3.06 ± 0.22	19.14± 1.05
**2p**	4.25 ± 0.14	3.42 ± 0.18	4.37 ± 0.28	13.89± 0.58
**2q**	2.53 ± 0.09	1.12 ± 0.10	4.49 ± 0.24	24.32± 1.71
**2r**	2.22 ± 0.10	0.97 ± 0.06	2.93 ± 0.29	11.27± 0.66
**2s**	8.96 ± 0.03	6.47 ± 0.36	4.29 ± 0.52	nd
sorafenib	12.82 ± 0.71	3.34 ± 0.22	12.57 ± 1.03	10.87 ± 0.69

nd—not determined.

**Table 2:** Activity of targeted compounds on VEGFR-2 kinase.

Comp.	IC_50_, μM
**2c**	0.68 ± 0.03
**2f**	0.39 ± 0.02
**2l**	0.42 ± 0.03
**2n**	2.2 ± 0.1
**2o**	0.31 ± 0.02
**2q**	0.55 ± 0.04
sorafenib	0.12 ± 0.01

## Data Availability

The original contributions presented in this study are included in the article/Supplementary Materials. Further inquiries can be directed to the corresponding authors.

## Supplementary Materials

The following supporting information can be downloaded at https://www.mdpi.com/article/10.3390/molecules30234526/s1.

## Abbreviations

The following abbreviations are used in this manuscript:

VEGF	Vascular endothelial growth factor
VEGFR	Vascular endothelial growth factor receptor
VEGFR-1	Vascular endothelial growth factor receptor-1
VEGFR-2	Vascular endothelial growth factor receptor-2
VEGFR-3	Vascular endothelial growth factor receptor-3
FDA	Food and Drug Administration
DFG	Dynamic form generator
Et_3_N	Triethylamine
DCM	Dichloromethane
CCK-8	Cell Counting Kit 8
MOE	Molecular Operating Environment
Glu	Glutamic acid
Asp	Aspartic acid
Cys	Cysteine
Phe	Phenylalanine
TLC	Thin-layer chromatography
