# Supplementary material for: Design, Synthesis, Molecular Docking, and Anticancer Activity of Chalcone Derivatives as VEGFR-2 Inhibitors
Source: Molecules. 2025 Nov 24;30(23):4526. doi: 10.3390/molecules30234526 (PMC12692968; doi:10.3390/molecules30234526)
Supplement: Supplementary file 1 [file molecules-30-04526-s001.zip › molecules-3958169-supplementary.pdf]

Supporting information

Design, synthesis, molecular docking, and anticancer activity of chalcone derivatives as VEGFR-2 inhibitors

Yu Mingjun[1*], Zhang Xin[2], Hui Zhu[1], Zhang Xiaoqian[1]

[1] Department of Traditional Chinese Medicine College, Bozhou University, Bozhou, Chian, 236800

[2] School of Chinese Medicine, Hong Kong Baptist University, Kowloon, Hong Kong SAR, China, 999077.

Corresponding authors

✉ Yu Mingjun

yuymj127@126.com

Contents:

1. Docking results of compounds 2a - 2s with VEGFR2: **Page no. 3-4**

2. Spectra of synthesized compounds: **Page no. 5-34**

Table S1 Docking results of compounds **2a - 2s** with VEGFR2

Comp.	ΔG (kcal/mol)	RMSD	Bonding interactions Residue/Distance (Å)
**2a**	-9.305	1.52	Glu885(2.85, 3.26), Asp1046(2.92), Leu840(4.23， 4.21)
**2b**	-9.486	1.13	Glu885(2.85, 3.18), Asp1046(2.92), Leu840(4.19, 4.22)
**2c**	-9.377	1.31	Glu885(2.77), Asp1046(2.94), Lys868(3.78)
**2d**	-9.432	1.42	Glu885(2.85, 3.24), Asp1046(2.92), Leu840(4.20, 4.22)
**2e**	-9.487	1.63	Glu885(2.85, 3.26), Asp1046(2.93), Leu840(433, 4.20, 4.25)
**2f**	-10.178	1.57	Glu885(2.85, 3.27), Asp1046(2.93), Leu840(4.23, 4.21)
**2g**	-9.493	1.53	Glu885(2.79), Asp1046(2.93), Leu840(4.19, 3.78)
**2h**	-9.635	1.66	Glu88(2.83, 3.24), Asp1046(2.94), Leu840(4.12)
**2i**	-9.476	1.07	Glu885(2.85), Asp1046(2.92), Lys868(3.80)
**2j**	-9.777	1.49	Glu885(2.83), Asp1046(2.96), Leu840(4.21)
**2k**	-9.784	1.37	Glu885(2.85, 3.25), Asp1046(2.93), Leu840(4.35, 4.18, 4.25)
**2l**	-9.504	1.42	Glu885(2.85, 3.25), Asp1046(2.93), Leu840(4.22, 4.21)
**2m**	-8.983	1.44	Glu885(2.58), Asp1046(2.98)
**2n**	-9.231	1.24	Glu885(2.57), Asp1046(2.96)
**2o**	-9.898	1.20	Glu885(2.64, 3.33), Asp1046(2.82), Leu840(4.10), Lys868(3.74), Cys919(2.70)
**2p**	-9.756	0.928	Glu885(2.83, 3.38), Asp1046(2.94), Leu840(4.48)
**2q**	-9.616	0.82	Glu885(2.67, 3.27), Asp1046(2.96), Leu840(4.26, 4,23)
**2r**	-9.767	1.99	Glu885(2.68, 3.26), Asp1046(2.95), Leu840(4.28,4.33)
**2s**	-9.436	1.35	Glu885(3.21), Asp1046(3.05), Phe1047(4.57),Cys919(3.57)
**sorafenib**	-10.230	0.22	Glu885(2.70,3.26), Asp1046(2.97), Cys919(3.22), Phe1047(3.65)

Note: Root mean square deviation (RMSD) values represents the root mean square deviation between the docked ligand and co-crystalline ligand.

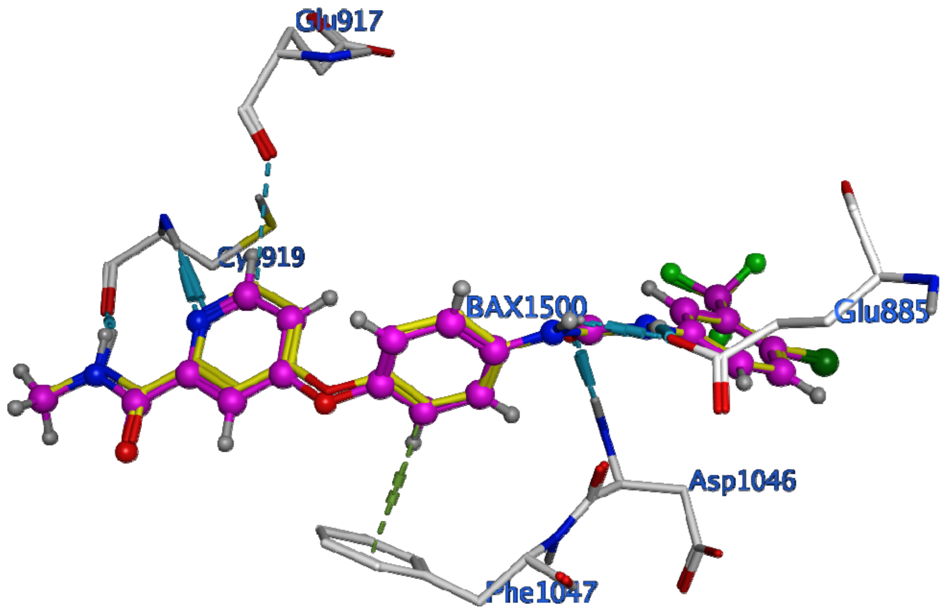

Yellow: Ligand of 4ASD　　　　Purple: **Sorafenib**

Fig S1 The overlapped structure of ligand of 4ASD and Sorafenib

2 Spectra of synthesized compounds:

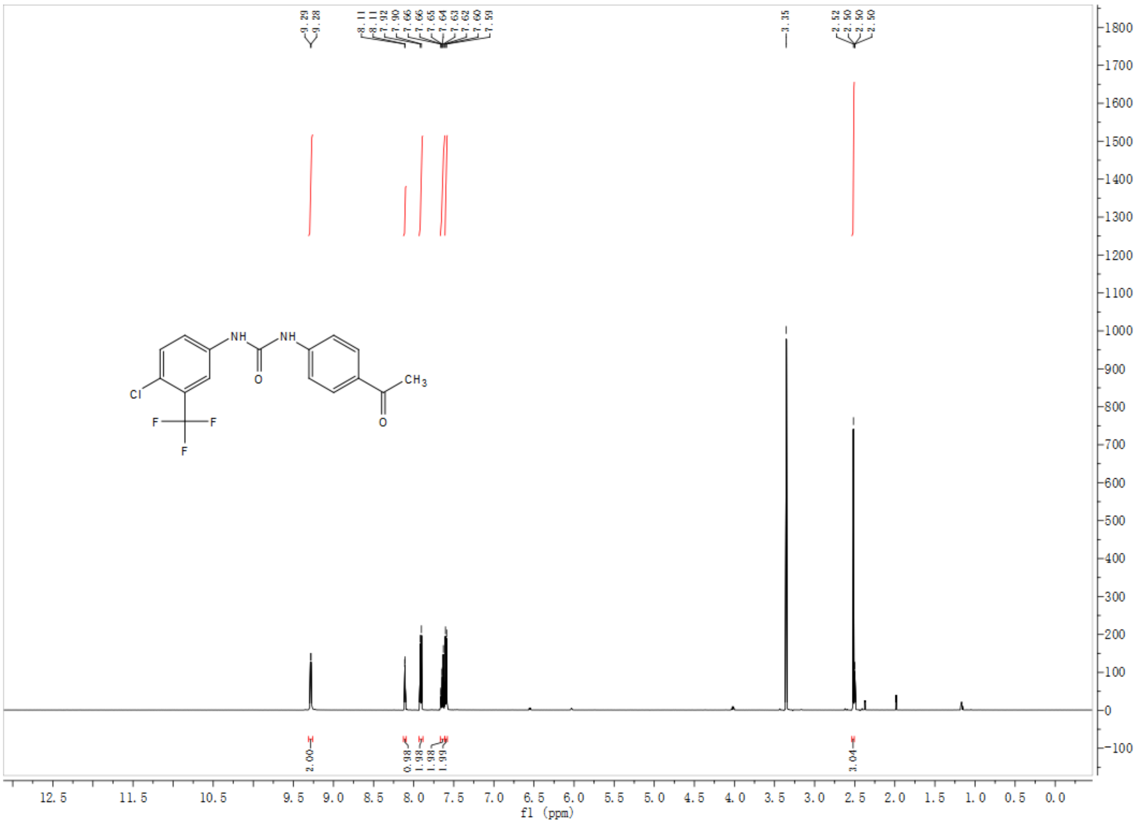

Fig S2 ^{1}H NMR (600 MHz, DMSO-d_6) spectra of **1a**

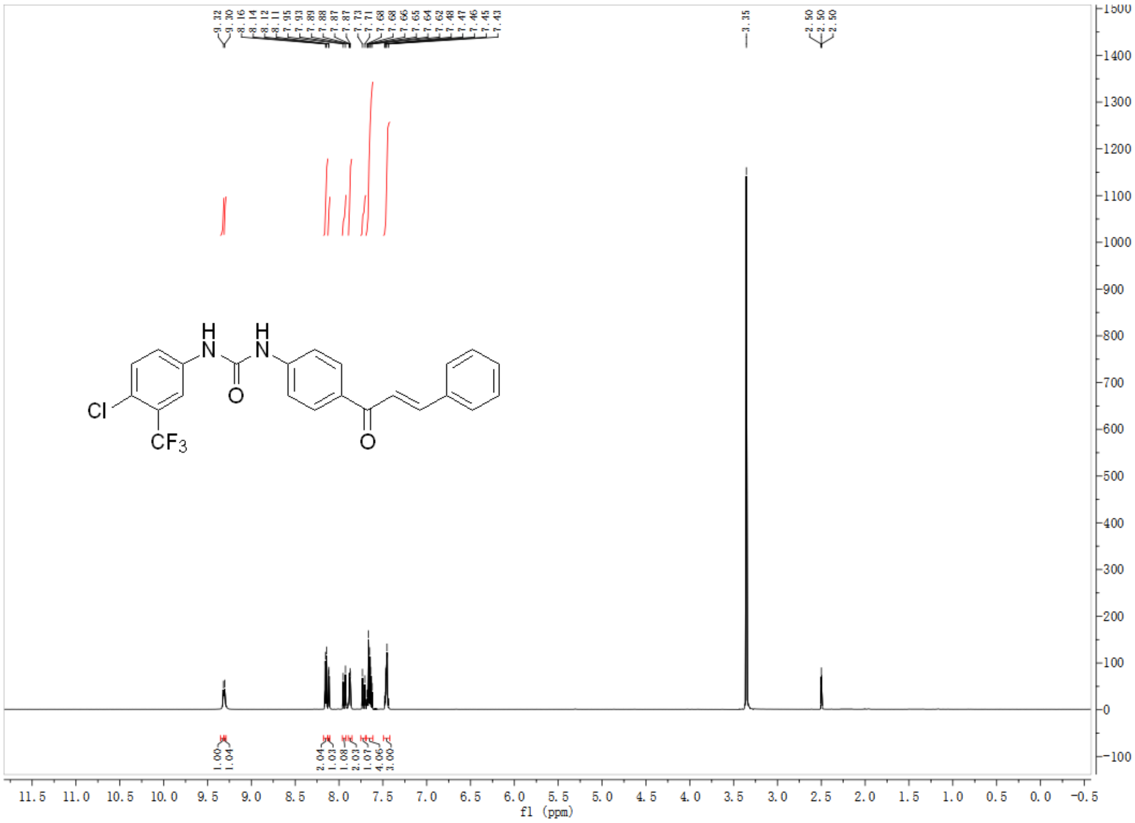

Fig S3 ^{1}H NMR (600 MHz, DMSO-d_6) spectra of **2a**

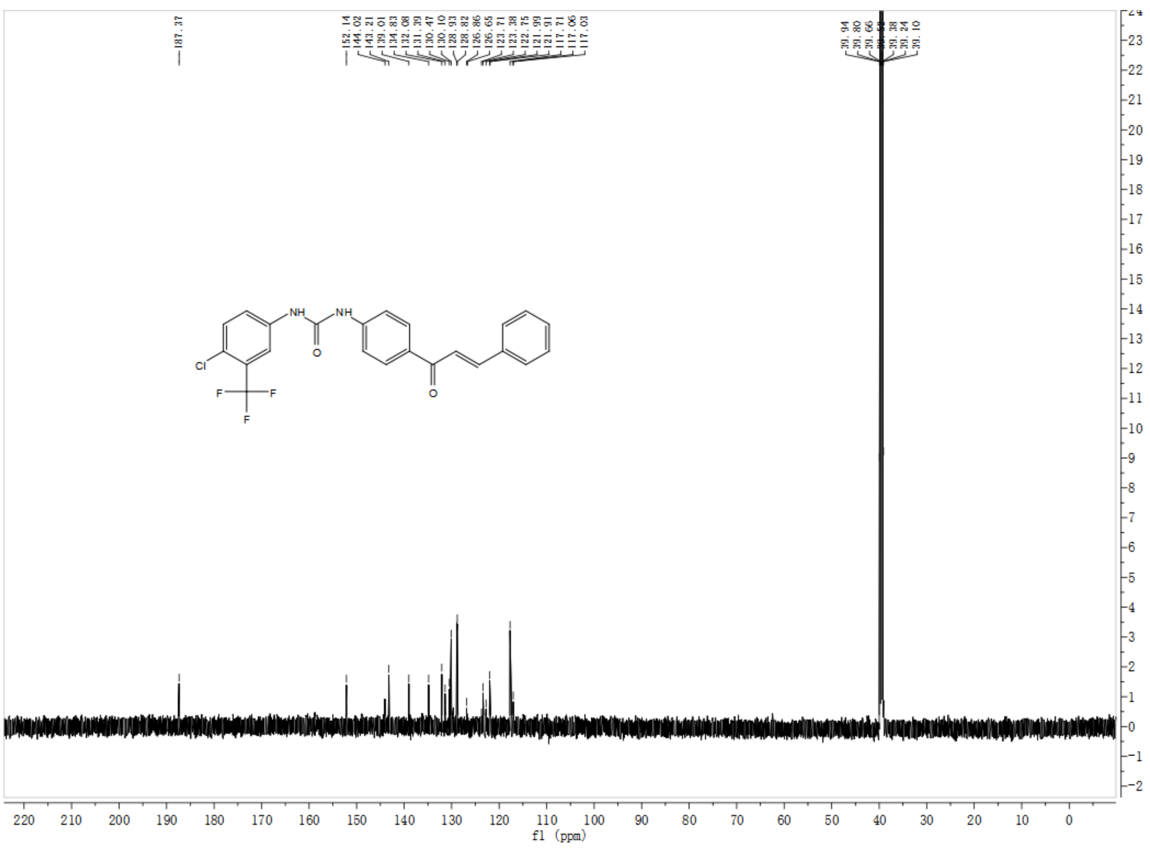

Fig S3 ^{13}C NMR (600 MHz, DMSO-d_6) spectra of **2a**

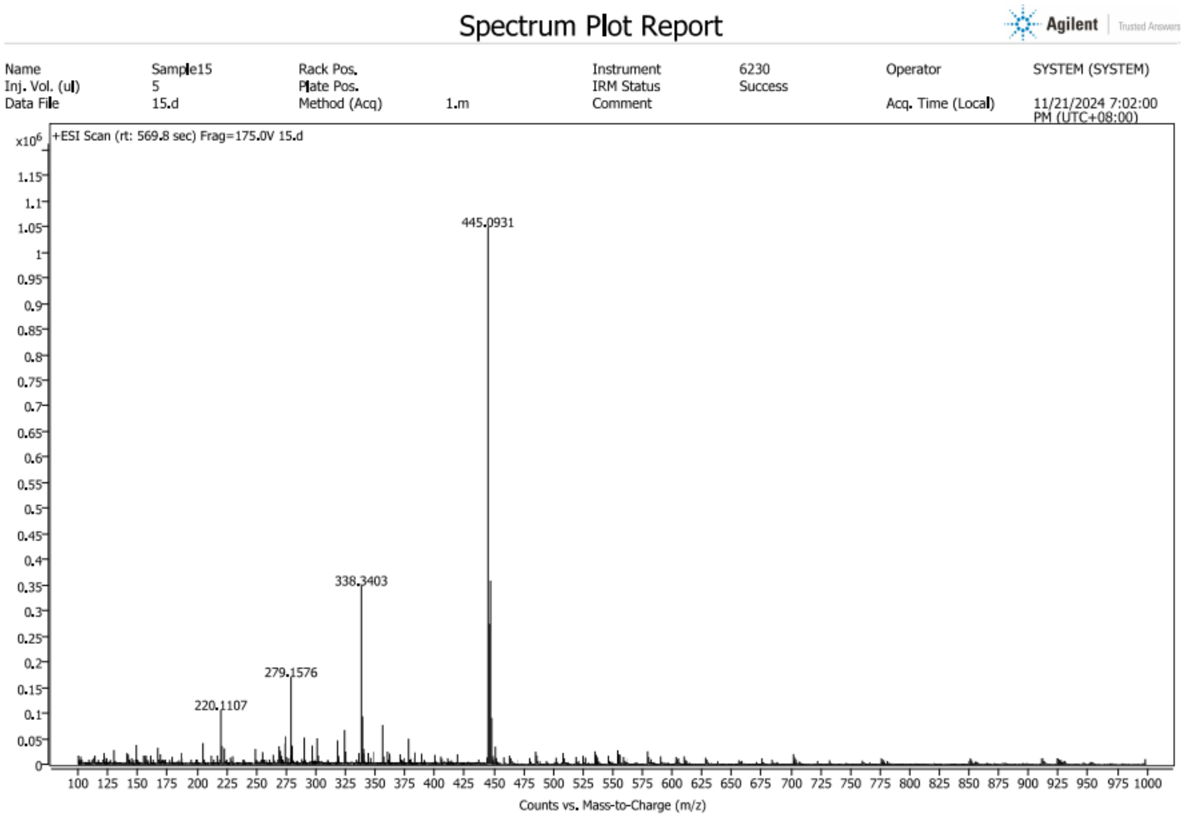

Fig S4 HR-MS spectrum of **2a**

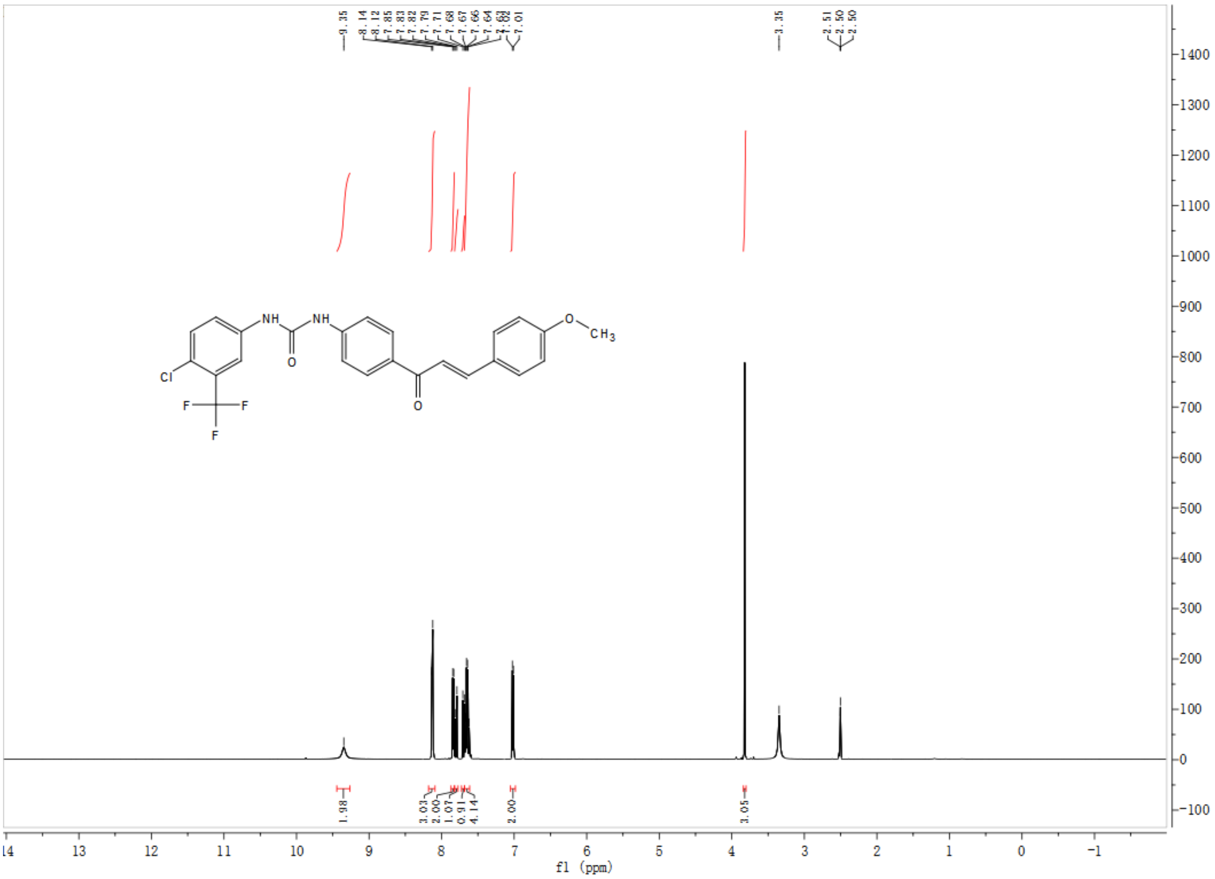

Fig S5 ^{1}H NMR (600 MHz, DMSO-d_6) spectra of **2b**

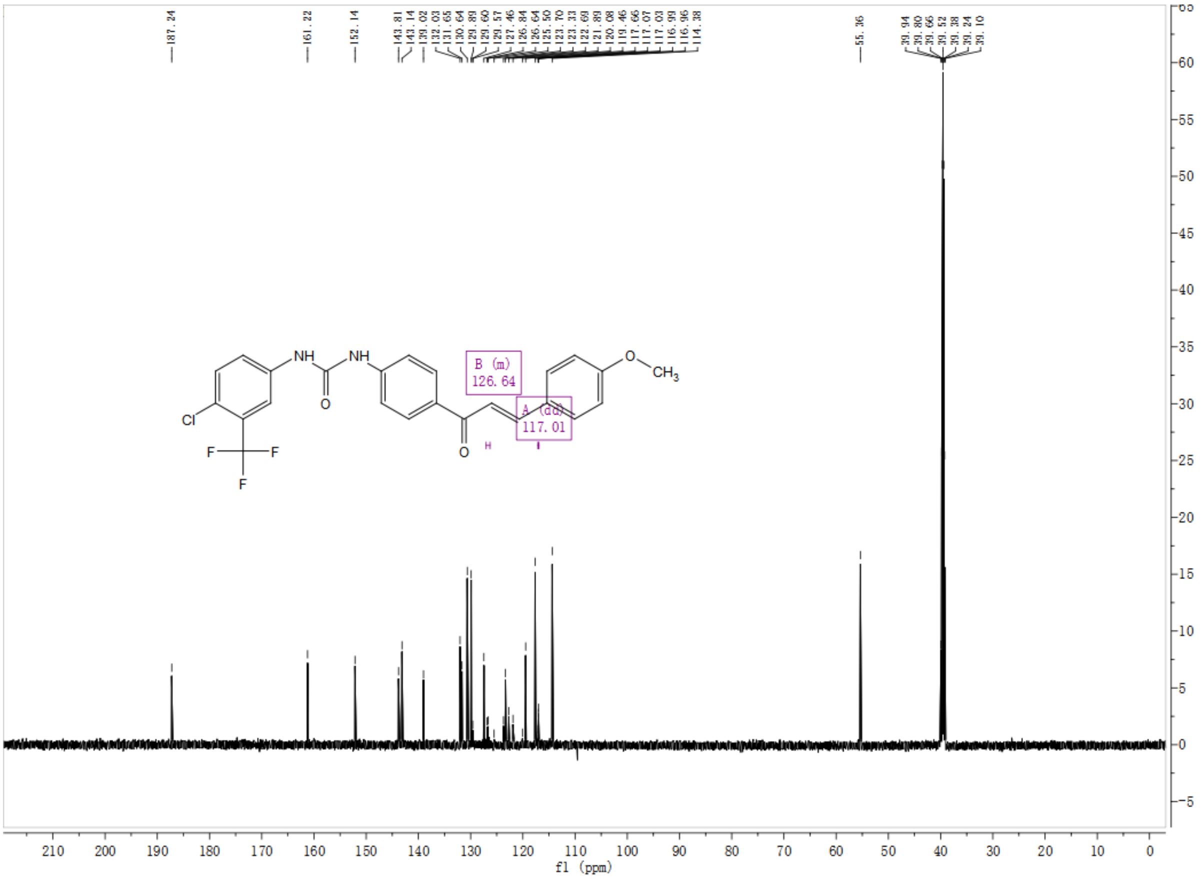

Fig S6 ^{13}C NMR (600 MHz, DMSO-d_6) spectra of **2b**

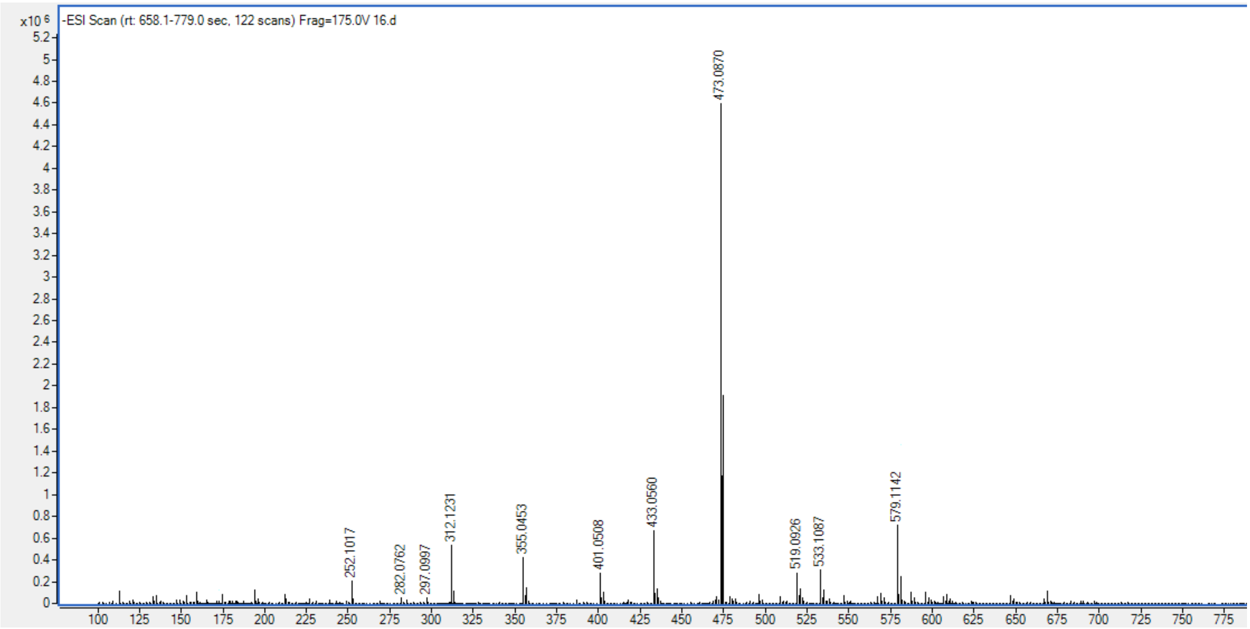

Fig S7 HR-MS spectrum of **2b**

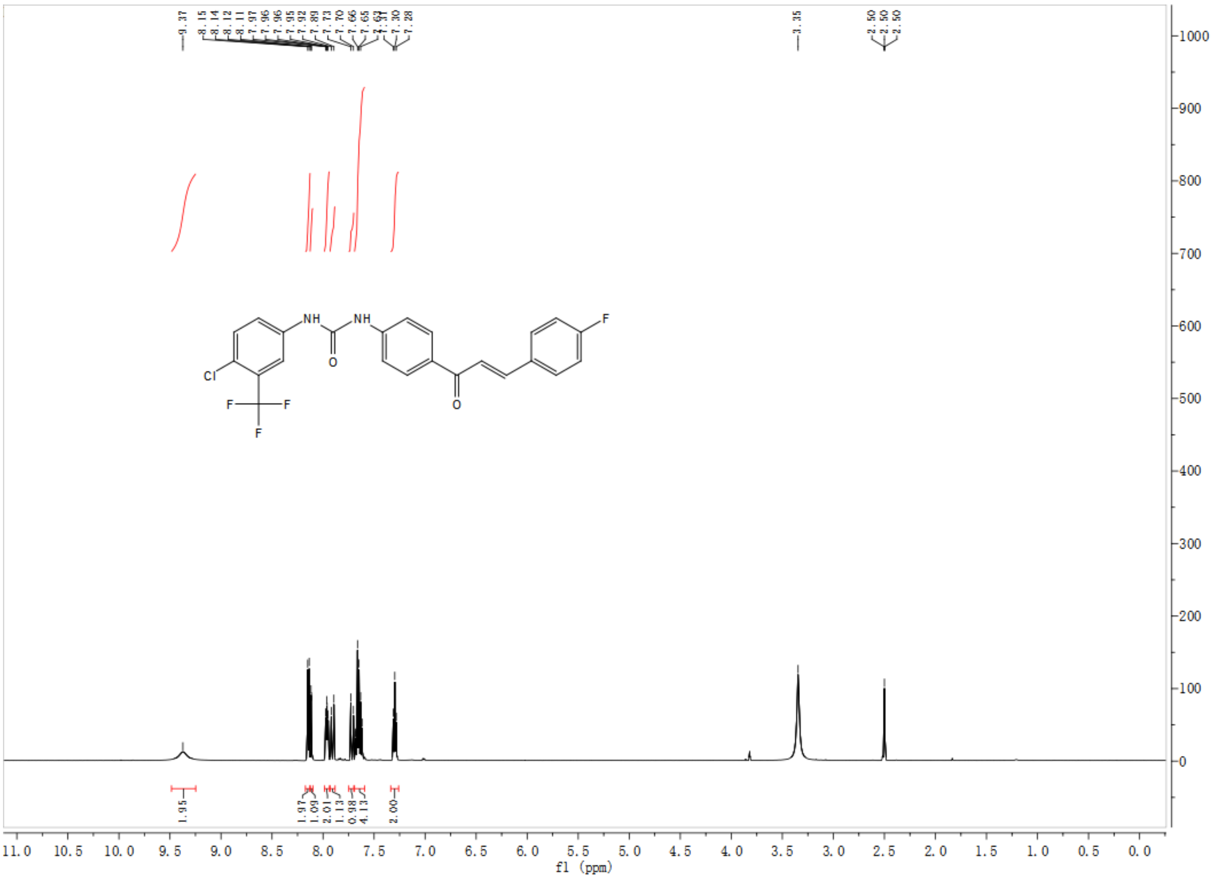

Fig S8 ^{1}H NMR (600 MHz, DMSO-d_6) spectra of **2c**

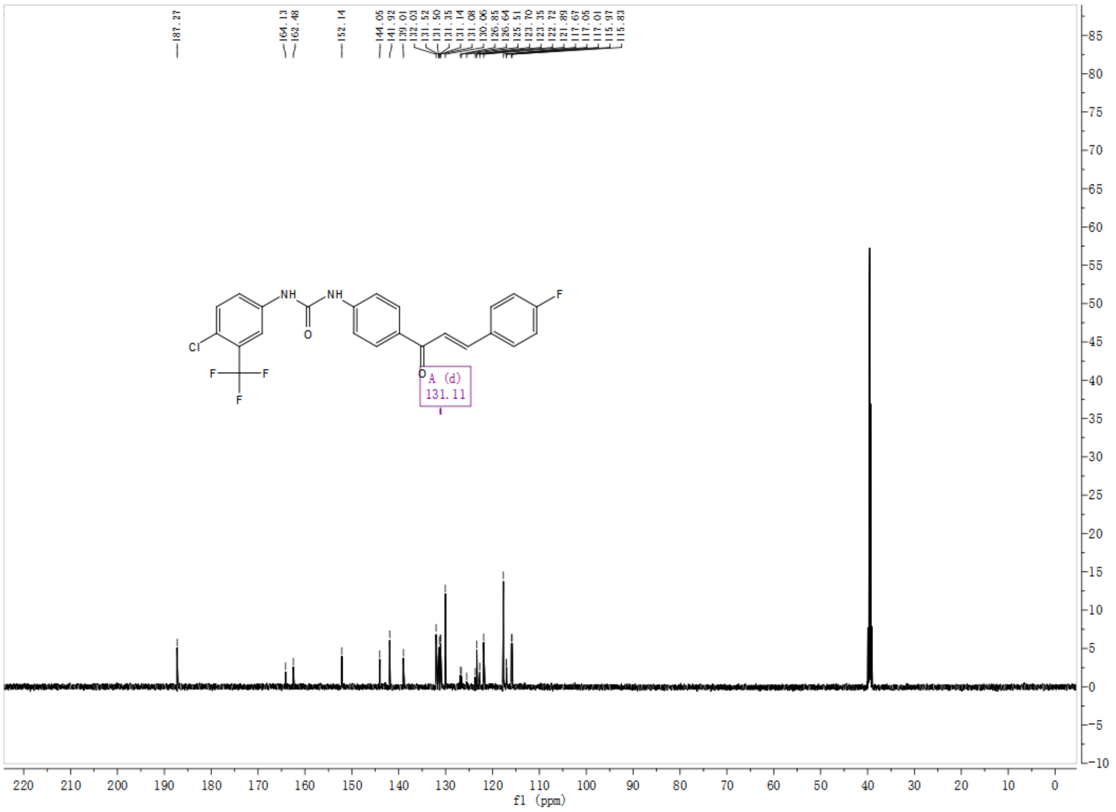

Fig S9 ^{13}C NMR (600 MHz, DMSO-d_6) spectra of **2c**

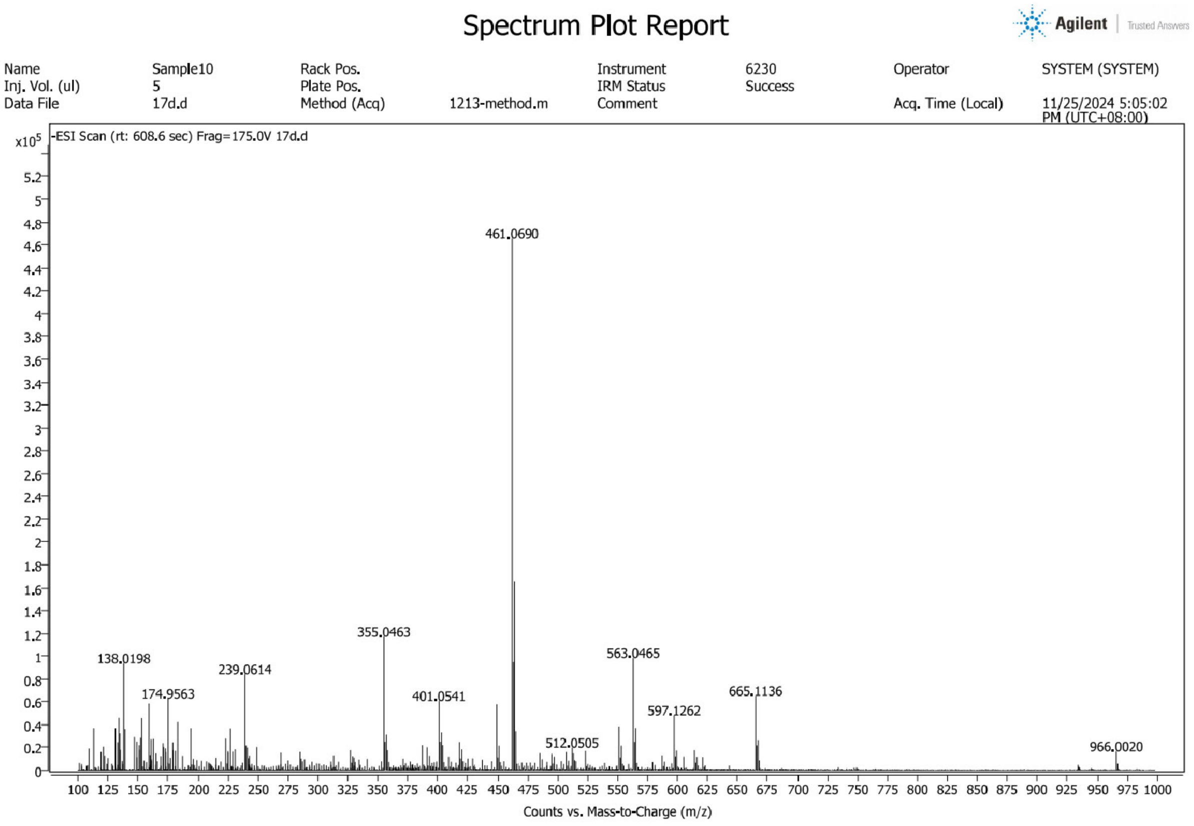

Fig S10 HR-MS spectrum of **2c**

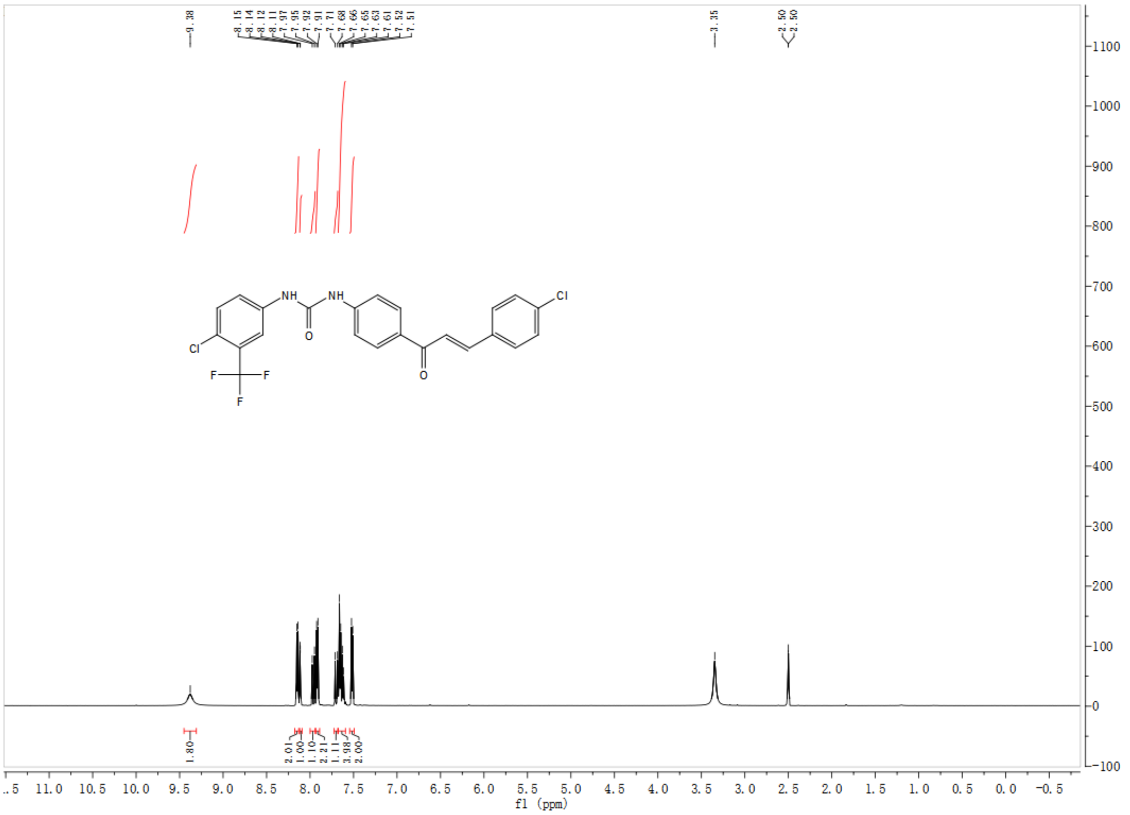

Fig S11 ^{1}H NMR (600 MHz, DMSO-d_6) spectra of **2d**

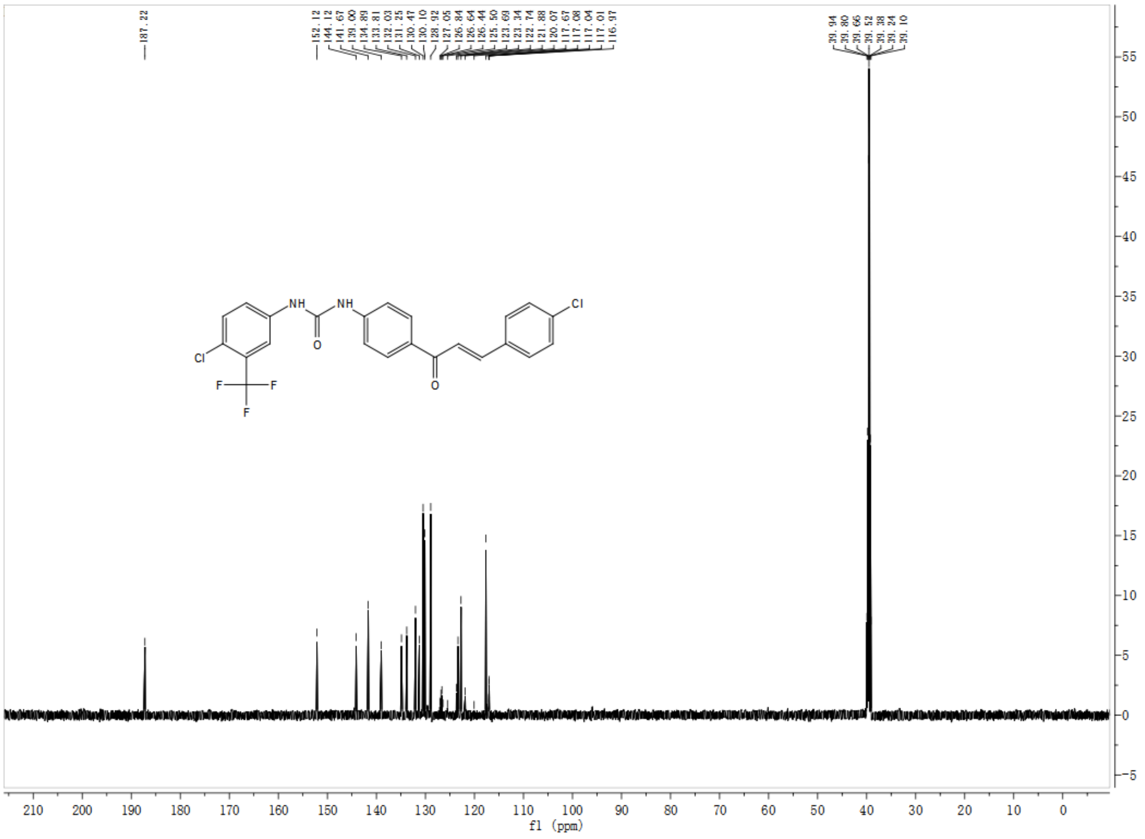

Fig S12 ^{13}C NMR (600 MHz, DMSO-d_6) spectra of **2d**

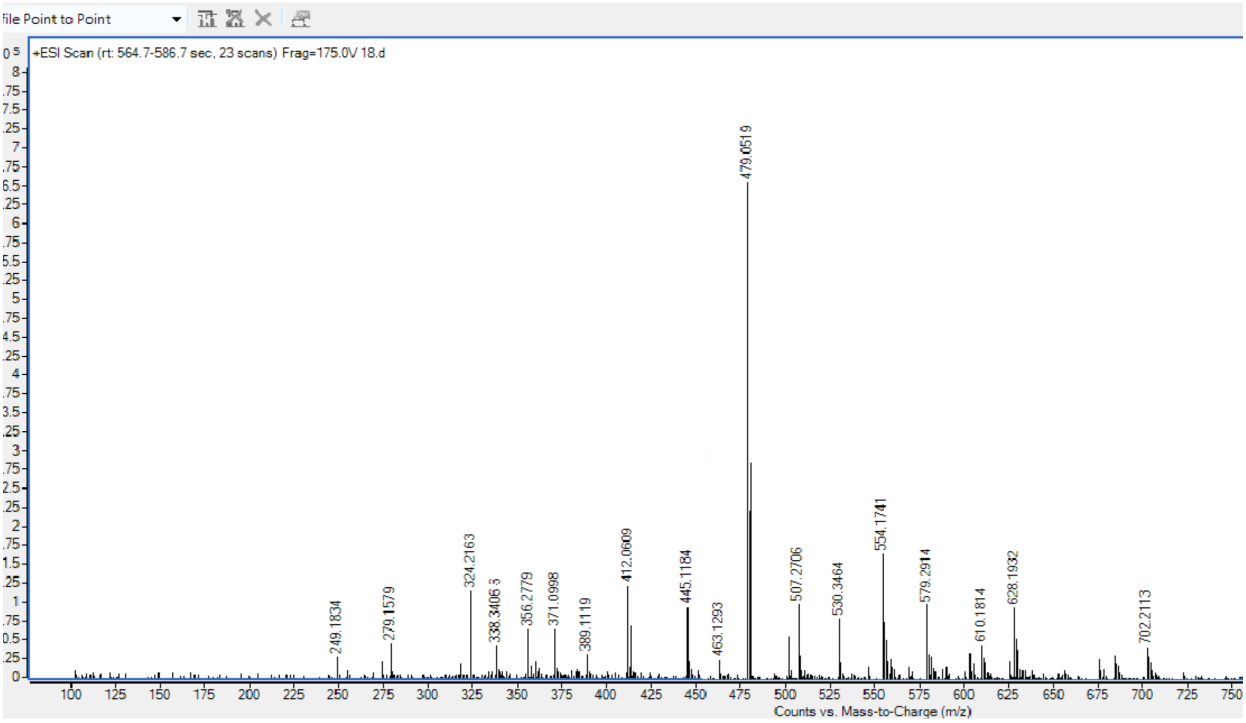

Fig S13 HR-MS spectrum of **2d**

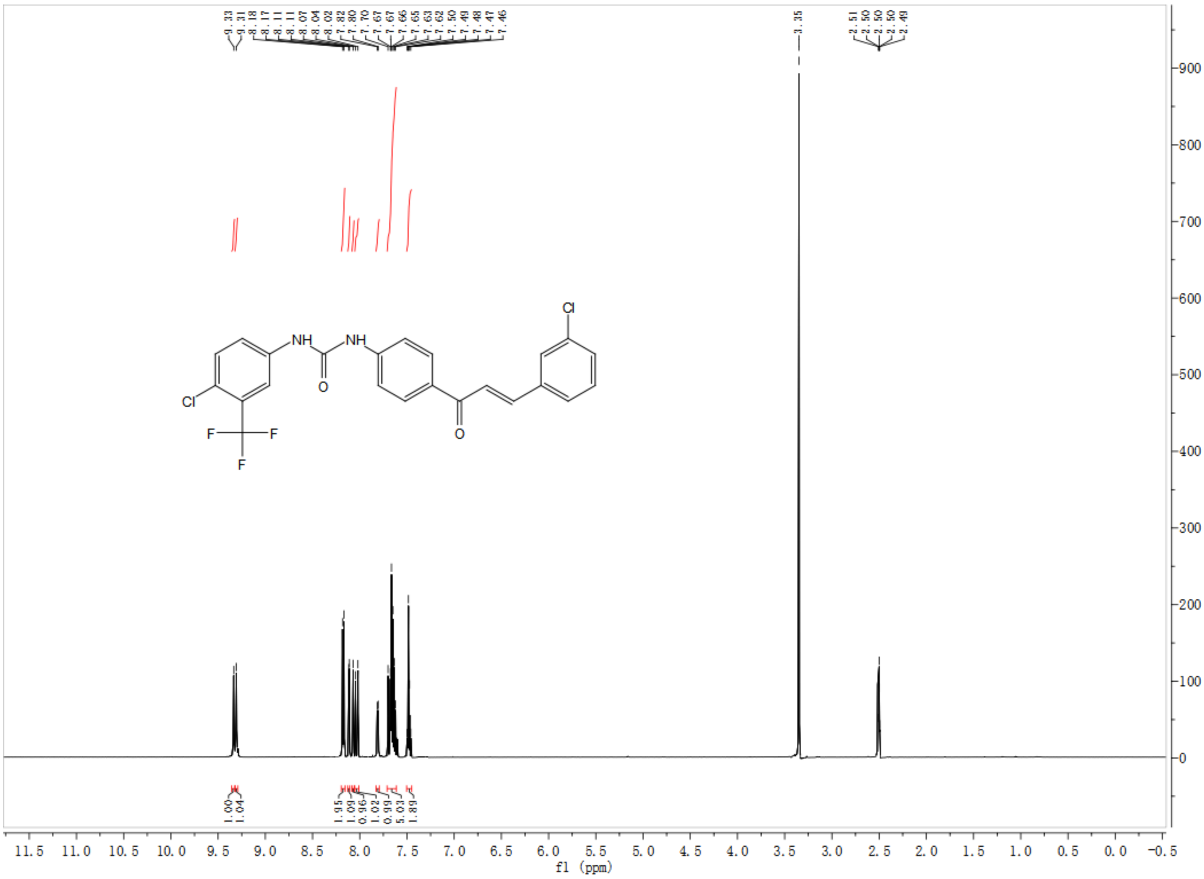

Fig S14 ^{1}H NMR (600 MHz, DMSO-d_6) spectra of **2e**

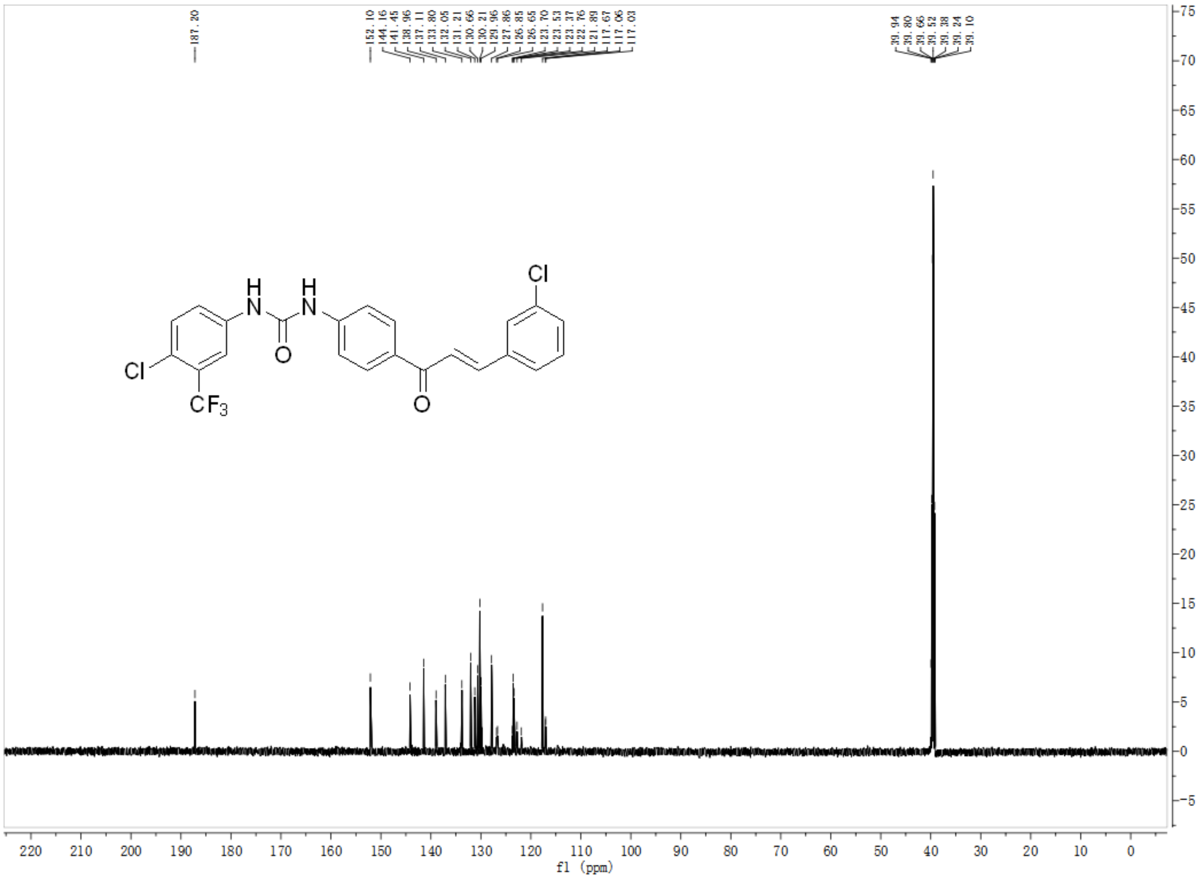

Fig S15 ^{13}C NMR (600 MHz, DMSO-d_6) spectra of **2e**

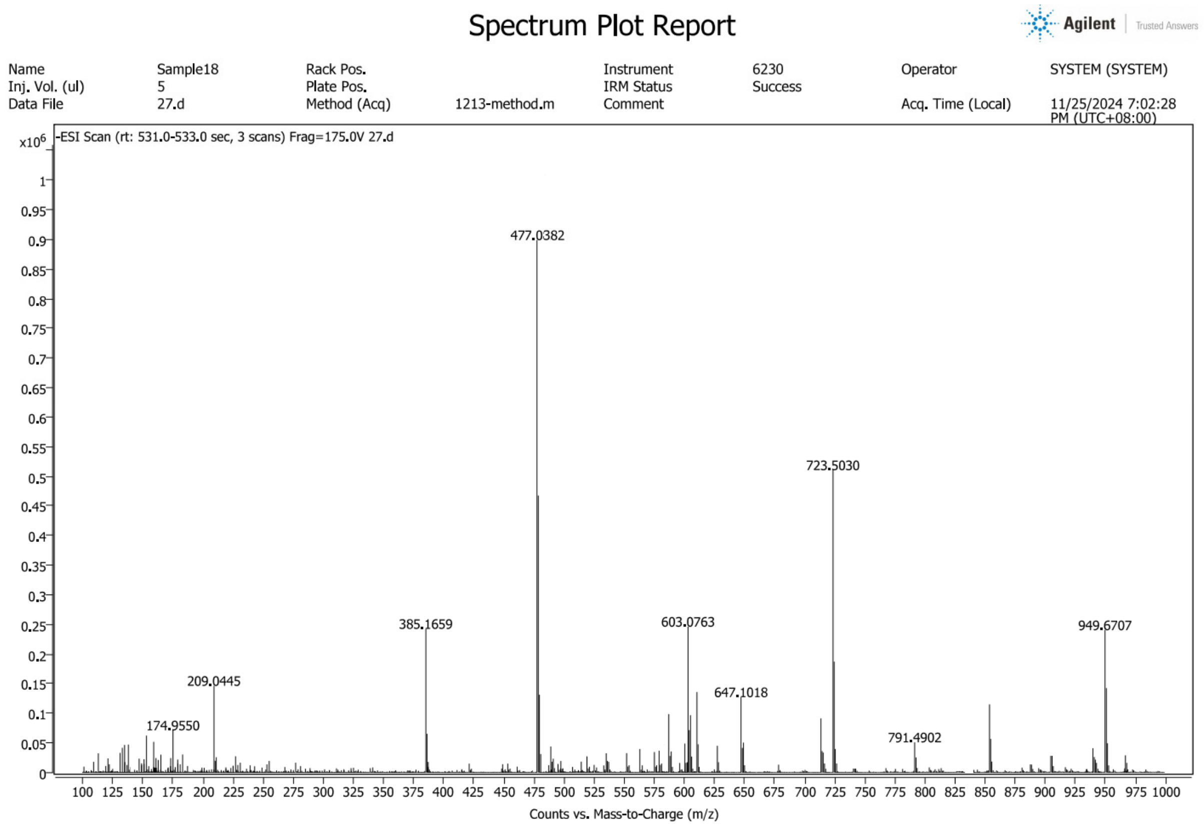

Fig S16 HR-MS spectrum of **2e**

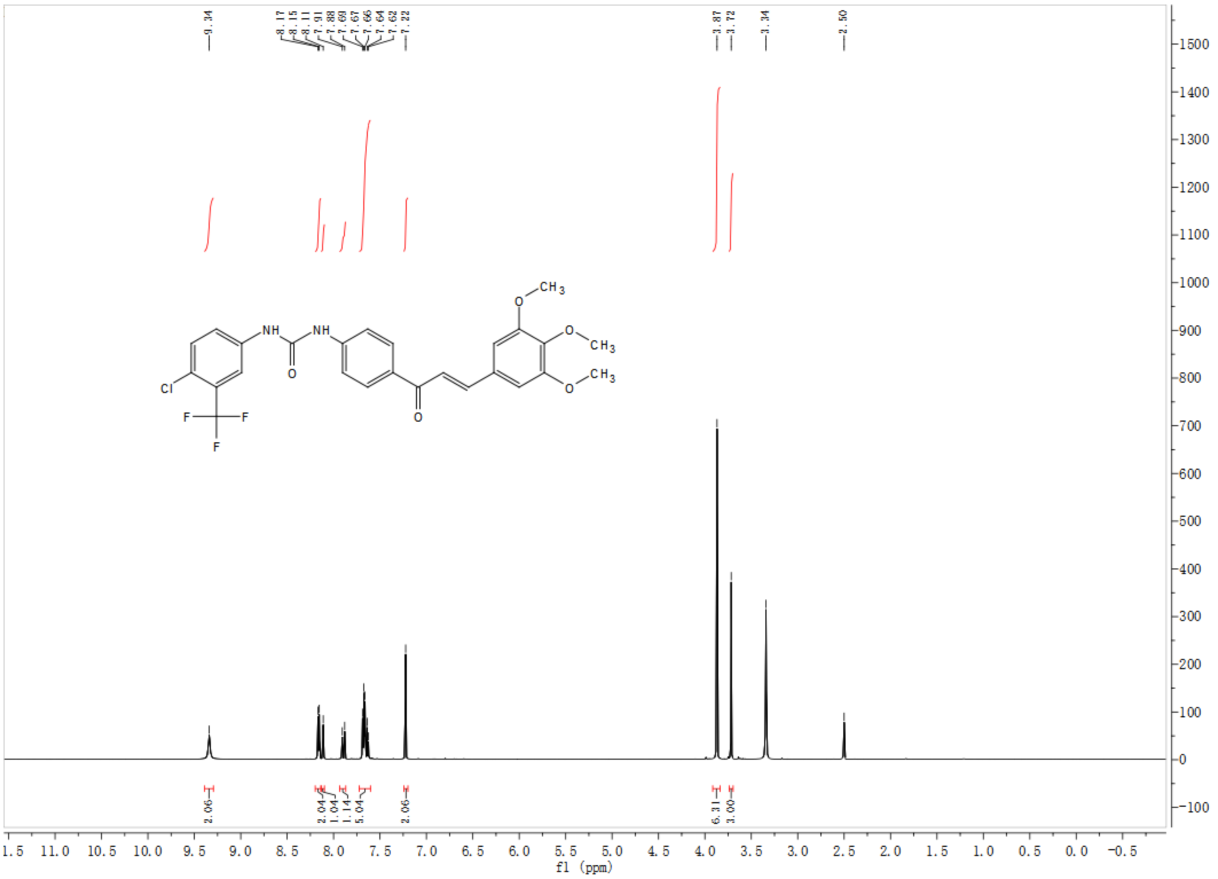

Fig S17 ^{1}H NMR (600 MHz, DMSO-*d*6) spectra of **2f**

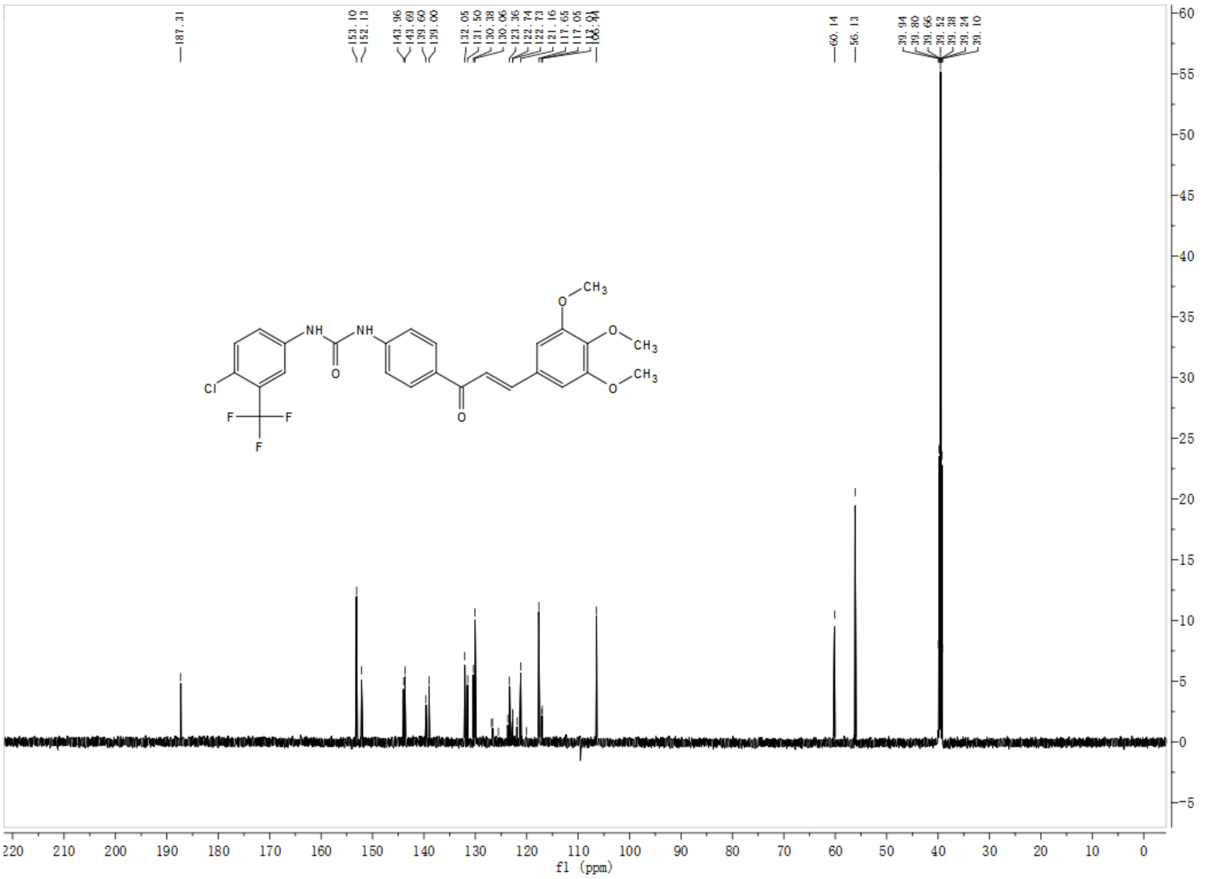

Fig S18 ^{13}C NMR (600 MHz, DMSO-*d*6) spectra of **2f**

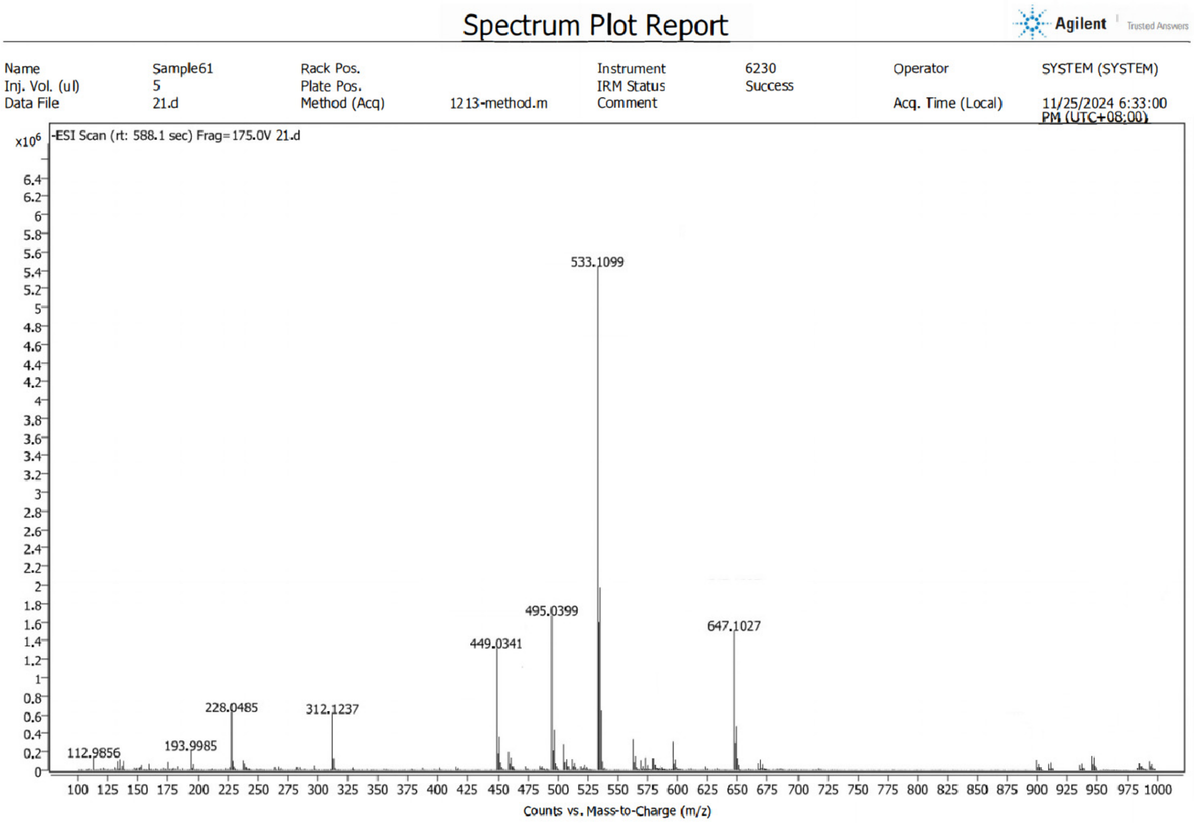

Fig S19 HR-MS spectrum of **2f**

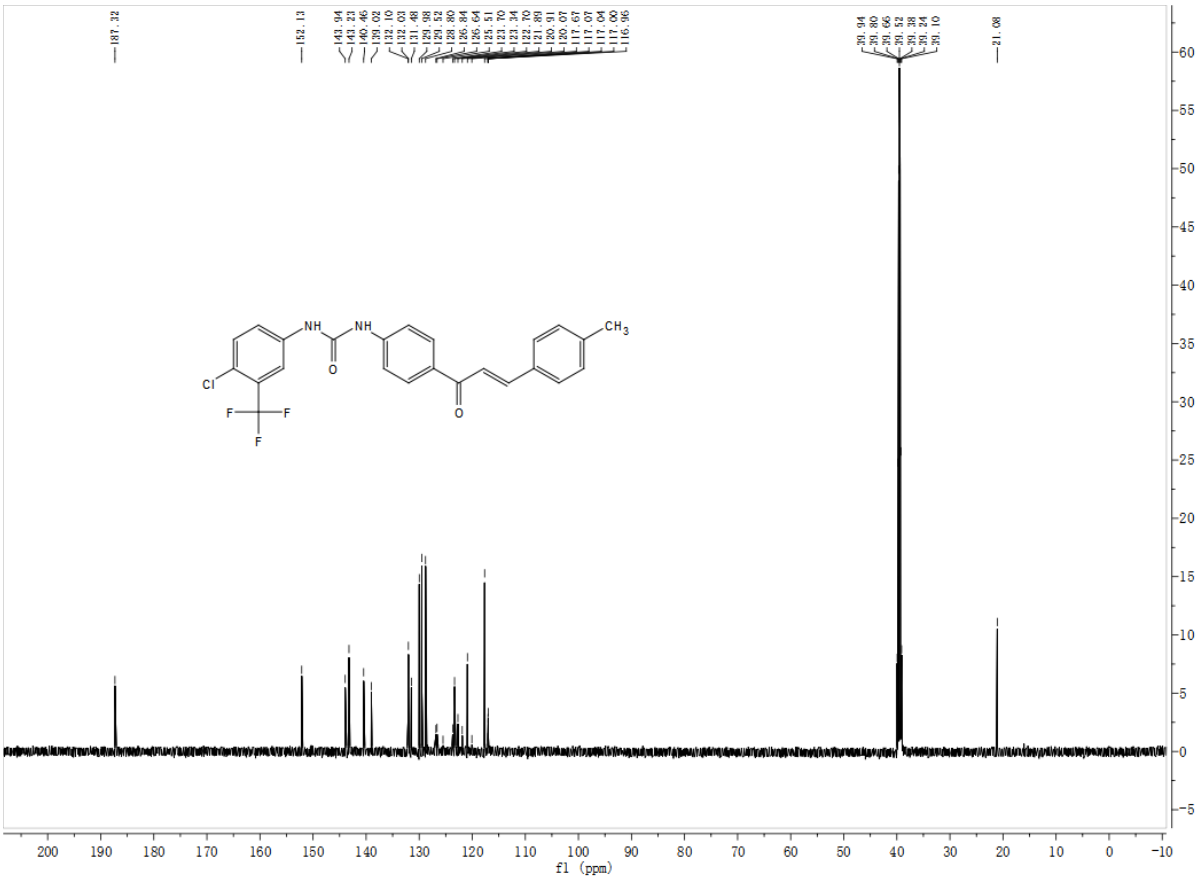

Fig S20 ^{1}H NMR (600 MHz, DMSO-d_6) spectra of **2g**

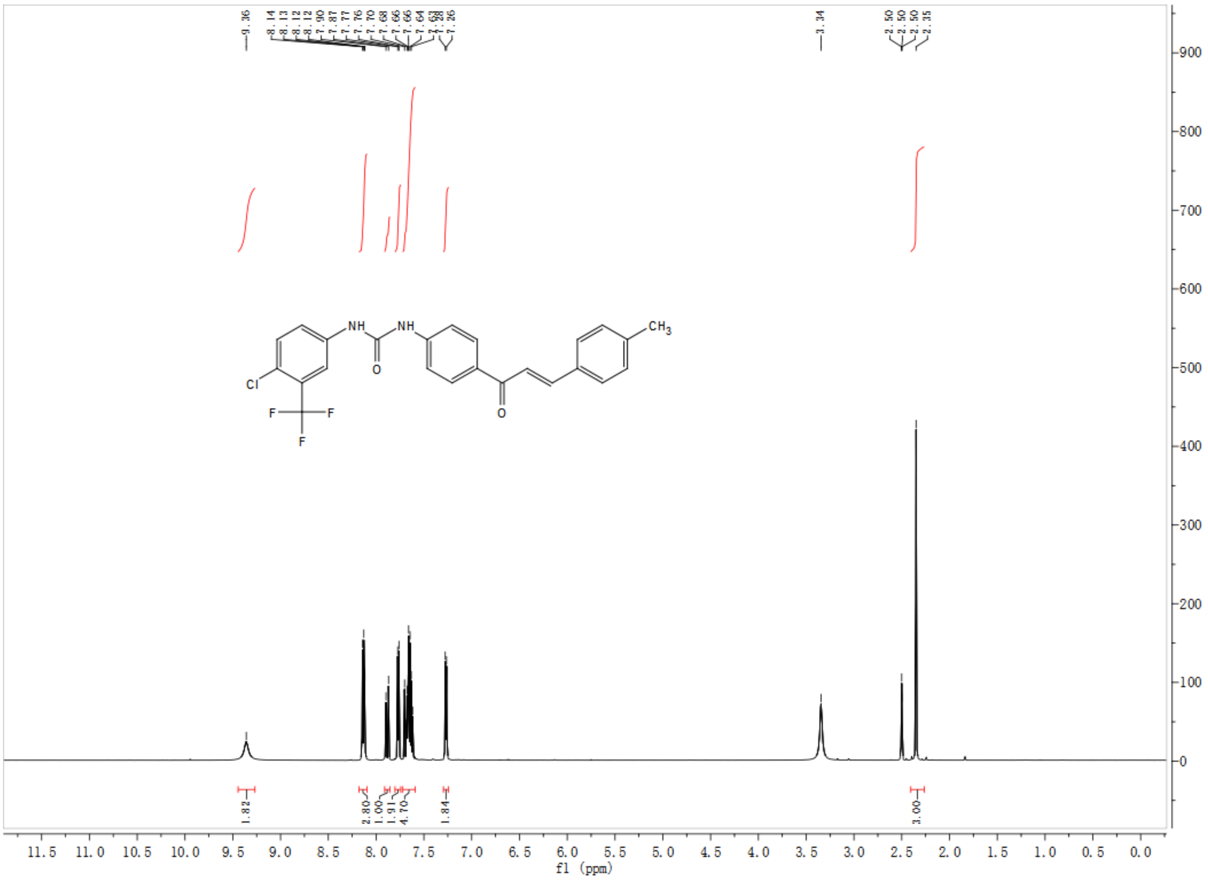

Fig S21 ^{13}C NMR (600 MHz, DMSO-d_6) spectra of **2g**

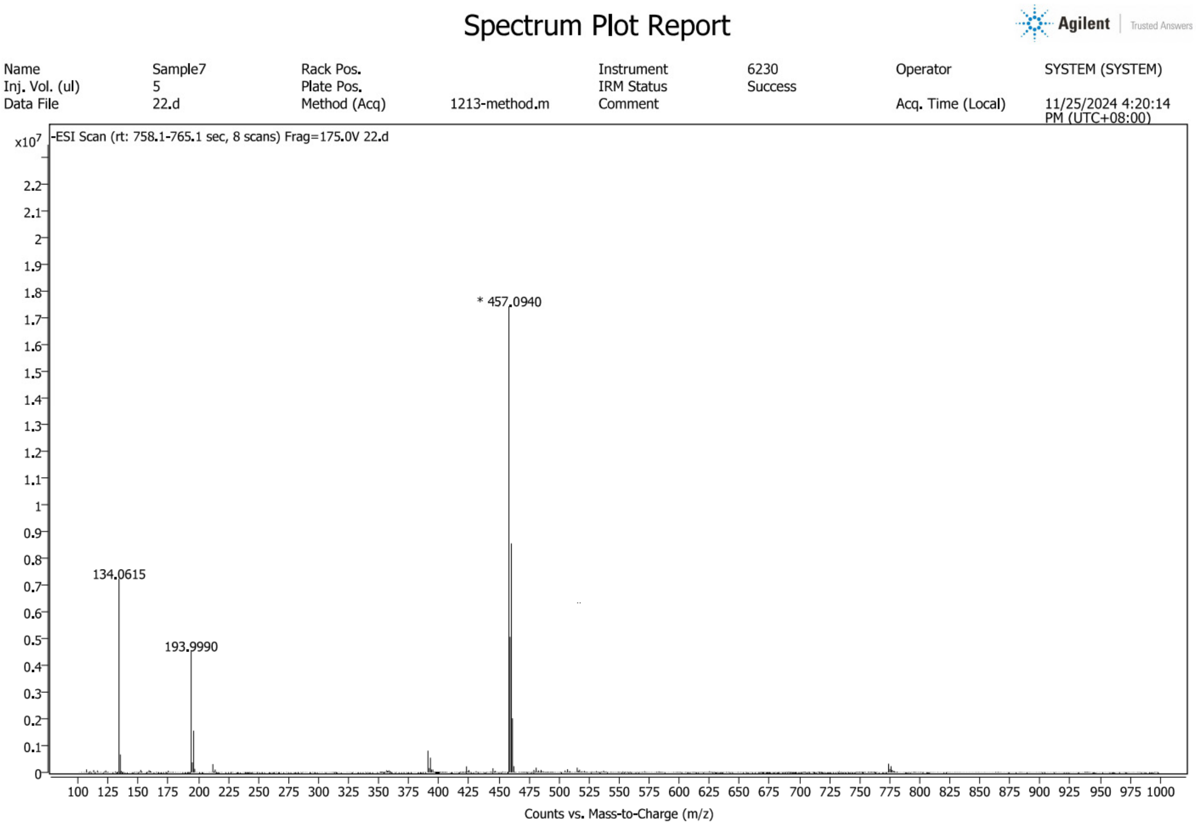

Fig S22 HR-MS spectrum of **2g**

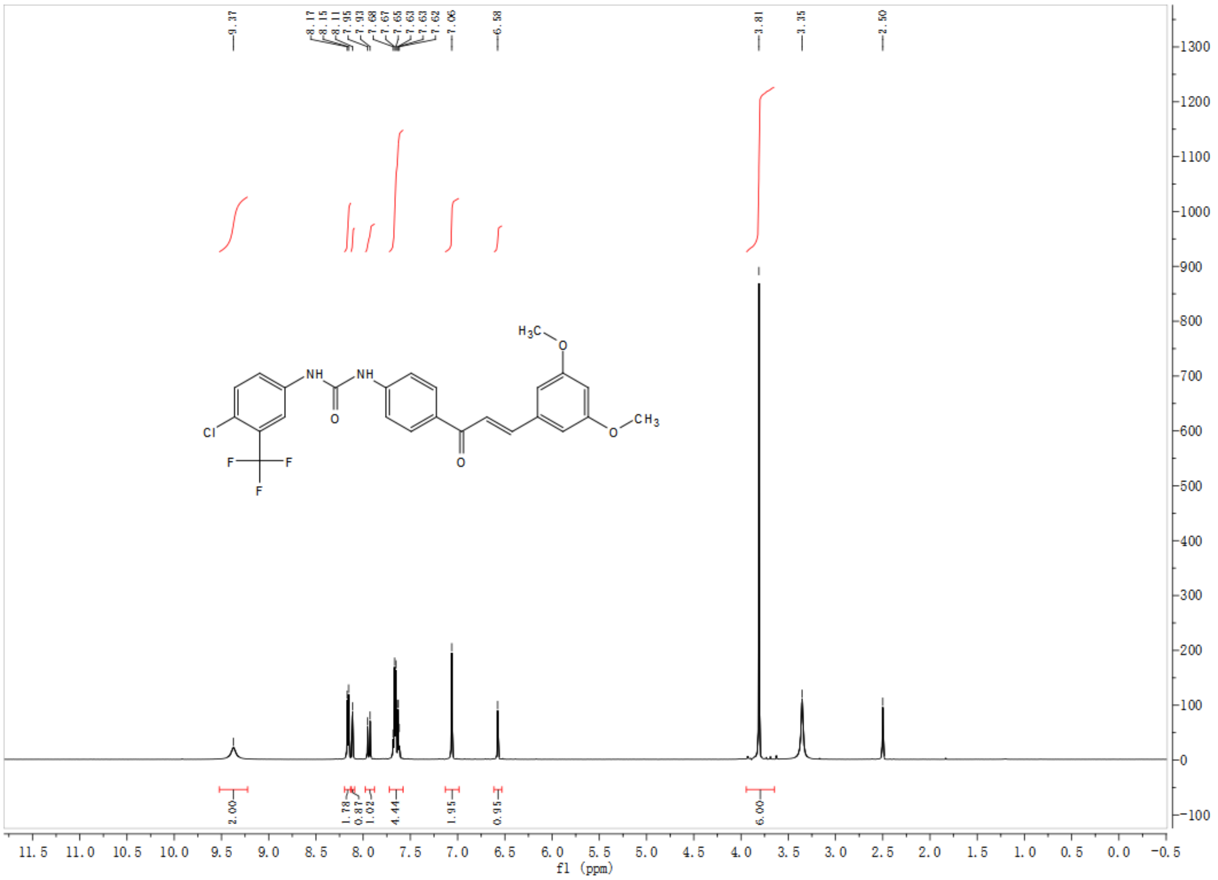

Fig S23 ^{1}H NMR (600 MHz, DMSO-d_6) spectra of **2h**

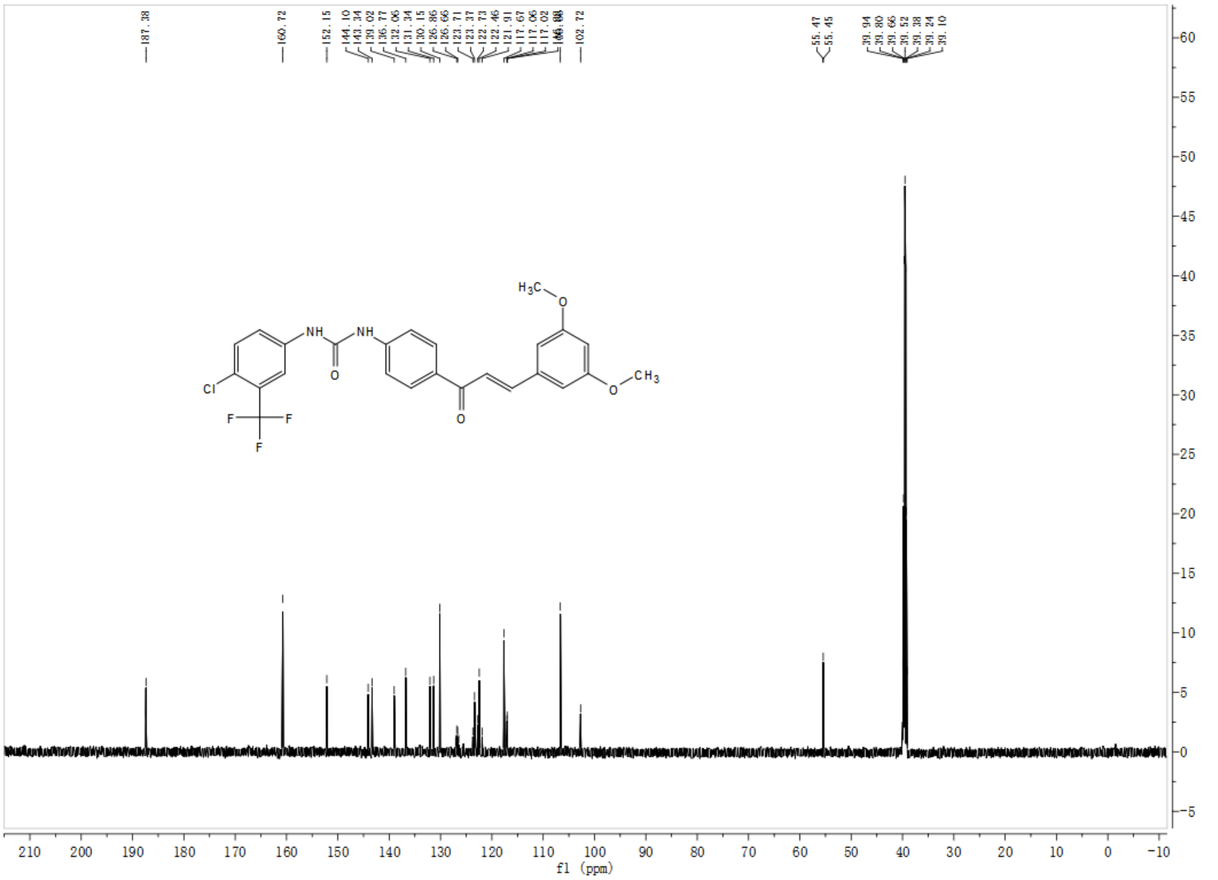

Fig S24 ^{13}C NMR (600 MHz, DMSO-d_6) spectra of **2h**

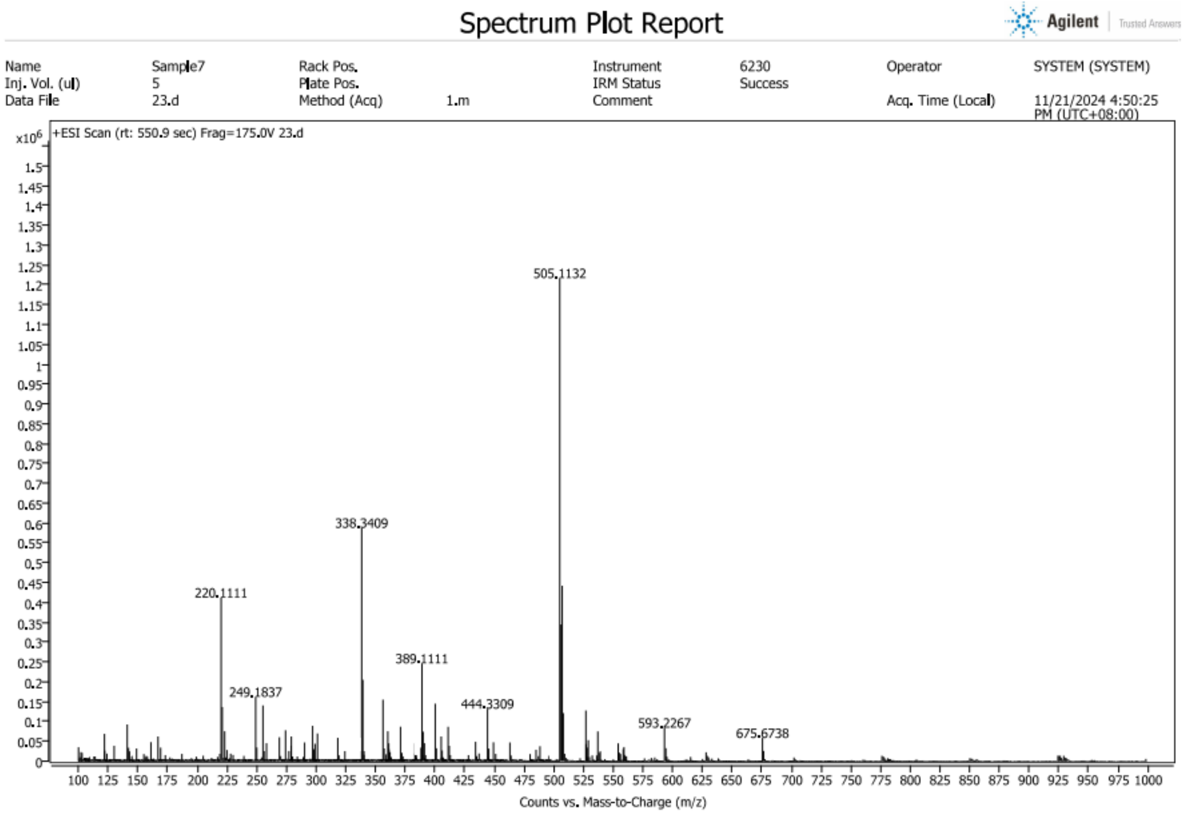

Fig S25 HR-MS spectrum of **2h**

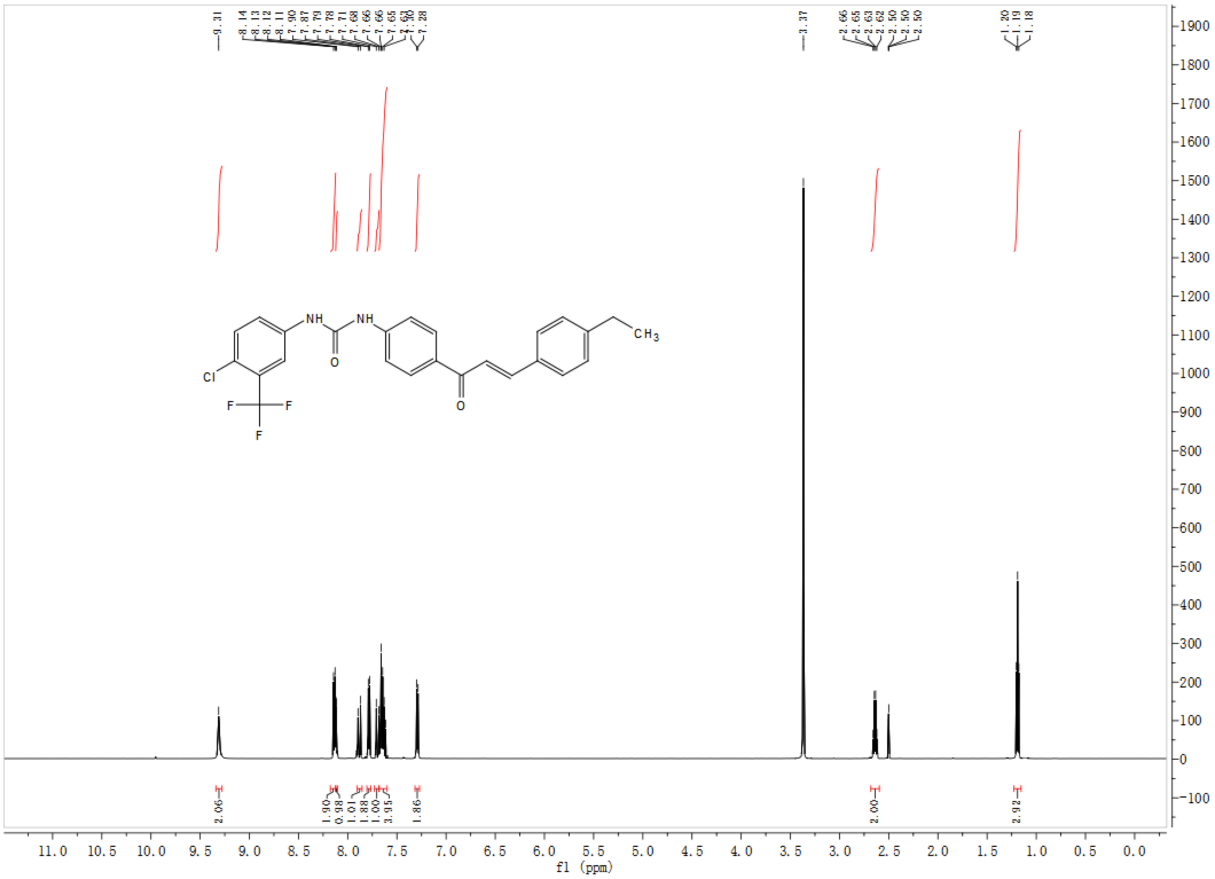

Fig S26 ^{1}H NMR (600 MHz, DMSO-d_6) spectra of **2i**

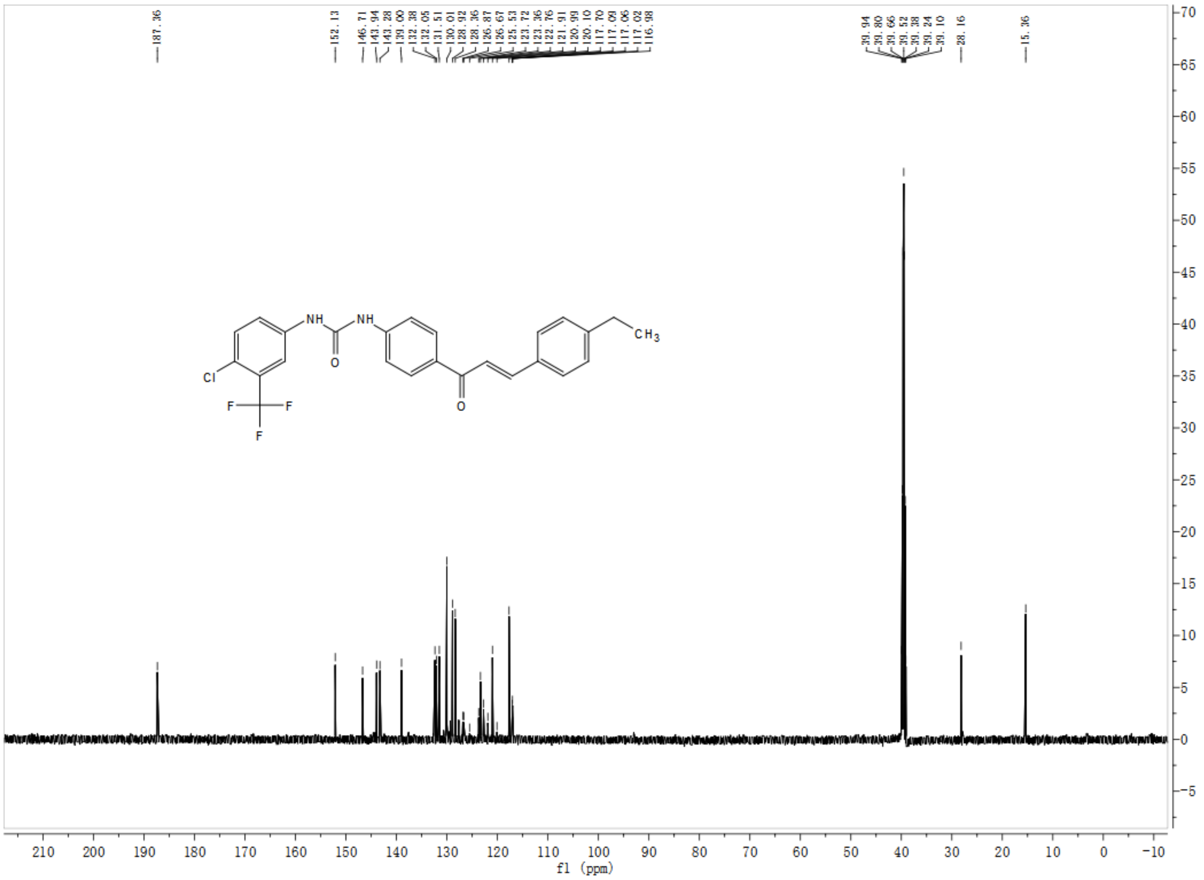

Fig S27 ^{13}C NMR (600 MHz, DMSO-d_6) spectra of **2i**

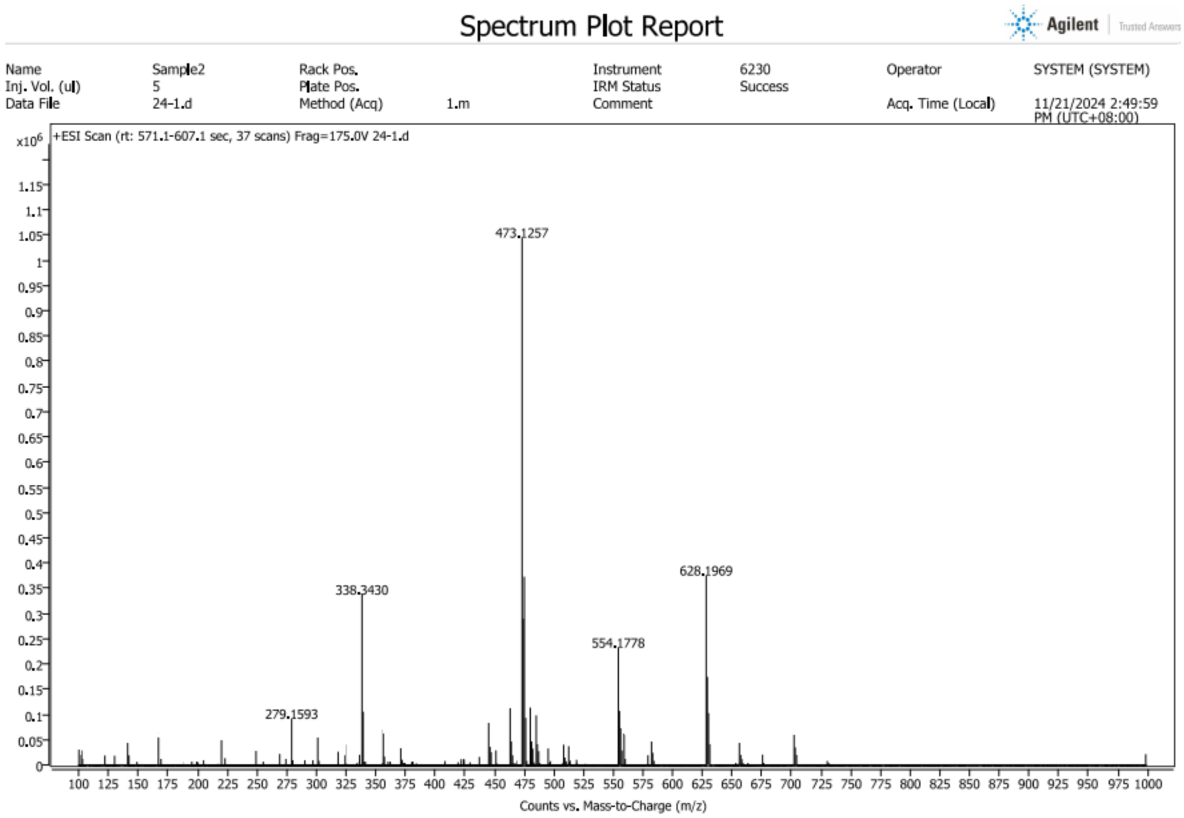

Fig S28 HR-MS spectrum of **2i**

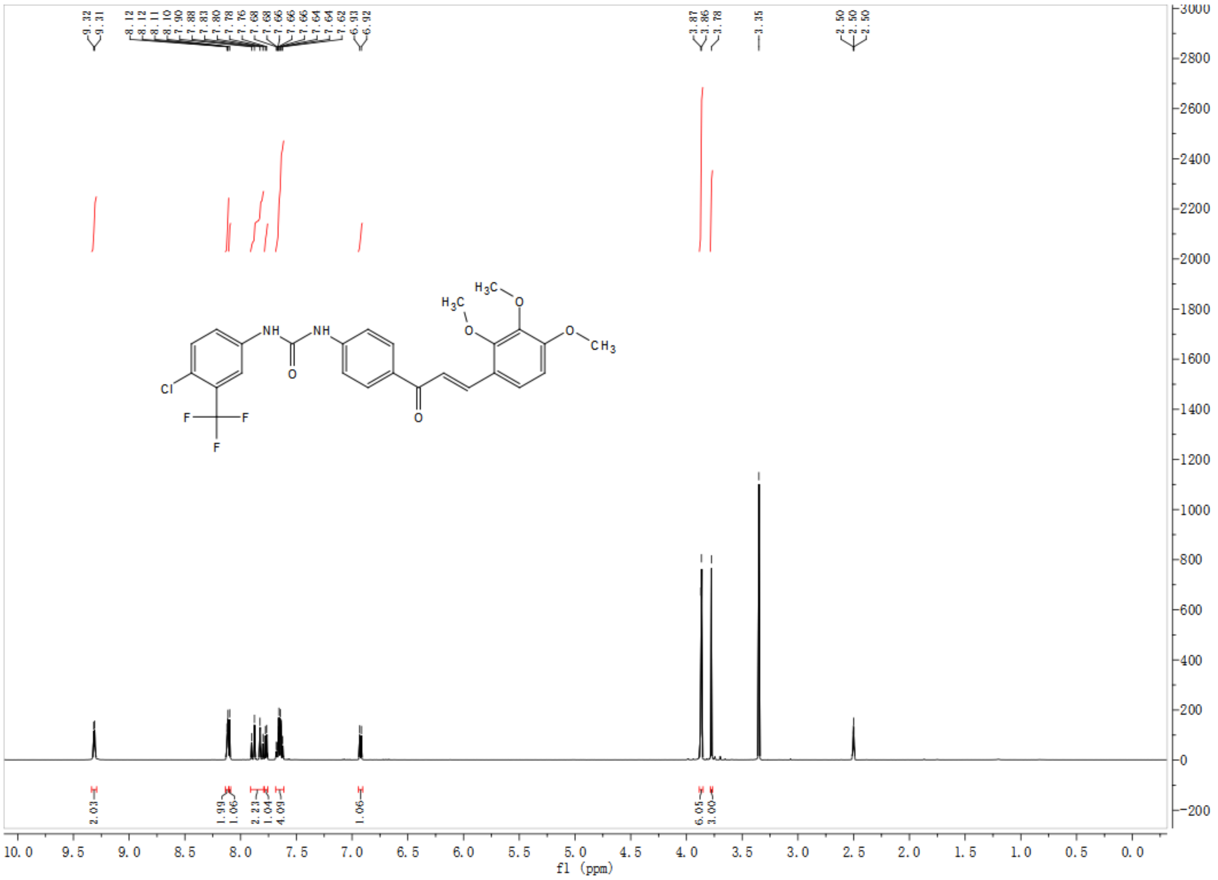

Fig S29 ^{1}H NMR (600 MHz, DMSO-*d*$_6$) spectra of **2j**

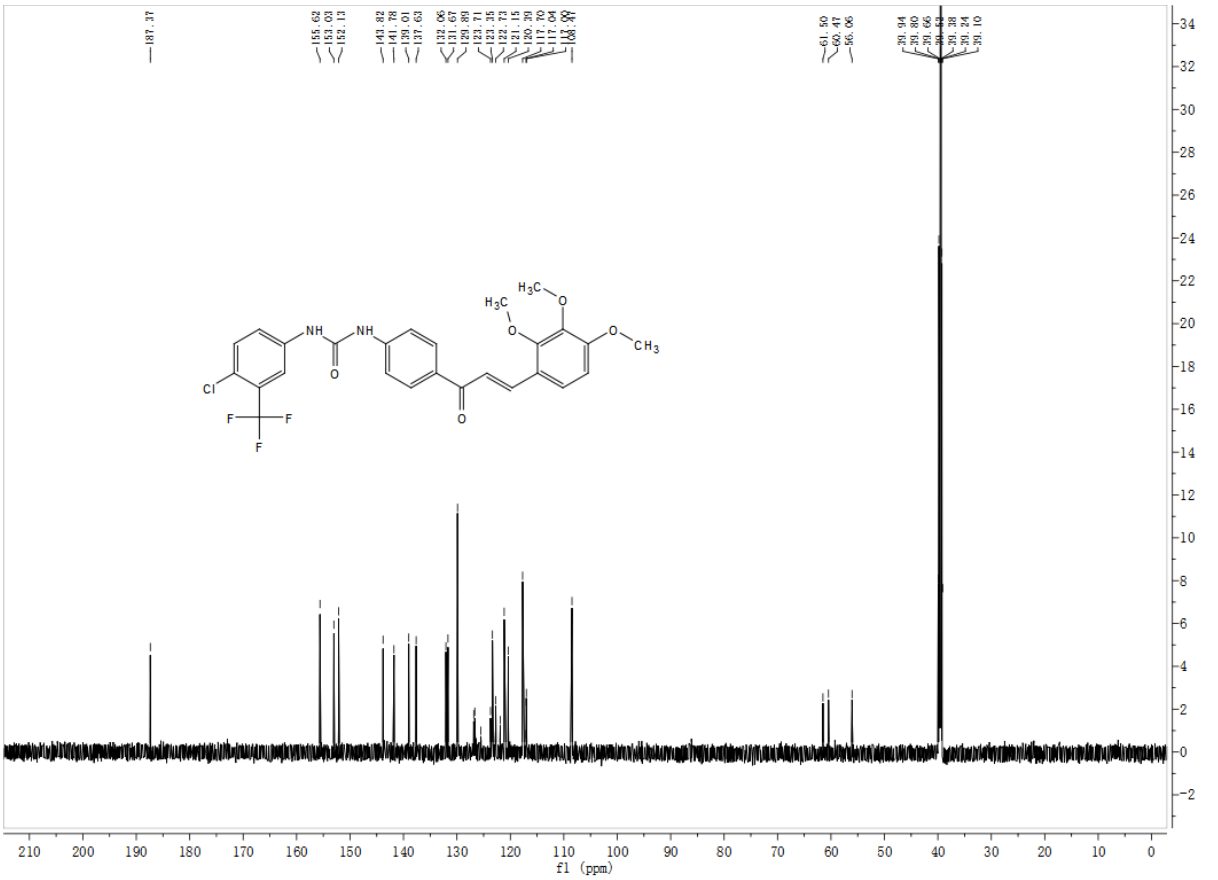

Fig S30 ^{13}C NMR (600 MHz, DMSO-*d*$_6$) spectra of **2j**

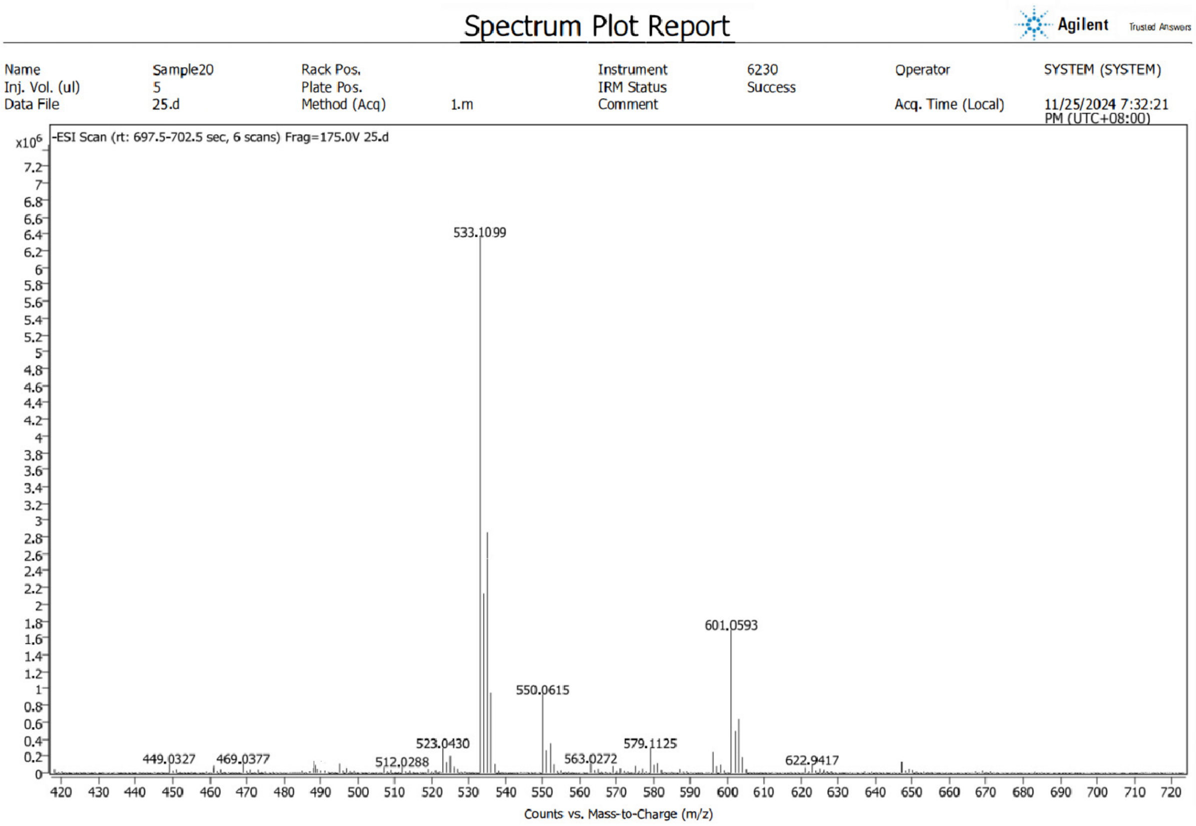

Fig S31 HR-MS spectrum of **2j**

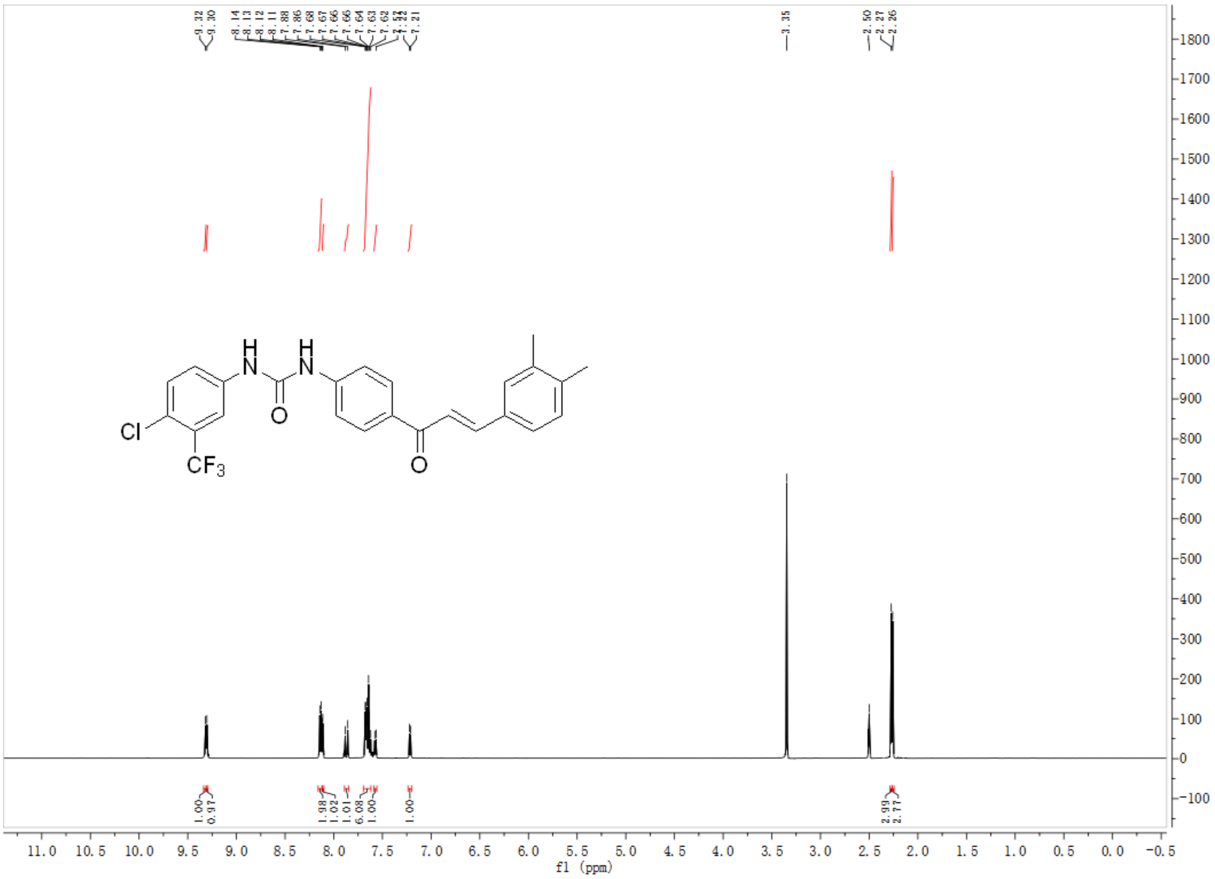

Fig S32 ^{1}H NMR (600 MHz, DMSO-d_6) spectra of **2k**

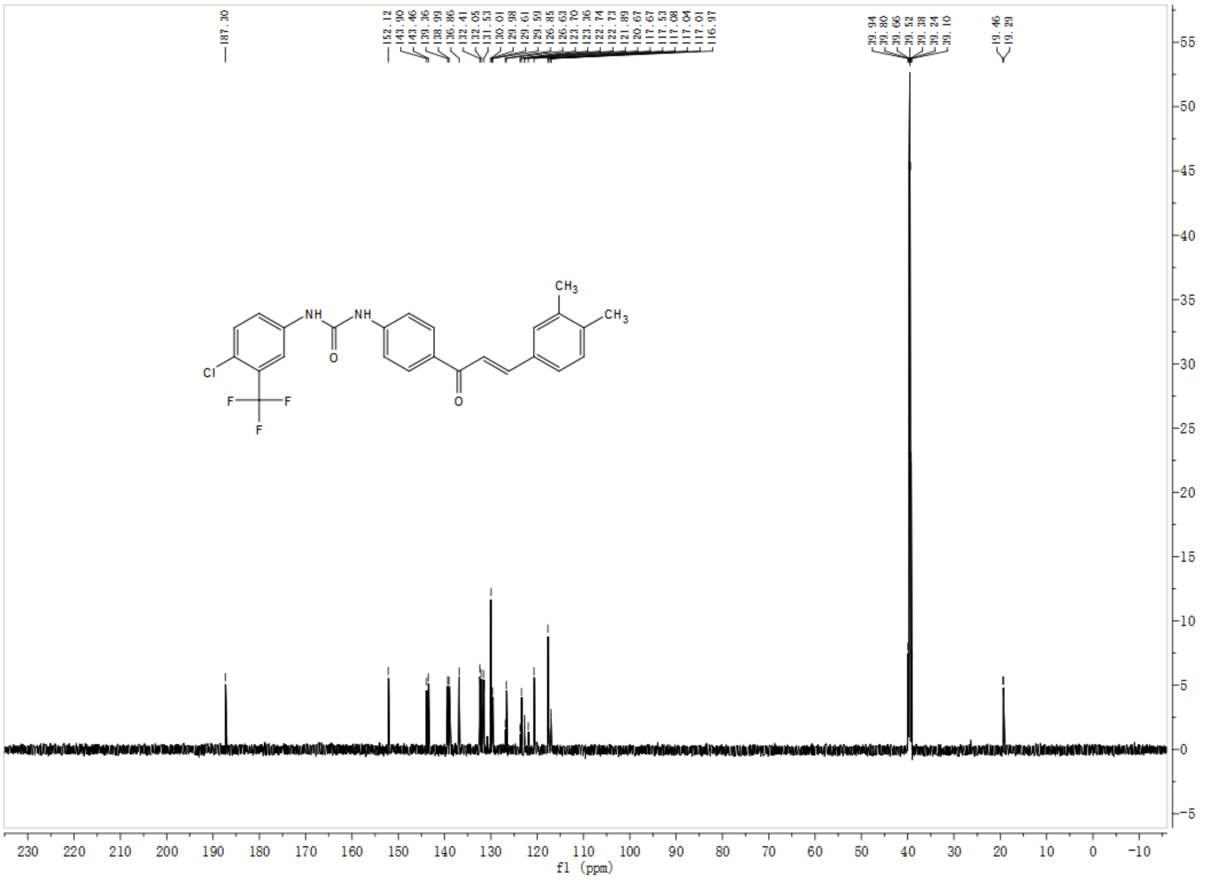

Fig S33 ^{13}C NMR (600 MHz, DMSO-d_6) spectra of **2k**

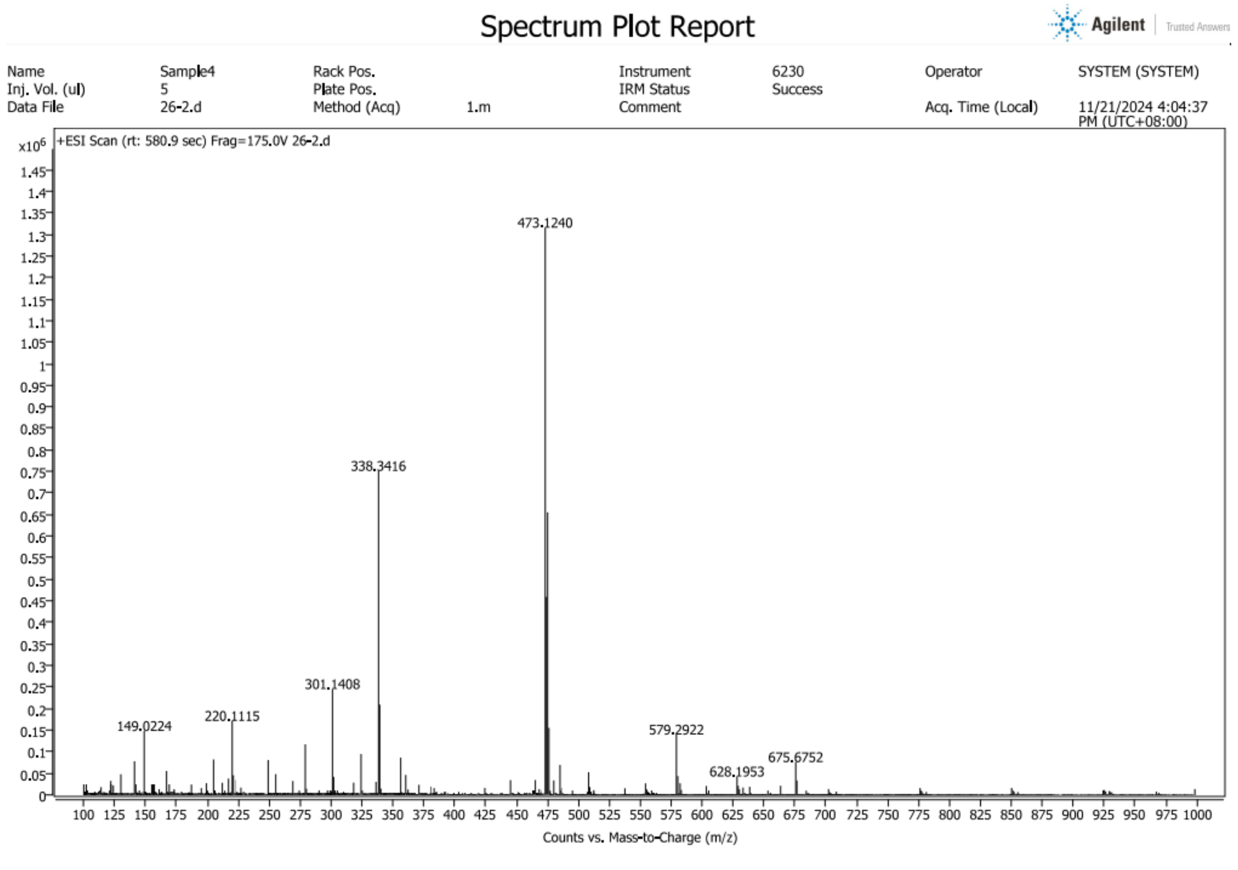

Fig S34 HR-MS spectrum of **2k**

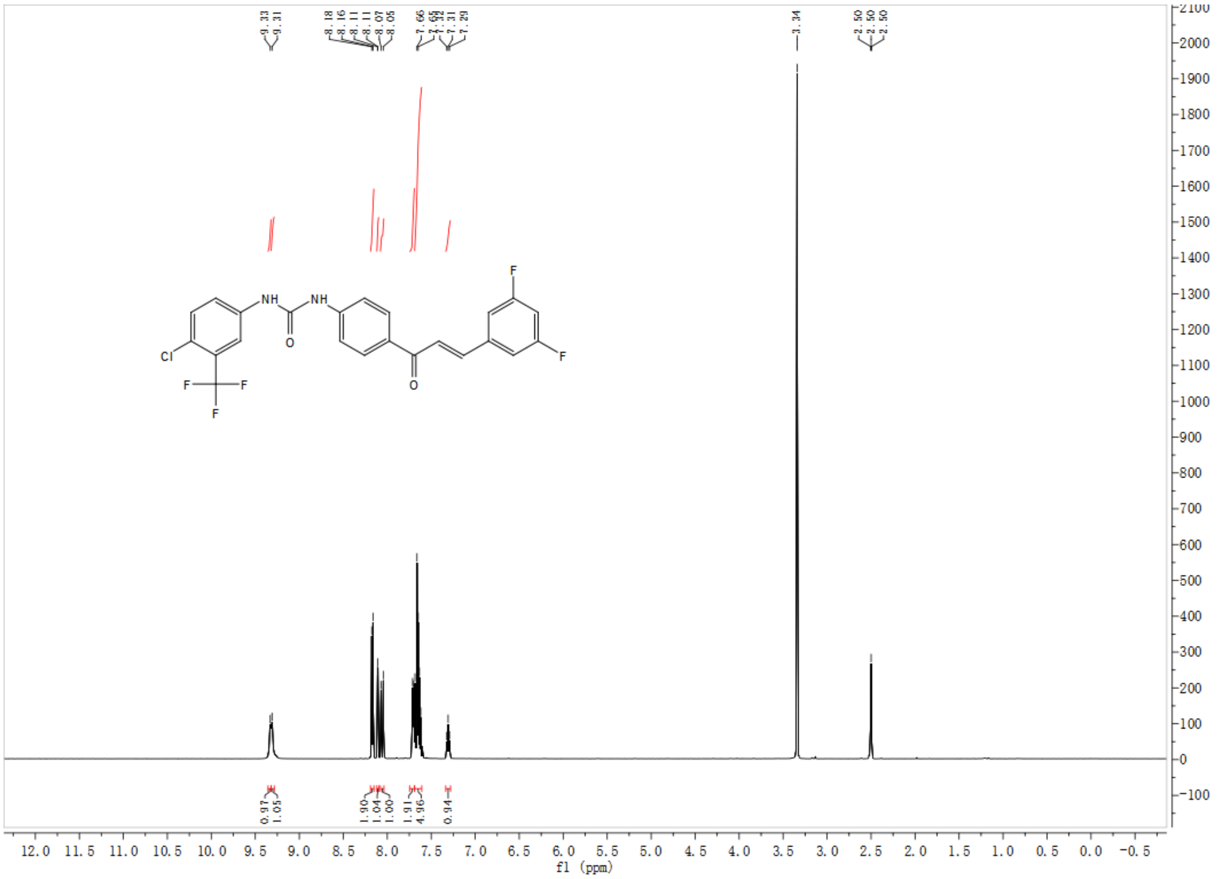

Fig S35 ^{1}H NMR (600 MHz, DMSO-d_6) spectra of **2l**

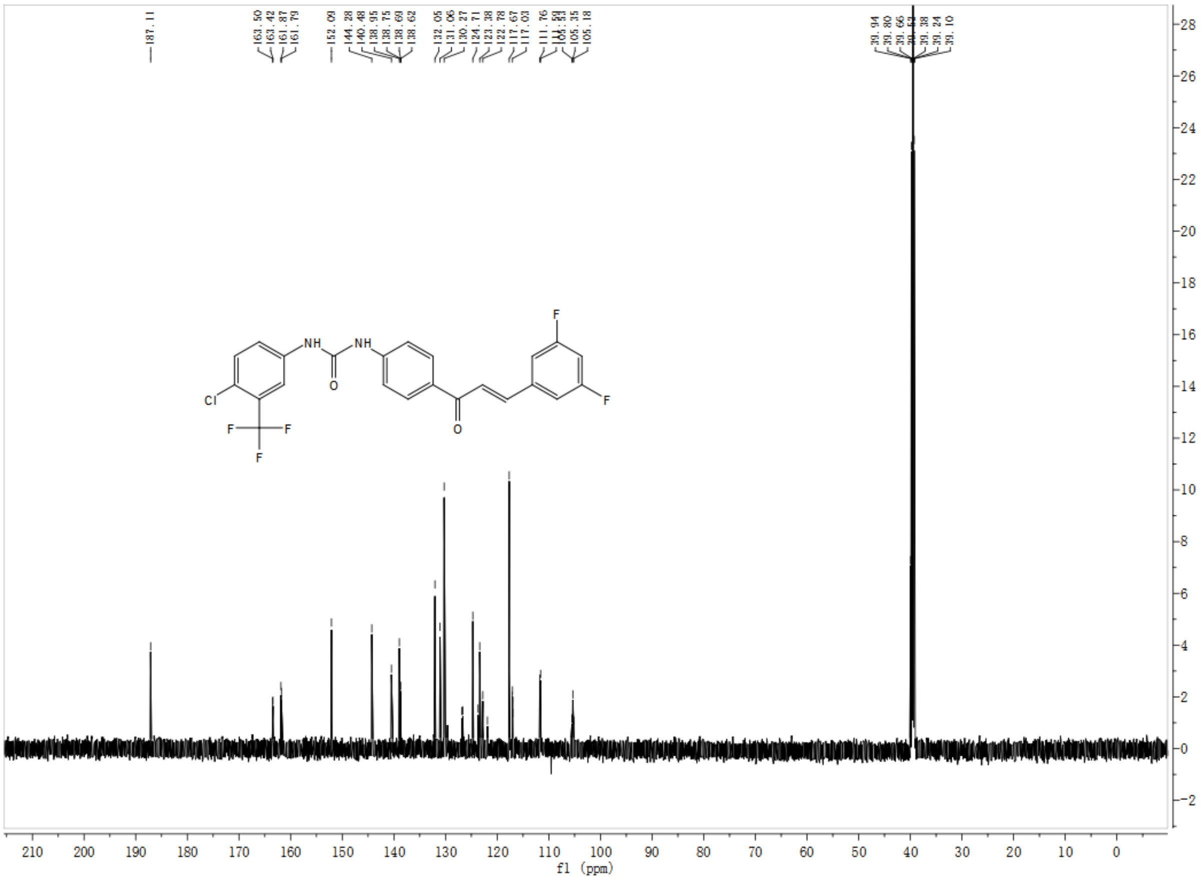

Fig S36 ^{13}C NMR (600 MHz, DMSO-d_6) spectra of **2l**

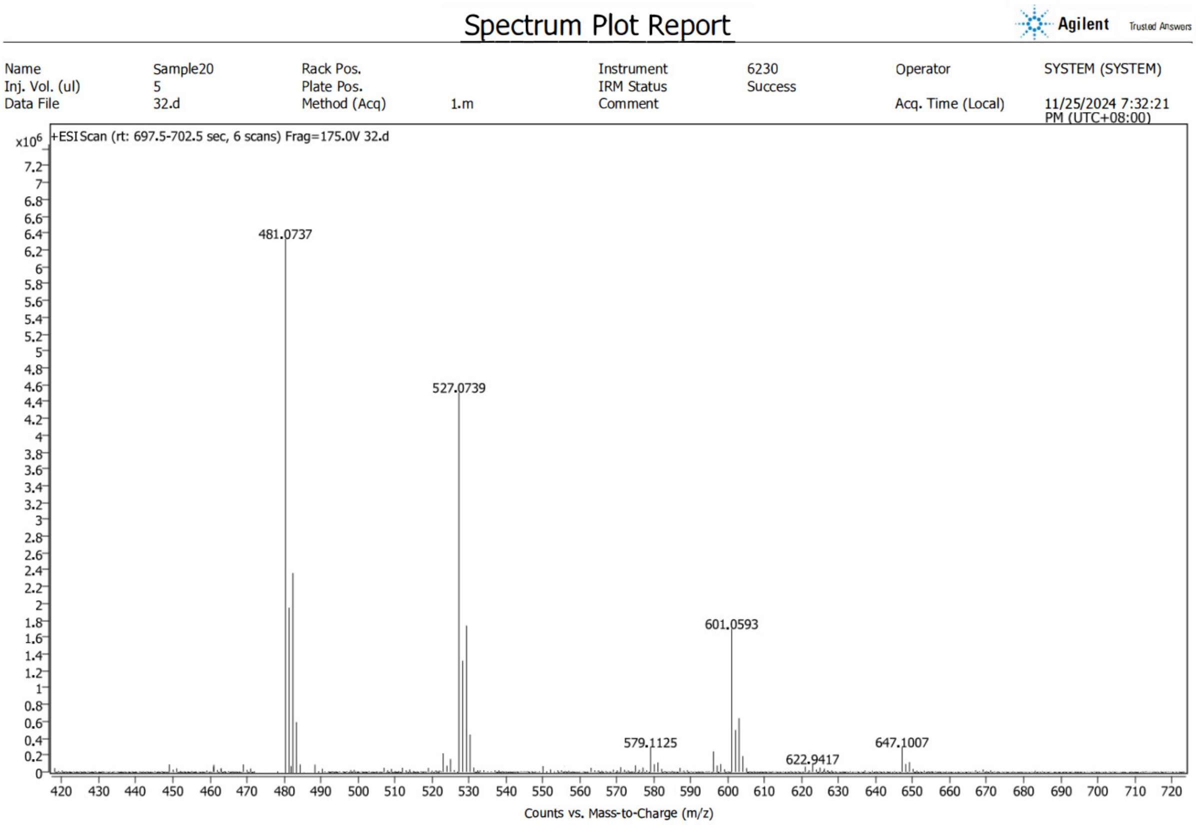

Fig S37 HR-MS spectrum of **2l**

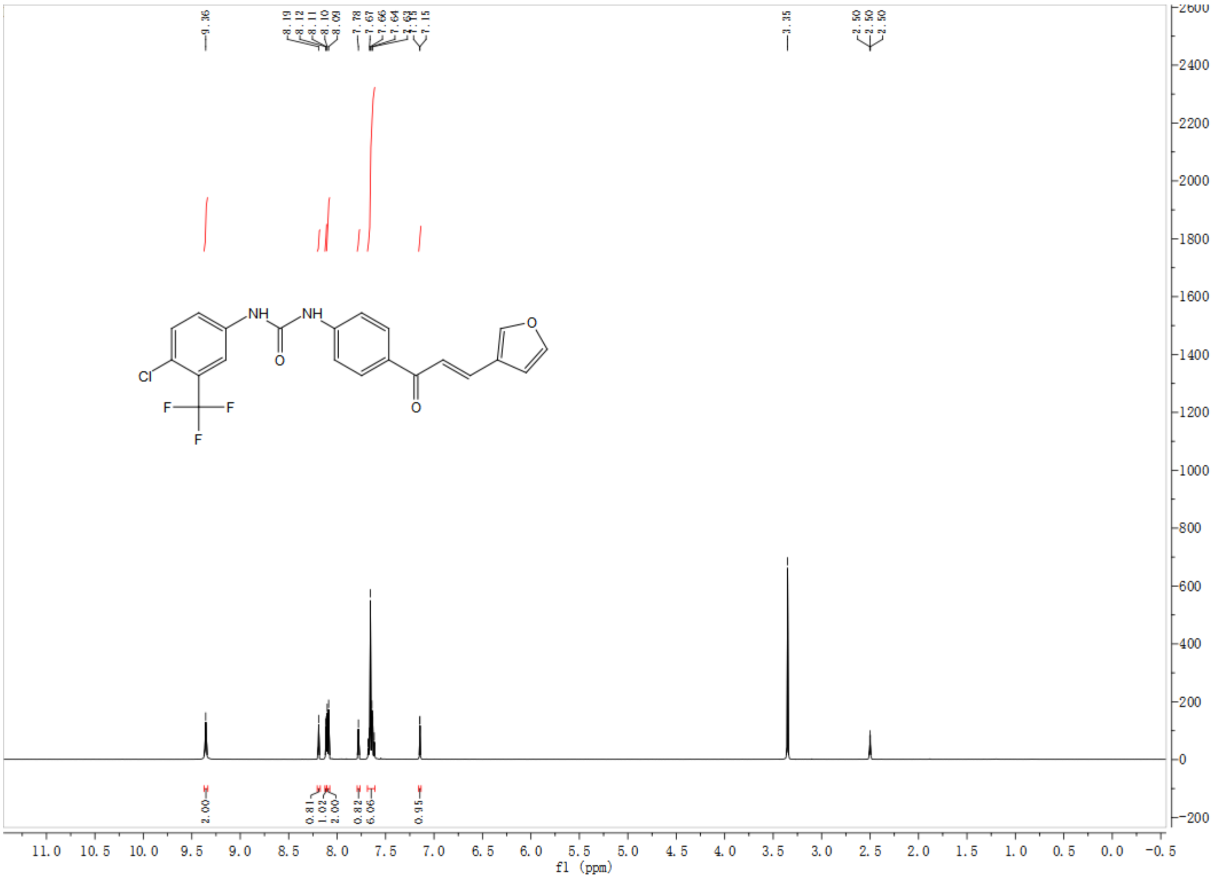

Fig S38 ^{1}H NMR (600 MHz, DMSO-d_6) spectra of **2m**

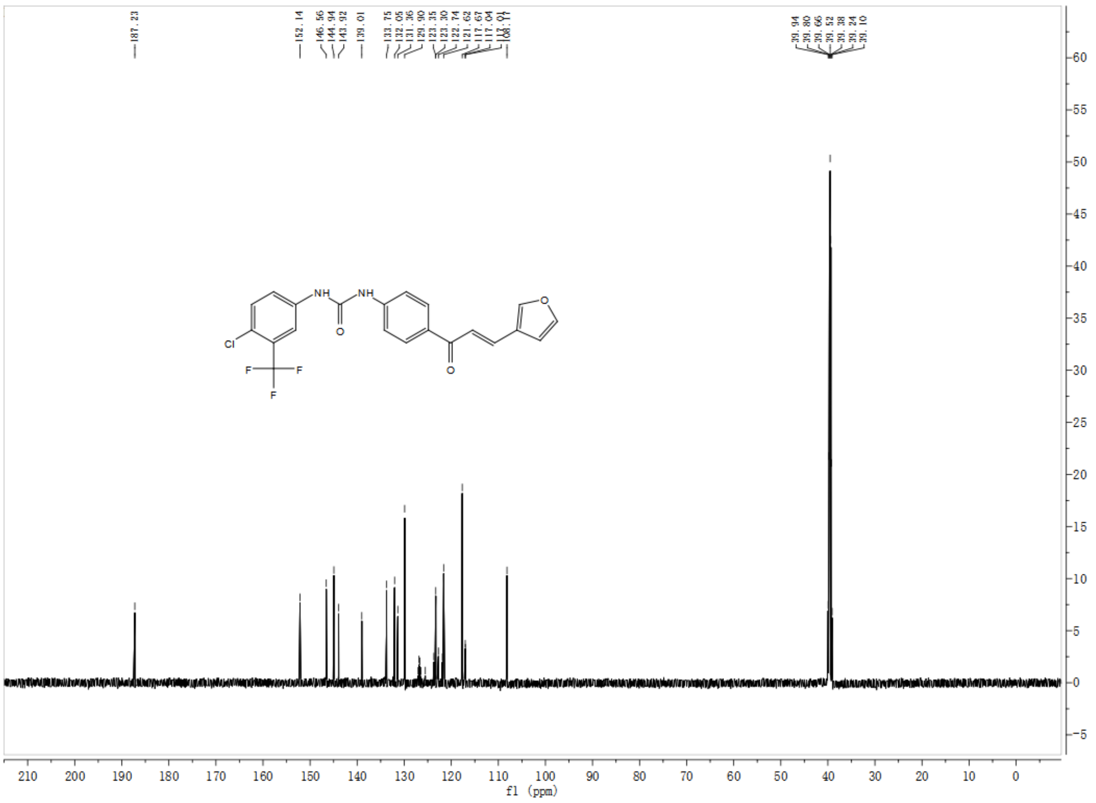

Fig S39 ^{13}C NMR (600 MHz, DMSO-d_6) spectra of **2m**

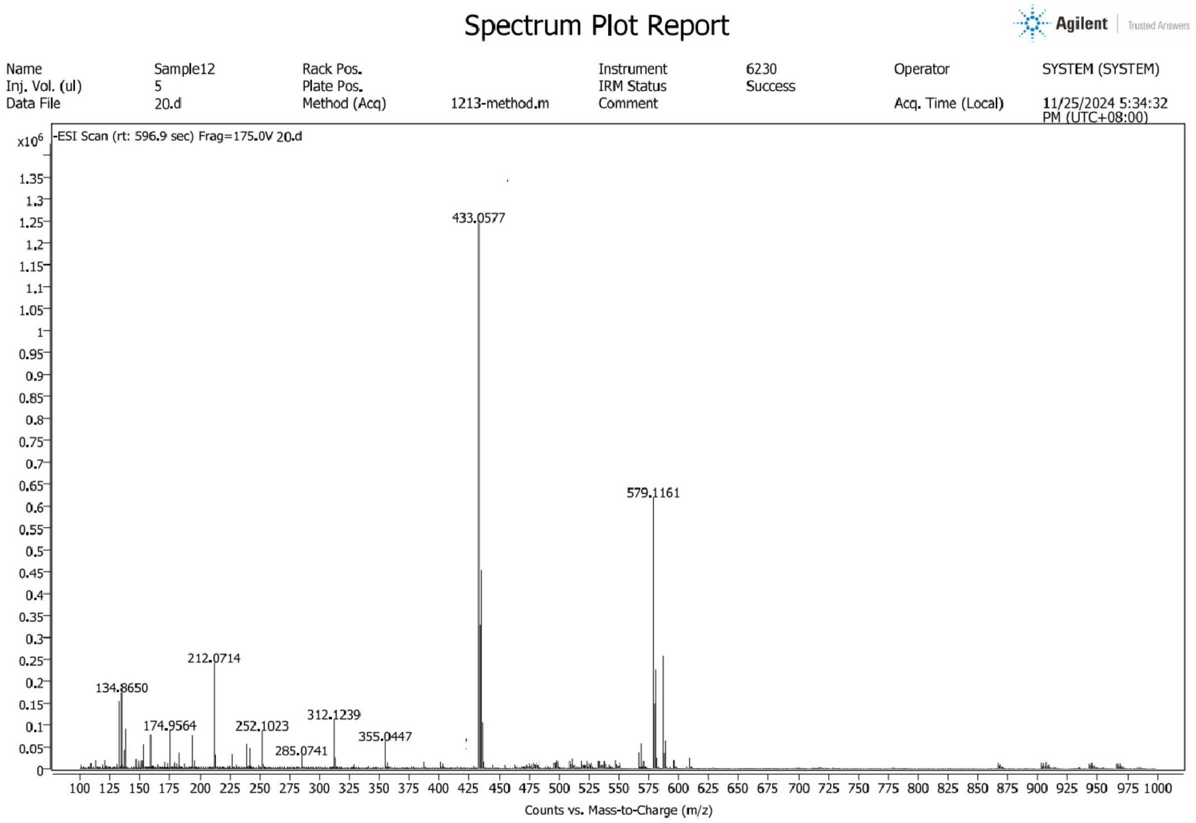

Fig S40 HR-MS spectrum of **2m**

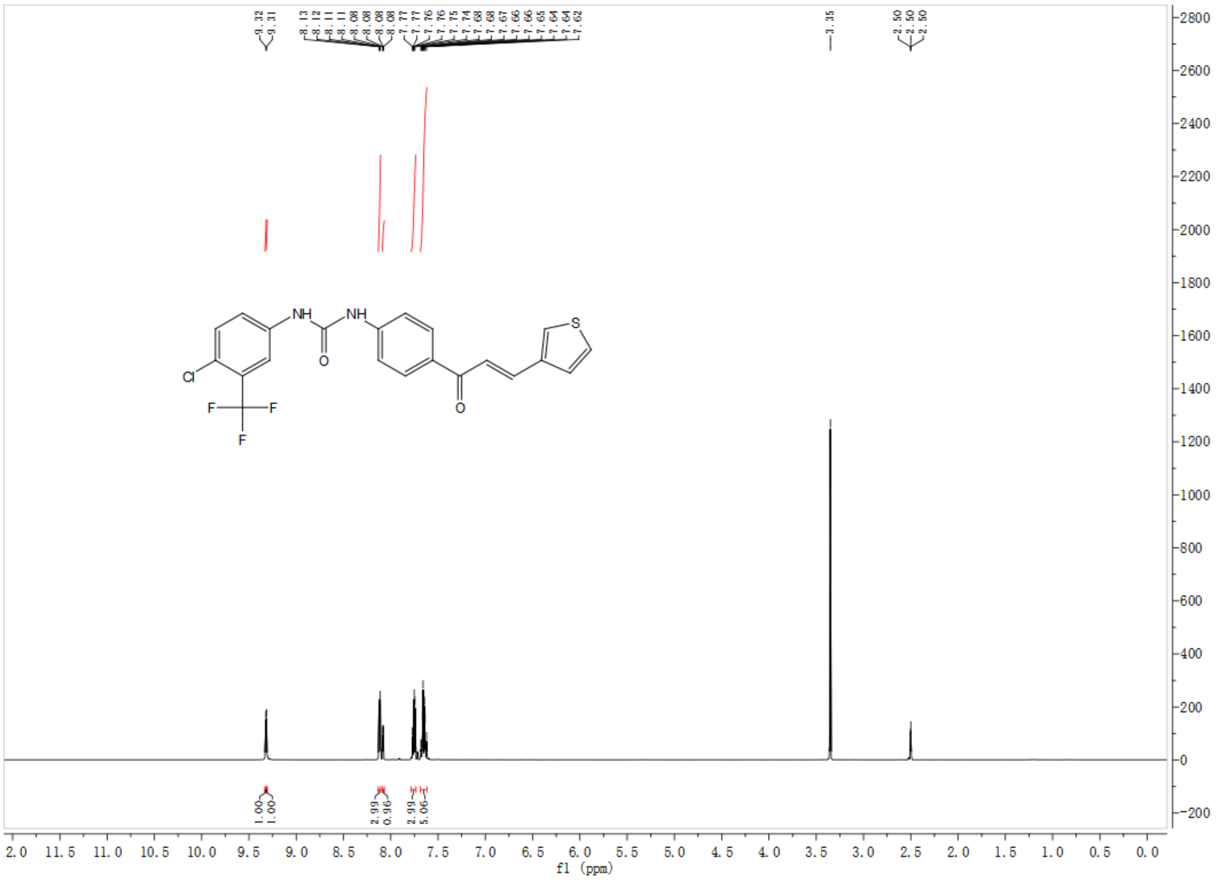

Fig S41 ^{1}H NMR (600 MHz, DMSO-d_6) spectra of **2n**

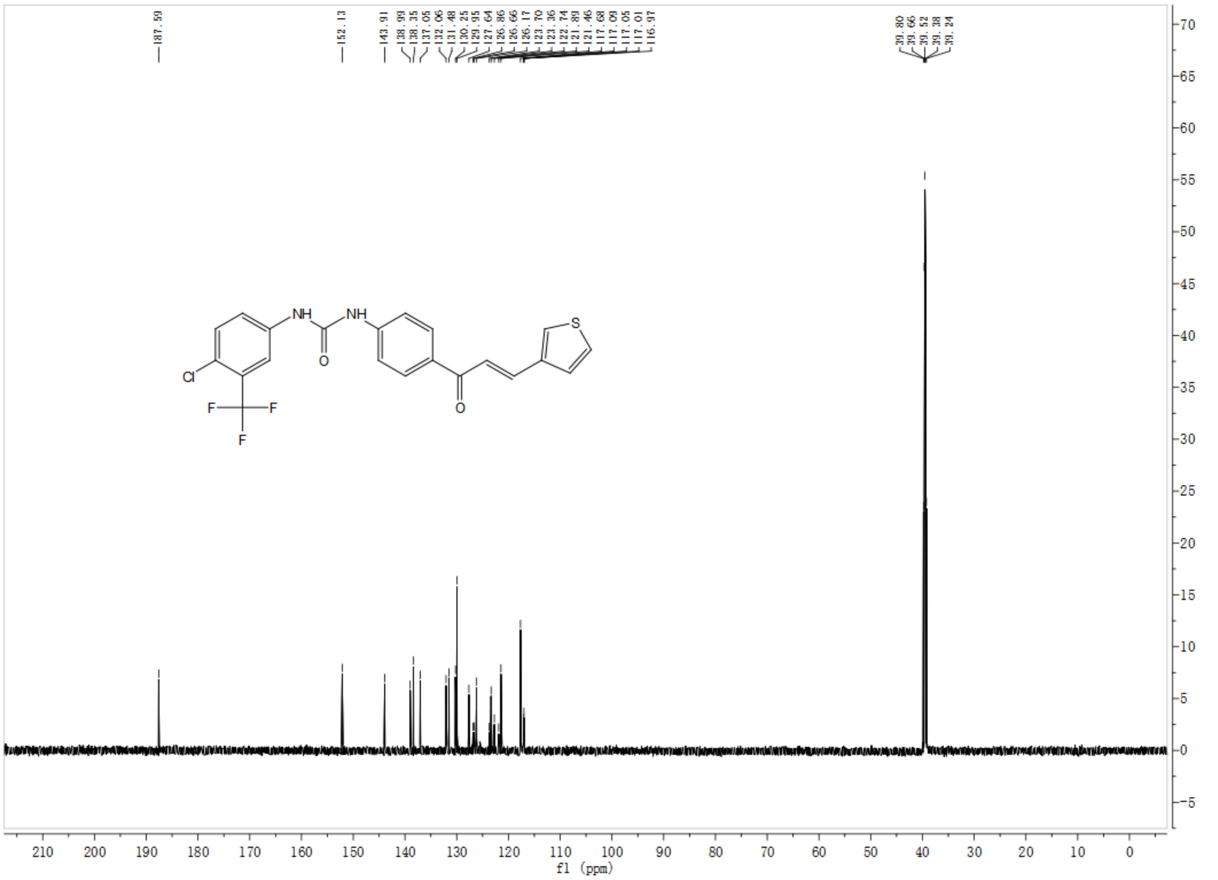

Fig S42 ^{13}C NMR (600 MHz, DMSO-d_6) spectra of **2n**

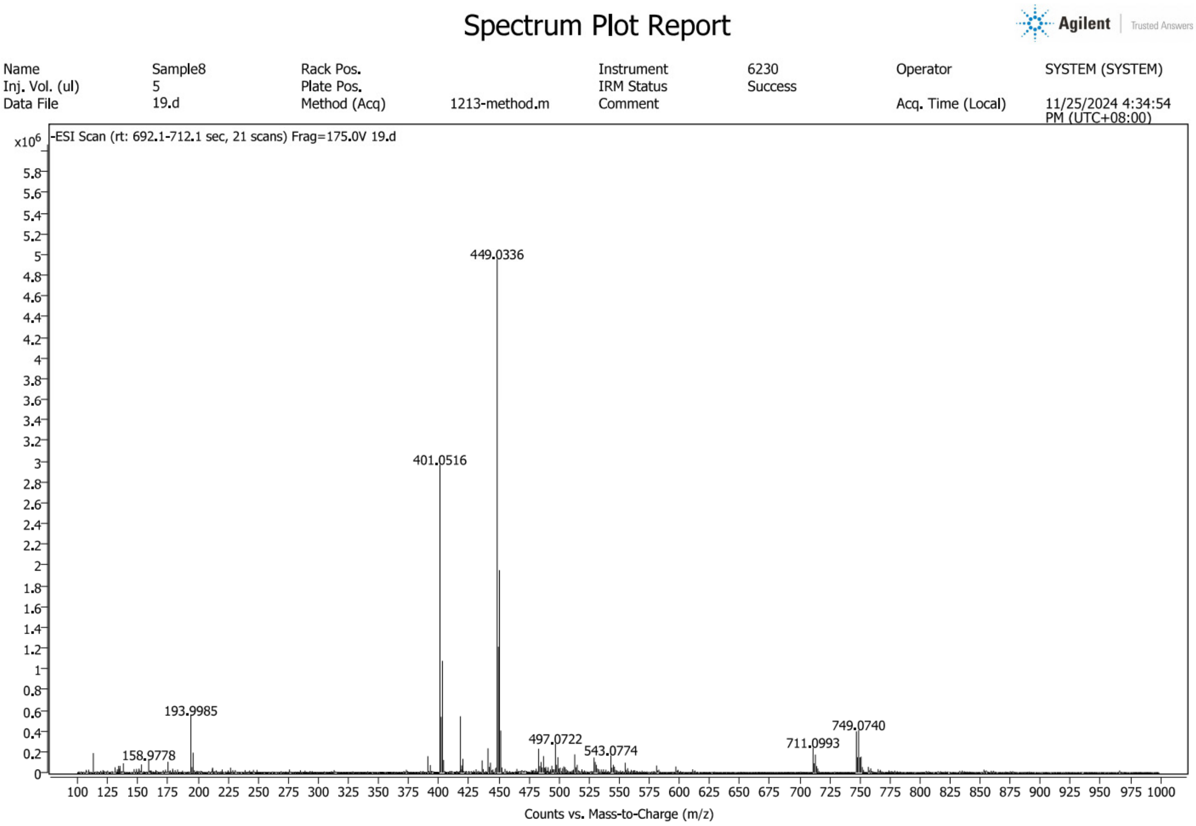

Fig S43 HR-MS spectrum of **2n**

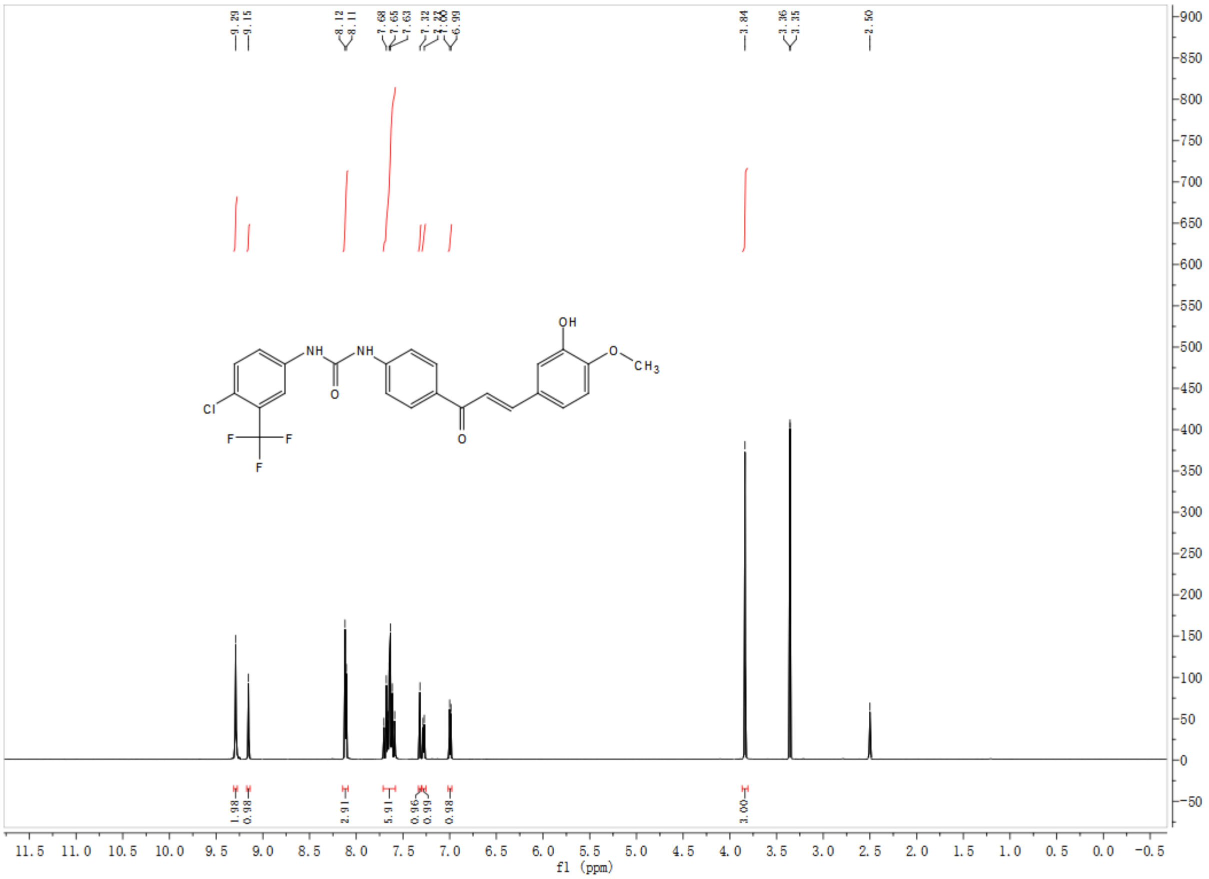

Fig S44 ^{1}H NMR (600 MHz, DMSO-d_6) spectra of **2o**

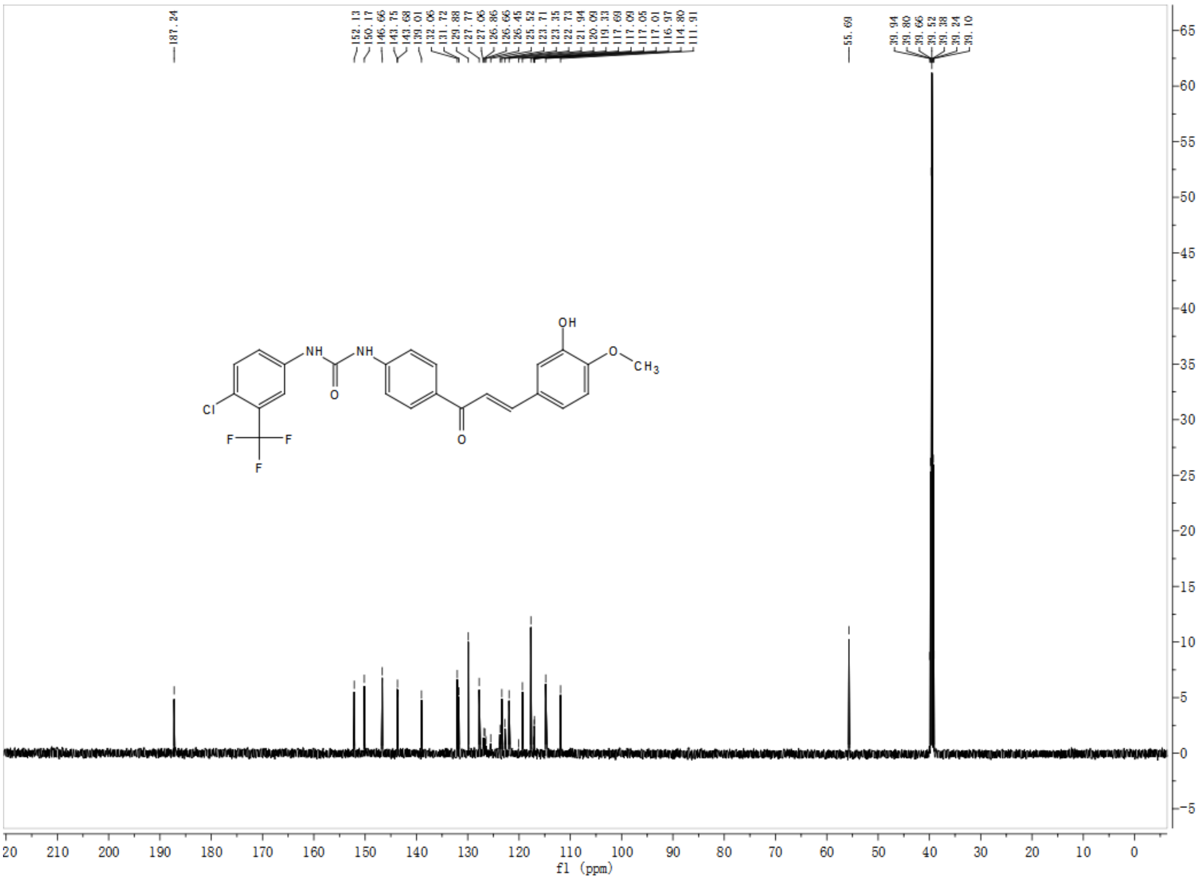

Fig S45 ^{13}C NMR (600 MHz, DMSO-d_6) spectra of **2o**

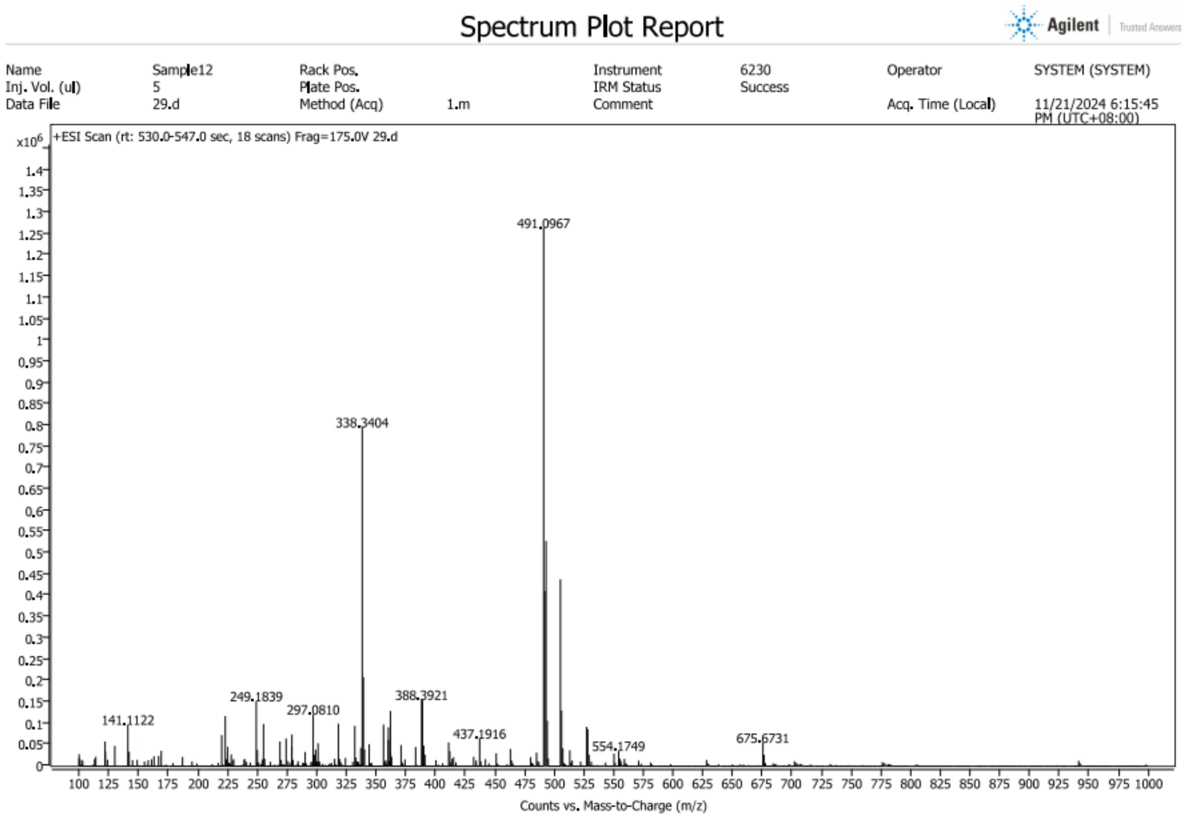

Fig S46 HR-MS spectrum of **2o**

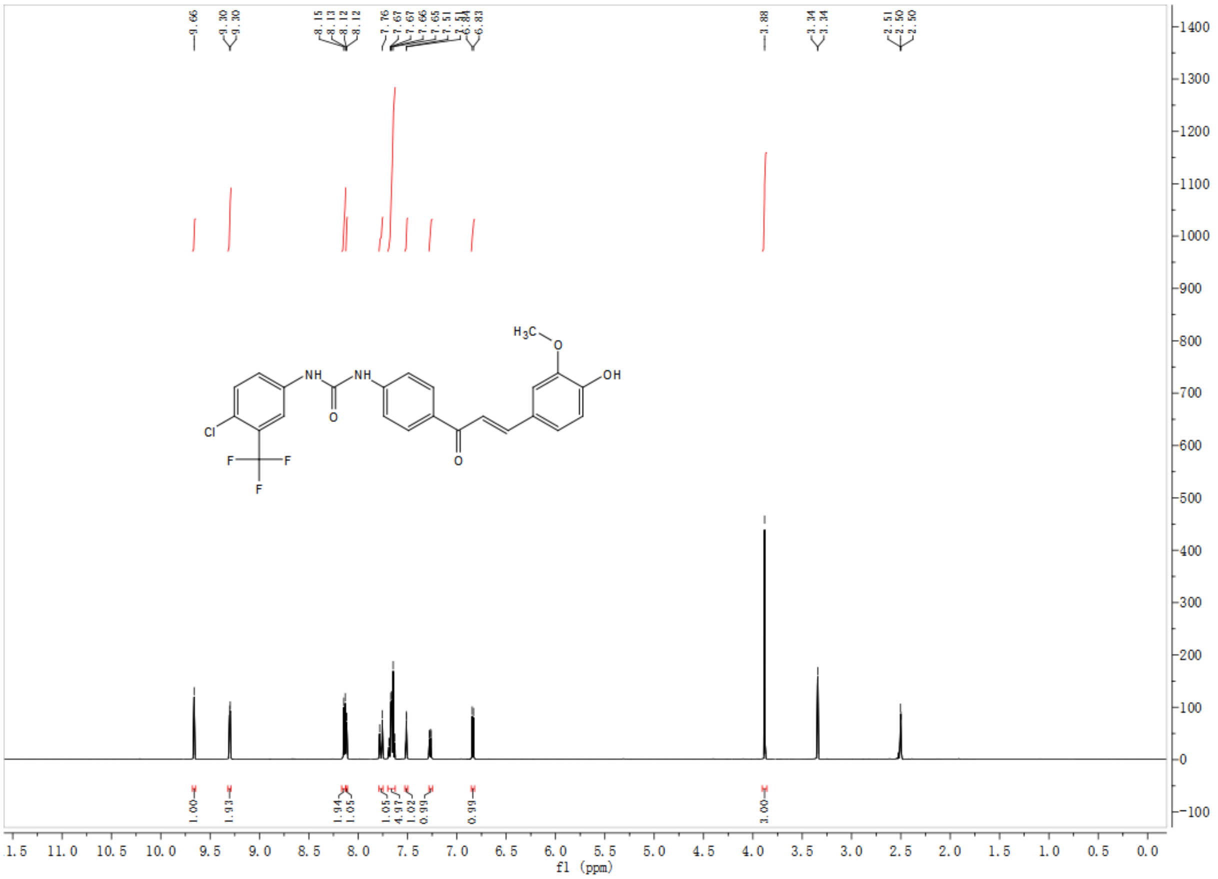

Fig S47 ^{1}H NMR (600 MHz, DMSO-d_6) spectra of **2p**

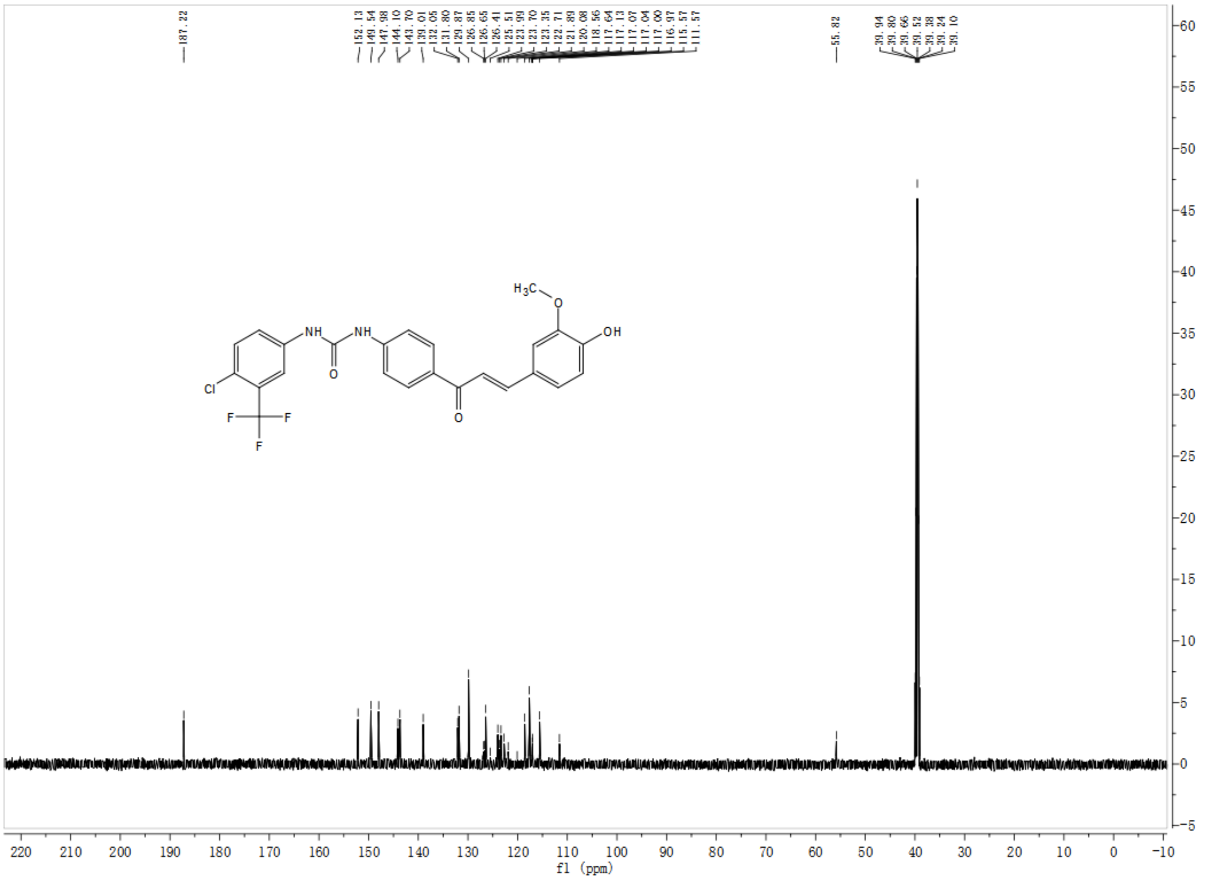

Fig S48 ^{13}C NMR (600 MHz, DMSO-d_6) spectra of **2p**

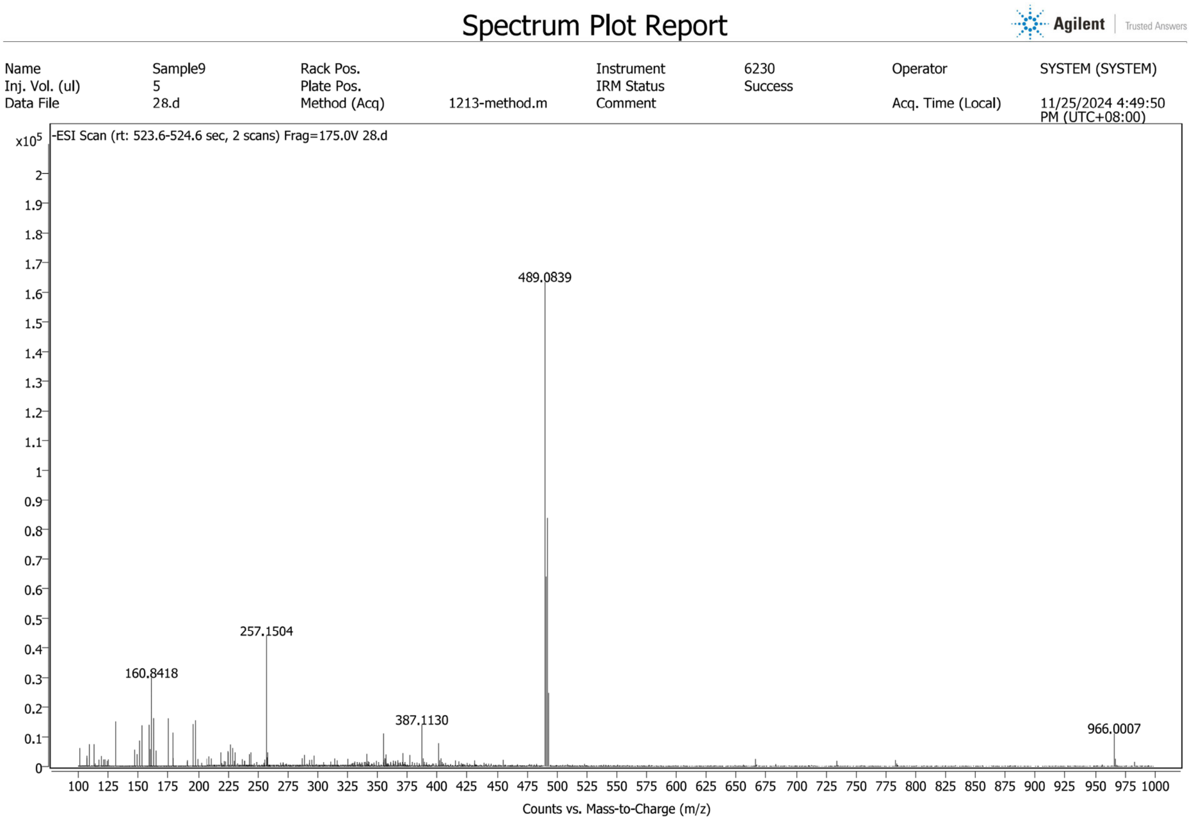

Fig S49 HR-MS spectrum of **2p**

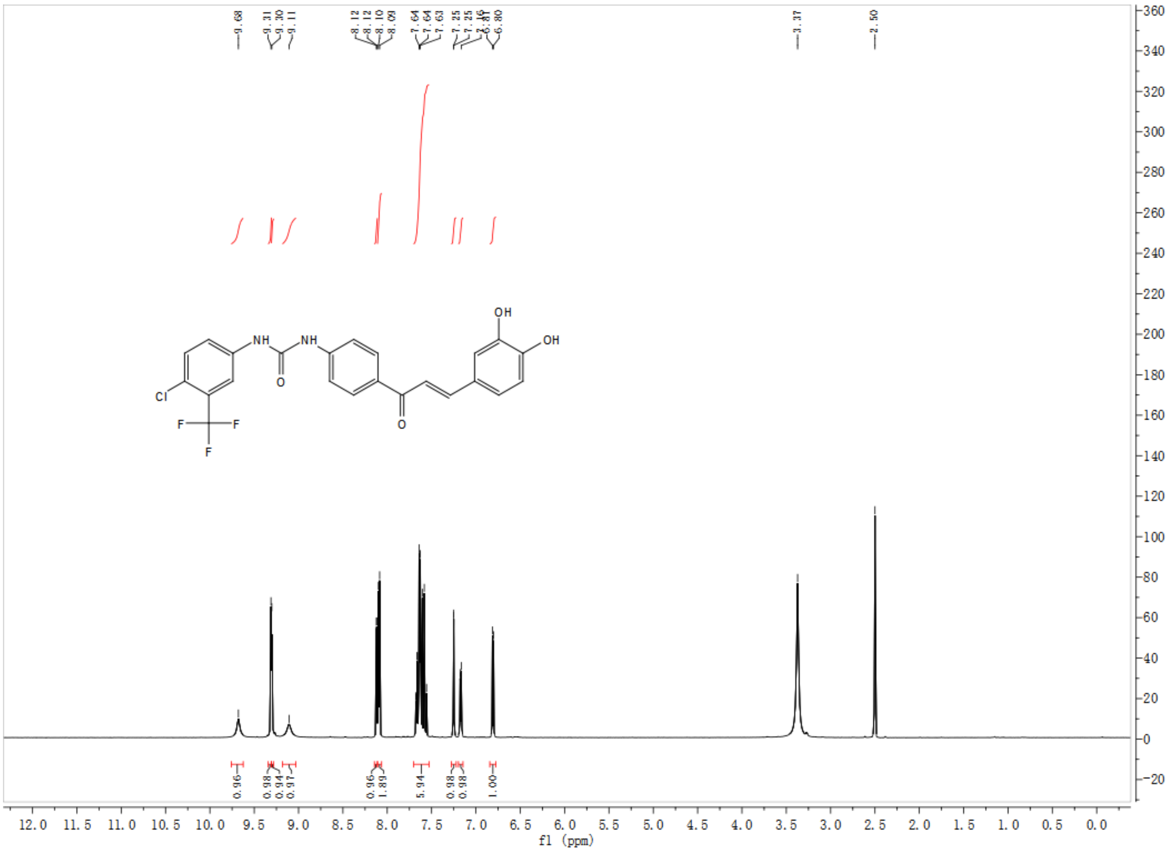

Fig S50 ^{1}H NMR (600 MHz, DMSO-d_6) spectra of **2q**

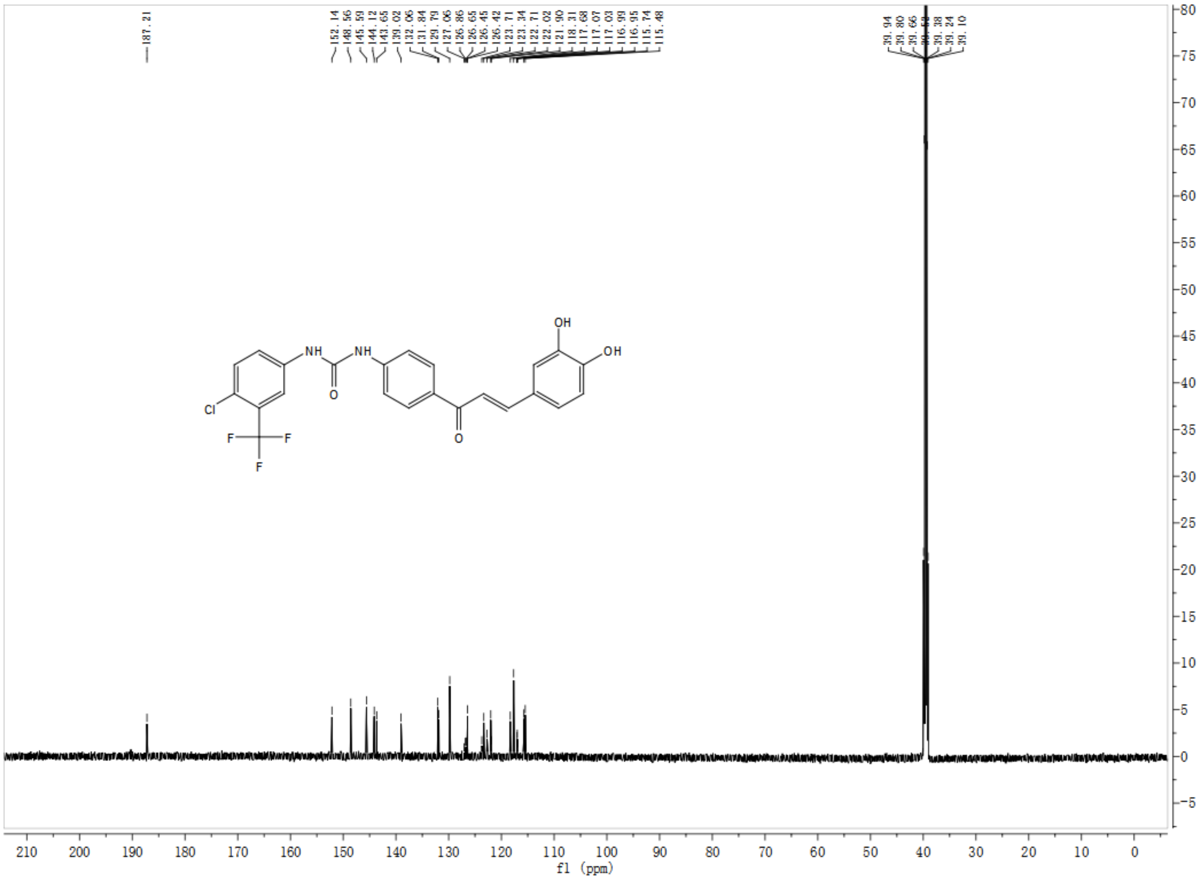

Fig S51 ^{13}C NMR (600 MHz, DMSO-d_6) spectra of **2q**

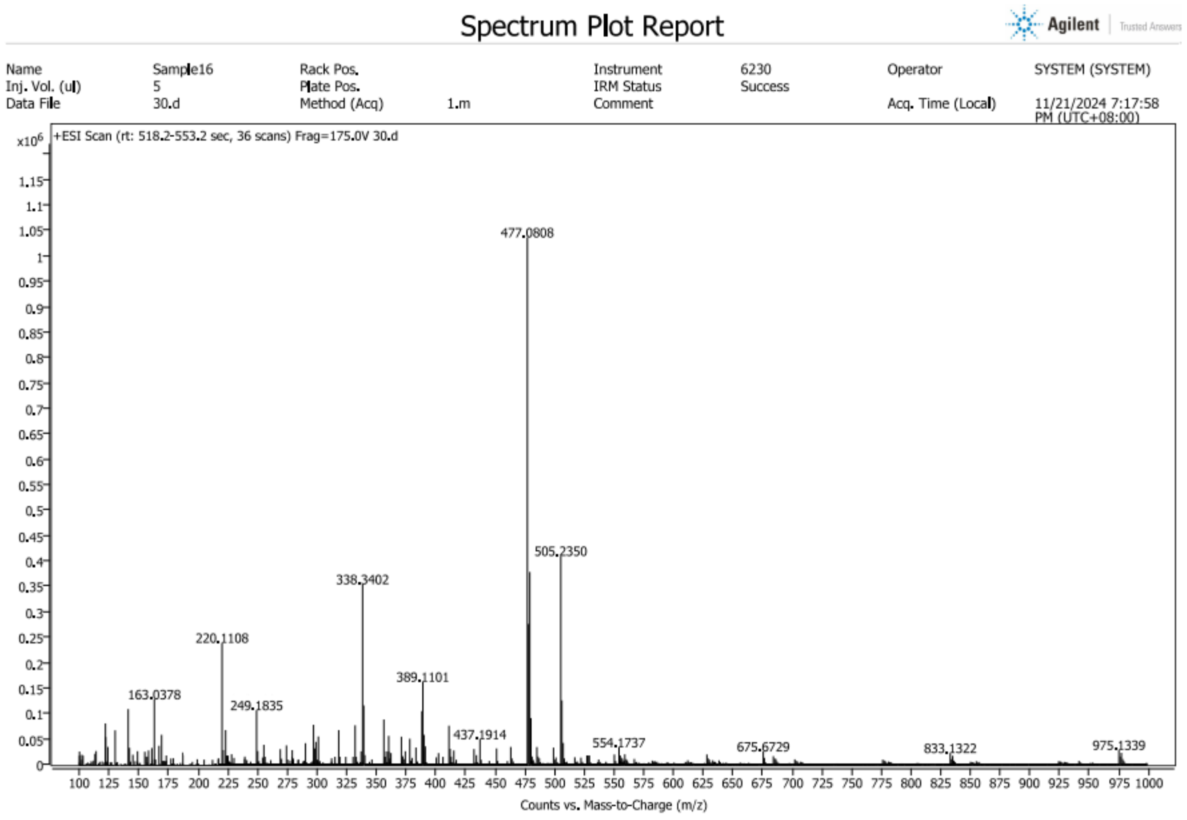

Fig S52 HR-MS spectrum of **2q**

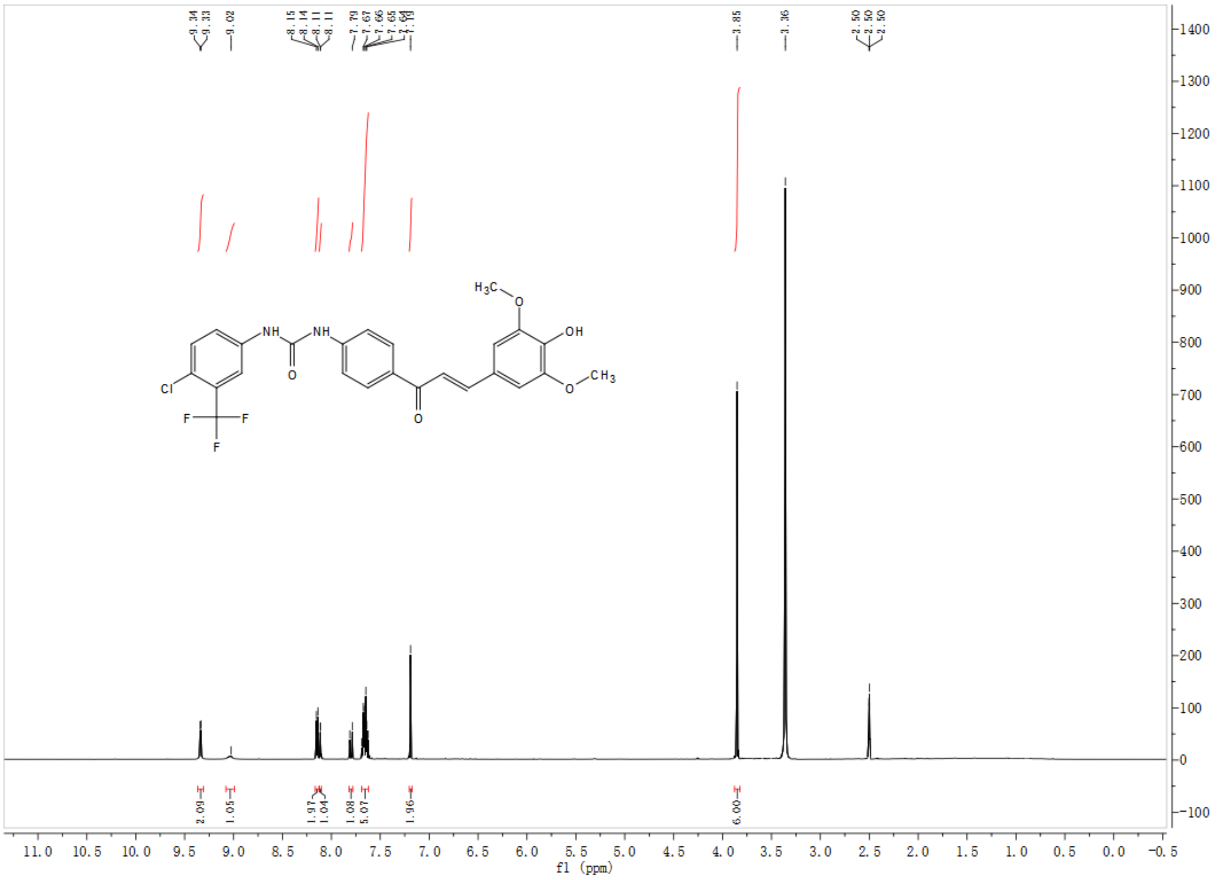

Fig S53 ^{1}H NMR (600 MHz, DMSO-d_6) spectra of **2r**

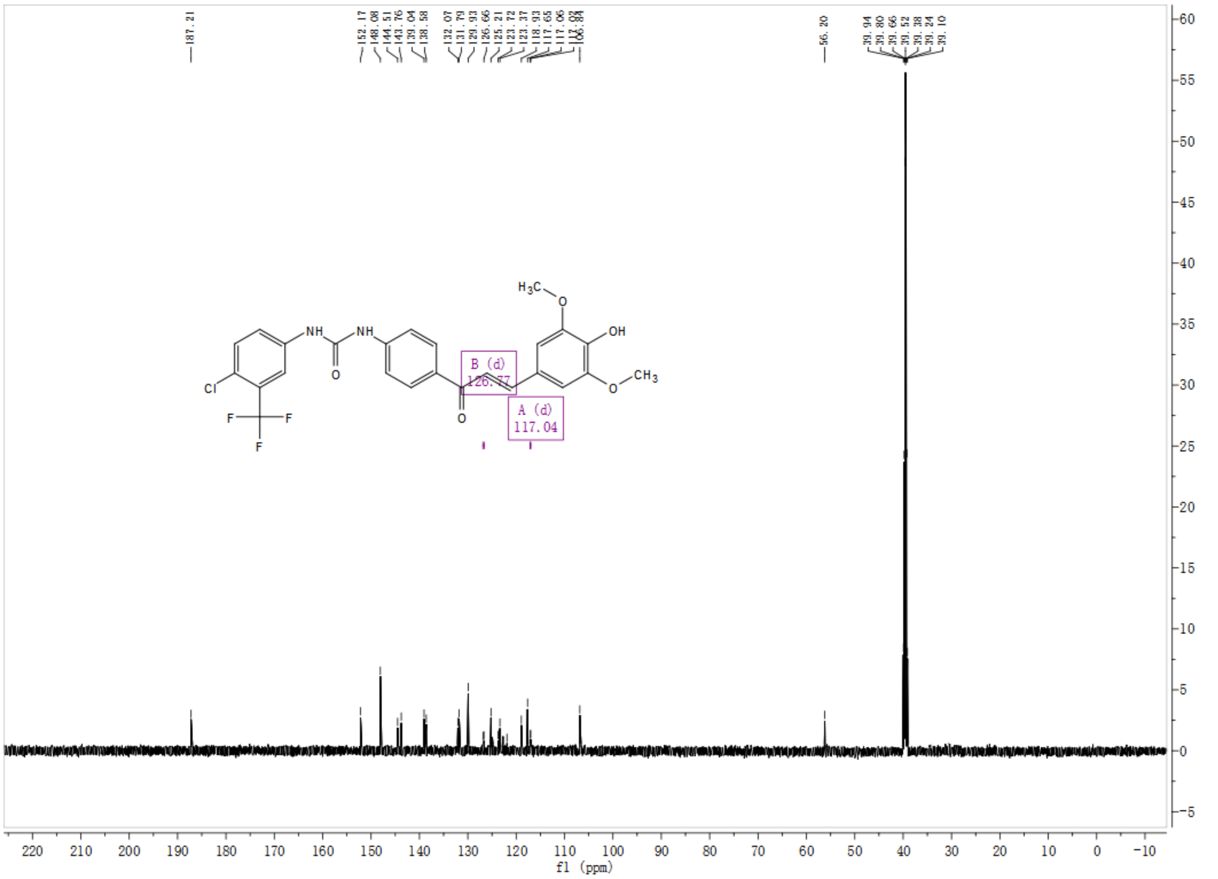

Fig S54 ^{13}C NMR (600 MHz, DMSO-d_6) spectra of **2r**

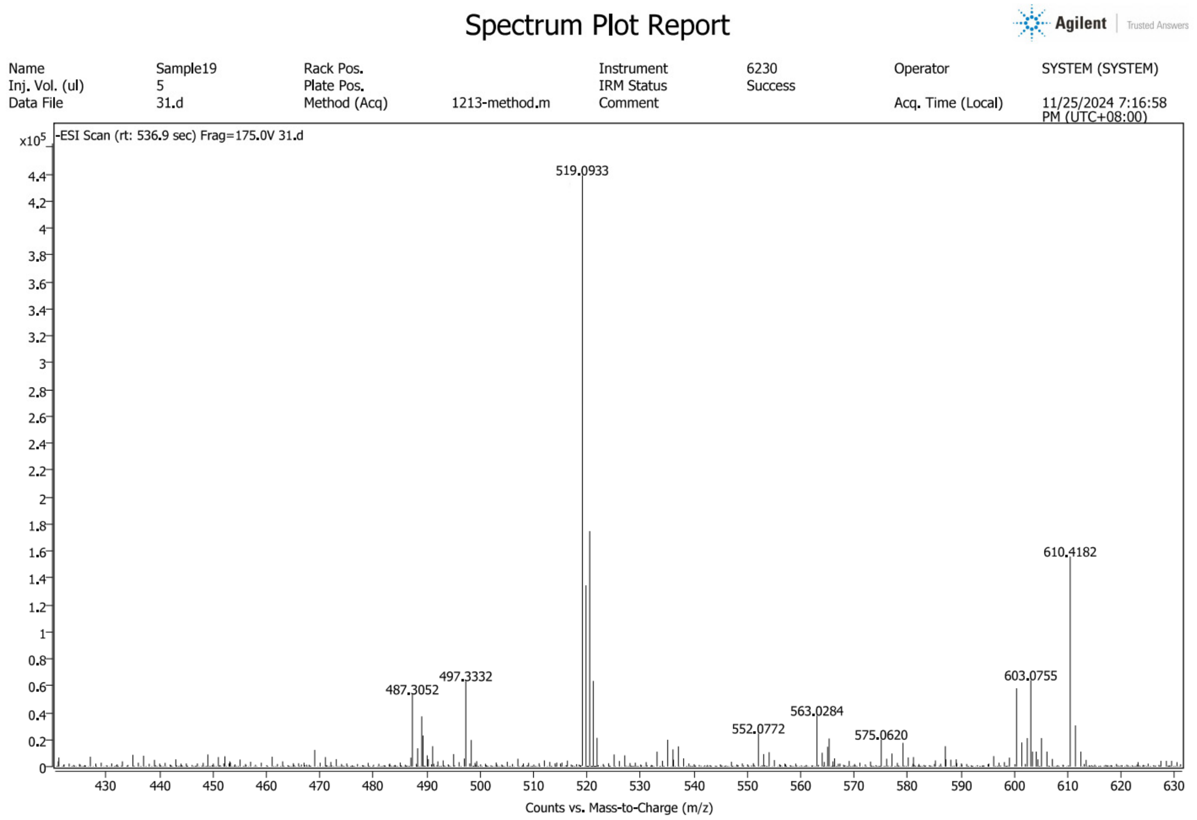

Fig S55 HR-MS spectrum of **2r**

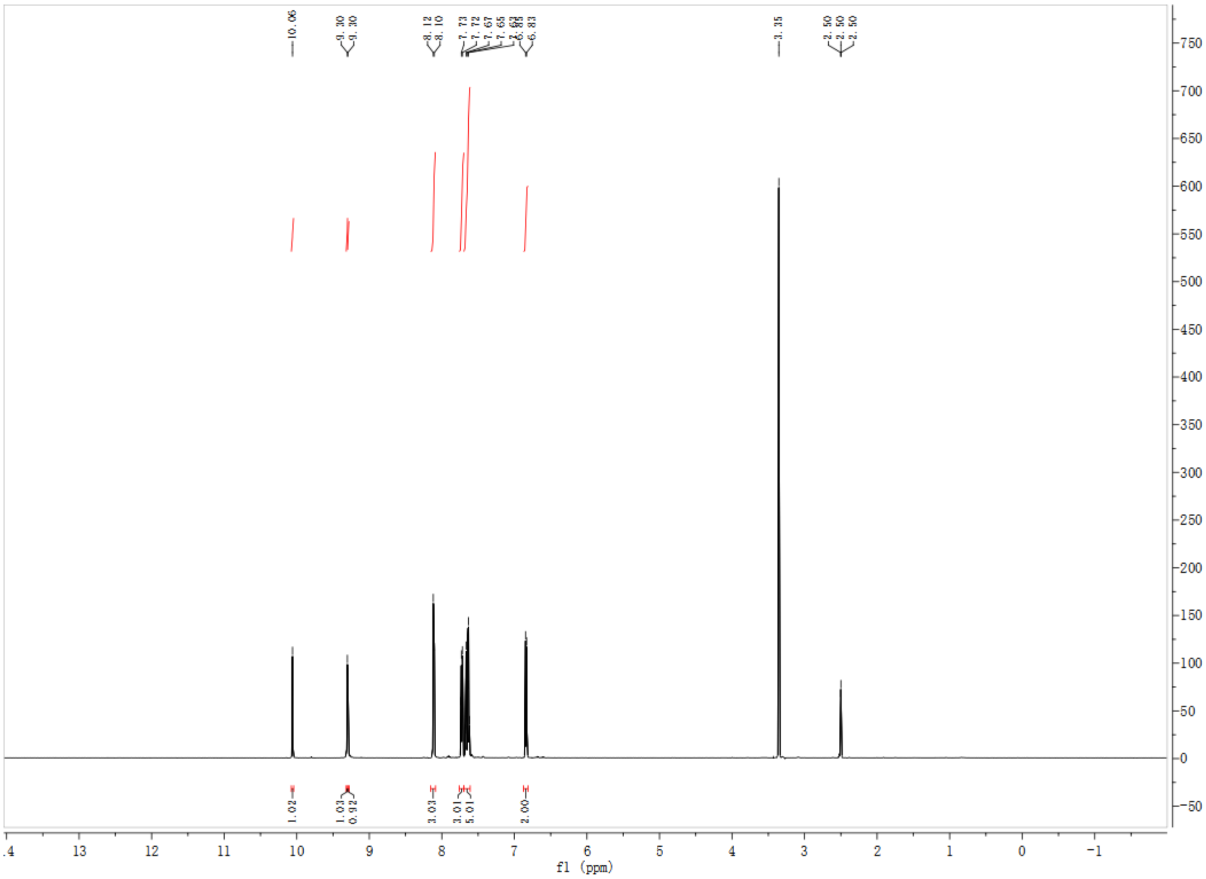

Fig S56 ^{1}H NMR (600 MHz, DMSO-d_6) spectra of **2s**

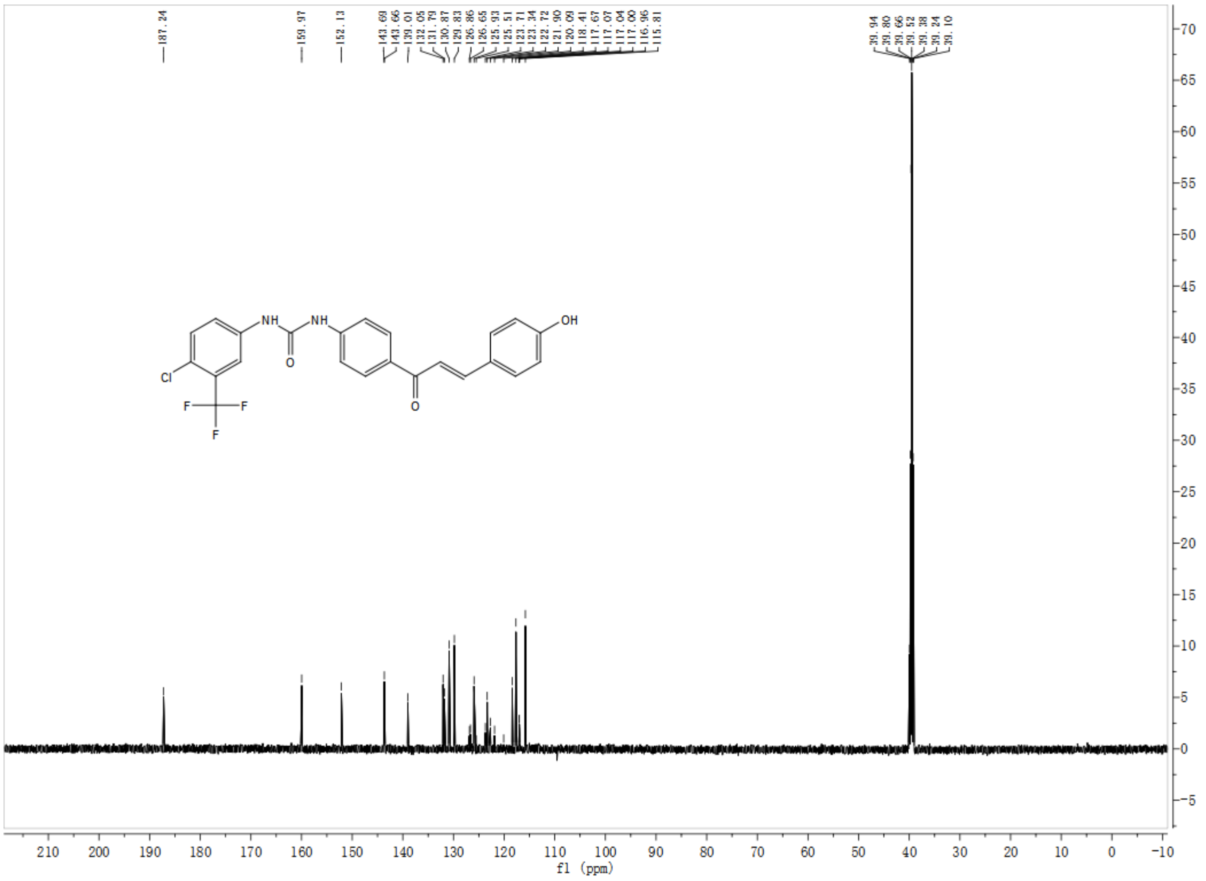

Fig S57 ^{13}C NMR (600 MHz, DMSO-d_6) spectra of **2s**

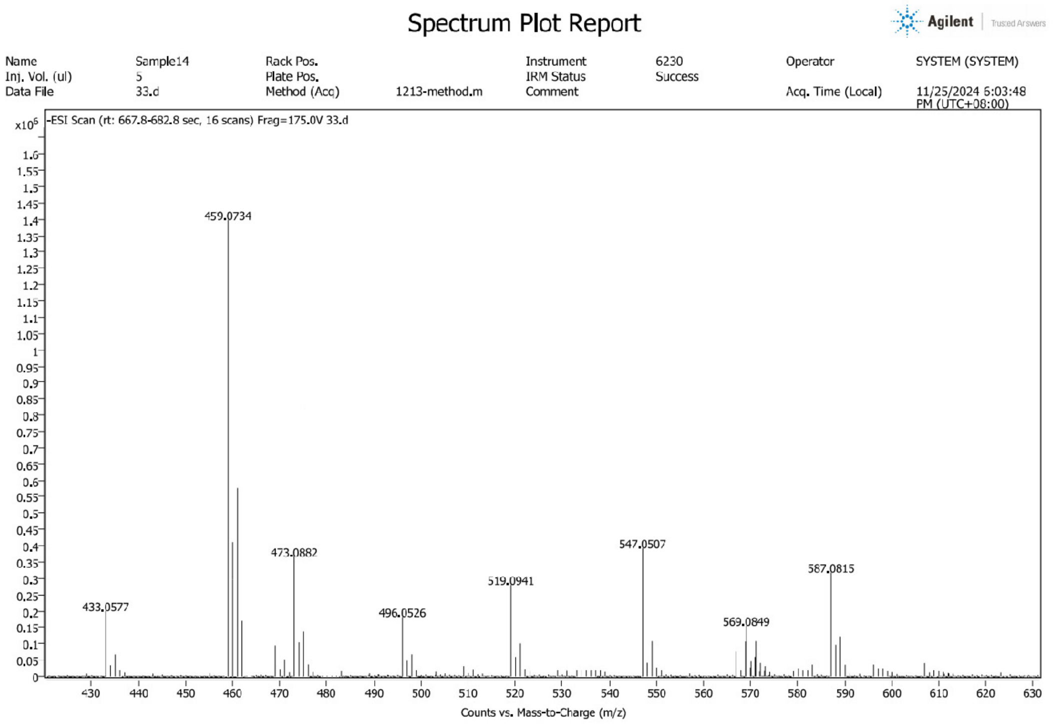

Fig S58 HR-MS spectrum of **2s**
